# Perceived barriers and facilitators to exercise adherence in osteoarthritis: A thematic synthesis of qualitative studies

**DOI:** 10.1016/j.ocarto.2025.100584

**Published:** 2025-02-15

**Authors:** Benedetto Giardulli, Davide Marazzi, Alessandro Nespoli, Gaia Leuzzi, Andrea Dell'Isola, Yeliz Prior, Simone Battista

**Affiliations:** a Department of Neurosciences, Rehabilitation, Ophthalmology, Genetics, Maternal and Child Health, University of Genova, Campus of Savona, Savona, Italy; b Department of Clinical Science Lund, Clinical Epidemiology Unit, Orthopaedics, Lund University, Lund, Sweden; c School of Health and Society, Centre for Human Movement and Rehabilitation, University of Salford, Salford, Greater Manchester, UK; d Department of Physical Education and Rehabilitation, Experimental Anatomy Research Group (EXAN), Vrije Universiteit of Brussel (VUB), Laarbeeklaan 103, 1090, Brussels, Belgium

**Keywords:** Physical therapy modalities, Physical therapy specialty, Treatment adherence and compliance, Qualitative research

## Abstract

**Objective:**

Exercise is a first-line intervention for osteoarthritis (OA). However, exercise adherence remains low, and existing studies exploring factors influencing adherence have yielded inconclusive results based on quantitative data. This study aims to synthesise qualitative studies focused on the perceived facilitators and barriers affecting exercise adherence in individuals with OA.

**Design:**

A thematic synthesis of qualitative studies was conducted. Relevant articles were sourced from MEDLINE, Cochrane Central Register of Controlled Trials, Embase, CINAHL, and PsychInfo until November 2024. Studies focused on adults (≥16 years) with hip or knee OA who had participated in exercise programmes to manage OA. The quality of the studies was assessed using the Critical Appraisal Skills Programme (CASP) tool. Data analysis followed Thematic Synthesis by Thomas & Harden to identify descriptive and analytical themes. The confidence of the evidence was evaluated through the Confidence in Evidence from Reviews of Qualitative Research (CERQual).

**Results:**

A total of 21 studies involving 458 participants were included. From seven descriptive themes, three analytical themes were developed: (i) Mind-Body Connection, (ii) Social Support Systems, and (iii) Environmental Enablers. These themes underscore the importance of personal beliefs, experiences, and mindsets, alongside social and environmental factors, in promoting or hindering exercise adherence.

**Conclusion:**

This study highlights the multifaceted cognitive, social, and environmental factors influencing exercise adherence in individuals with OA. The findings suggest that a ‘one-size-fits-all’ approach is insufficient for promoting sustained exercise engagement. Future quantitative research should build upon these insights to develop tailored strategies for enhancing exercise adherence in people with OA.

## 1 Introduction

The World Health Organisation recommends all adults undertake 150–300 ​min of moderate-intensity, or 75–150 ​min of vigorous-intensity physical activity, or a combination thereof, per week [1]. In osteoarthritis (OA), exercise is suggested as a first-line intervention in OA clinical practice guidelines (CPGs) [[2], [3], [4], [5], [6]]. However, recent evidence has questioned its effectiveness [7], with exercise adherence being a primary predictor of it [8]. Yet, people with OA often reach inadequate levels of exercise adherence [9,10]. In other rheumatic and musculoskeletal conditions, such as inflammatory arthritis, exercise adherence is generally affected by the disease's symptoms (e.g., fatigue and pain), general health, and mental health [11,12]. As with the other diseases, there is a need to identify which factors facilitate exercise adherence in OA [9,10], a population marked by unmet expectations and low care quality [13,14].

In OA, the studies examining the variables correlated to exercise response are primarily limited to quantitative secondary analyses of randomised controlled trials (RCTs) or longitudinal studies bringing inconclusive and conflictual evidence [[15], [16], [17], [18], [19]]. Exercise adherence seems to be influenced by a complex interaction of individual, social and environmental factors, such as beliefs in the exercise benefit, self-efficacy levels, previous exercise experience, tailored exercise programmes, pain, motivation, access to facilities, psychological factors, time to exercise and social support [[20], [21], [22], [23]]. These factors are complex to gather exclusively through quantitative data, with a missed opportunity to gain deeper insights from participants since investigators commonly focus on domains already covered by existing questionnaires [24]. Qualitative research methods can enrich the understanding derived from large cohort studies, offering profound interpretations of quantitative data and generating novel hypotheses that might otherwise be overlooked [24].

In the last few years, different qualitative studies have been conducted to understand barriers and facilitators to exercise adherence in people with OA. Dobson et al. (2016) conducted a scoping review to identify barriers and facilitators to exercise for people with hip and/or knee OA, emphasising the influence of individual circumstances, environmental factors, and negative beliefs [25]. However, their review included quantitative and qualitative research studies without performing a critical appraisal or assessing the quality of the evidence. Therefore, there is a need to conduct a study with a more rigorous methodology to update and appraise the latest evidence from a qualitative perspective, especially considering the emerging role of health professionals' soft skills in exercise adherence [26]. Hence, this thematic synthesis of qualitative studies aimed to synthesise the available qualitative evidence exploring the main facilitators and barriers to exercise adherence in people with OA.

## 2 Methods

### 2.1 Synthesis methodology and approach to research

We performed a pre-planned (comprehensive search strategies to retrieve all available evidence) thematic synthesis of qualitative studies to synthesise relevant articles addressing the perceived barriers and facilitators of exercise adherence in OA [27]. Thematic synthesis is a flexible, transparent and systematic method to synthesise the findings of multiple qualitative studies [27]. We followed the ‘Cochrane Handbook for Systematic Reviews for Interventions’ [28] and the ‘Cochrane Qualitative and Implementation Methods Group Guidance’ series to conduct the thematic synthesis [29,30]. We followed the ‘Enhancing transparency in reporting the synthesis of qualitative research’ (ENTREQ) to report the study [31].

### 2.2 Inclusion criteria

#### 2.2.1 Types of study

We included studies with a qualitative and mixed/multi-method (i.e. questionnaire, survey, interview, focus group, case study, observation) component. In the case of mixed/multi-method studies, we only included the qualitative part. No date and language restrictions were applied. Quantitative-design studies (e.g., RCTs) were excluded.

#### 2.2.2 Participants

We included studies involving adults (age >16 years) with hip and/or knee OA who have undergone an exercise programme. Participants self-reported an OA diagnosis made by a physician which could have been based on clinical symptoms or radiographic or were evaluated by researchers before being recruited in the exercise programme. However, the exercise programme had to be specific for hip and/or knee OA and supervised by a healthcare professional. Studies focused on arthroplasty surgery due to OA were excluded.

#### 2.2.3 Type of evaluation

We included studies focusing on the experience of an exercise programme defined by a healthcare professional to manage hip and knee OA and exploring the perceived barriers and facilitators to exercise adherence. With ‘Exercise,’ we intended any planned, structured, and repetitive physical activity [32]. We did not include studies on unplanned physical activity (e.g., dog walking).

### 2.3 Data sources

Five electronic databases (MEDLINE, Cochrane Central Register of Controlled Trials, Embase, CINAHL and PsychInfo) were searched until November 2024. Given the lack of consensus on which databases should be used for qualitative synthesis, we followed the ‘Cochrane Handbook for Systematic Reviews of Interventions’ recommendations [28]. According to their guidelines, the minimum requirements include using MEDLINE via PubMed, EMBASE, and the Cochrane Library. Additional sources should be selected based on the specific review topic. Consequently, we also included CINAHL and PsycINFO, as they are leading databases for qualitative and psychological primary studies.

### 2.4 Electronic search strategy

The search strategy was based on the SPIDER (Sample, Phenomenon of Interest, Design, Evaluation, Research type) framework for qualitative evidence synthesis and carried out by five authors (SB, DM, AN, BG, GL) [33]. The search strings are reported in Supplementary Material 1. The authors also manually searched the reference lists of the included articles.

### 2.5 Study screening methods

Articles obtained from the research were uploaded to Covidence (Covidence systematic review software, Veritas Health Innovation, Melbourne, Australia. Available at www.covidence.org). Duplicates were removed by Covidence. Two independent authors (DM and GL) read the studies’ titles and abstracts and then included the studies based on the inclusion and exclusion criteria. In case of disagreement, a third author (SB) was consulted. Full-text screening was done by splitting the list of articles in two, with each half being analysed by two separate groups composed of two independent authors (DM ​+ ​GL and AL ​+ ​BG). The final selection was made through discussion to clear any disagreement involving SB. These groups were maintained over time throughout the study for the subsequent phases.

### 2.6 Data extraction

Four authors, divided into the abovementioned groups, independently extracted the data from each study. In particular, data were organised in a standardised table focusing on author, title, year, country, study design and analysis, sampling strategy, population, intervention, and clinical characteristics. Subsequently, the two pairs independently collected the relevant themes and subthemes, along with their citations, in an additional table, from the included studies. Disagreements in the data collection were resolved through a consensus process or consultation with a fifth author (SB).

### 2.7 Rationale for appraisal and appraisal items

According to the Cochrane Qualitative and Implementation Group recommendations, the studies were evaluated for critical appraisal using the Critical Appraisal Skills Programme (CASP) tool by two pairs of authors as grouped above [30]. In case of disagreement, SB was consulted. CASP is widely used to assess the quality of health-related qualitative syntheses. This tool consists of a ten-question checklist in which you are asked to record a “yes”, “no”, or “can't tell”, and assist you in appraising qualitative research, considering what the results of a study, if the results of the study are valid and if the results help locally. Additionally, there are “comments” boxes provided to explain the reasoning behind the answers given, along with suggested “hints” to aid researchers in determining the correct response.

The Confidence in Evidence from Reviews of Qualitative Research (CERQual) was used by three authors (GB, DM and AN) to evaluate the confidence of the results, with SB reviewing the process. This approach includes the methodological limitations, relevance, coherence and adequacy of the data [34]. The methodological limitations were based on the previous evaluation with the CASP. GB, DM and AN assessed the relevance as the extent to which the context or inclusion criteria of the primary studies supporting the review findings applied to the context specified in the review question; coherence referred to the fit existing between the primary study data and the synthesised findings in the review, while data adequacy was determined based on the degree of richness and quantity of data supporting a review finding [34].

### 2.8 Data synthesis

The qualitative studies were synthesised using ‘Thematic Synthesis’ by Thomas and Harden [27]. There are three phases of thematic synthesis: ‘line-by-line’ text coding, ‘descriptive theme’ development, and ‘analytical theme’ generation. Whereas the development of descriptive themes remains close to the primary studies, the analytical themes indicate a stage of interpretation where authors ‘reach beyond’ the primary research and produce new interpretive constructs, explanations, or hypotheses to answer the study's research question. In particular, three independent authors (DM, AN and BG) coded study texts line-by-line based on the content and meaning of the various quotations in the included articles, with SB overseeing the whole process. Then, the codes were independently, methodically, and inductively arranged to provide descriptive themes that closely matched the data collected from the original studies. Finally, the descriptive themes were further synthesised to generate the final analytical themes focused on our research question about facilitators and barriers to exercise adherence in OA. The codes and theme development underwent multiple reviews and iterations. All authors discussed and decided on the final themes.

## 3 Results

### 3.1 Study selection

**Fig. 1:**
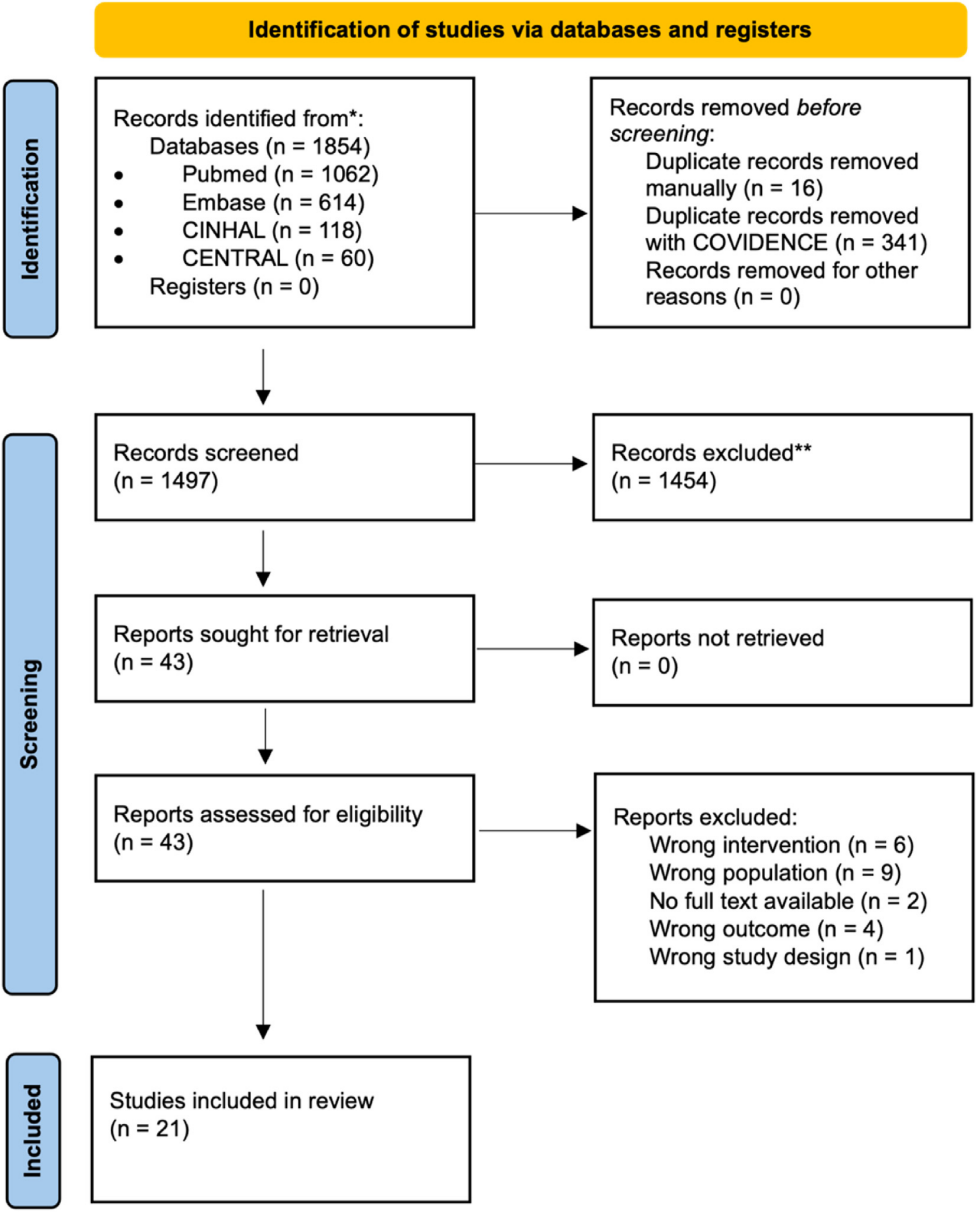
PRISMA 2020 flow diagramme.

The search across the databases yielded 1854 articles. After the removal of duplicates, 357 articles were excluded. Subsequently, the authors screened titles and abstracts, excluding 1454 articles. The full texts of the remaining 43 articles were then read. The third author SB was consulted for two cases during the title and abstract screening, as the other authors were blind to each other's decision and could not consult one another. SB helped resolve these conflicts. In all the other cases, consensus was reached before escalating to the third author. As shown in the PRISMA flow diagram (Fig. 1), the final number of articles included was 21. The reasons for excluding the remaining 19 articles were 6 for wrong intervention, 9 for examining a wrong population, 4 for wrong outcome, 1 for wrong study design, and the full text was unavailable for two articles. Detailed analysis of the excluded full texts and the specific reasons for their exclusion can be found in Supplementary Material 2.

### 3.2 Study characteristics

The 21 included studies comprised 458 participants, ranging approximately from 35 to 89 years of age. The studies were published between 2001 and 2024 and were conducted in Australia (8) [[35], [36], [37], [38], [39], [40], [41], [42]], the Netherlands [43], the USA (2) [22,44], Sweden [45], Canada (2) [46,47], Iceland [21], Italy [48], Jordan [49], New Zealand [50], Greenland [51], and the United Kingdom (2) [52,53]. The main characteristics (title, country, study design and analysis, sampling strategy, population, intervention, and clinical characteristics) of the included articles are presented in Table 1. Supplementary Material 3 displays the various themes, sub-themes and quotes extracted by the authors from the studies.

**Table 1:** Summary of the findings.

References	Country	Study design and analysis	Sampling strategy & Diagnostic criteria	Population	Outcome measures	Intervention
**Hinman et al. (2023)**	Australia	Semi structured telephone individual interviews after completing the intervention Reflexive thematic analysis within each subgroup using grounded theory principles	Purposive sampling National Institute for Health and Care Excellence Criteria	**Number**: 26 **Age**, range: 45–84 years **Sex**, N (%): • Male: 9 (35) • Female: 17 (65) **Ethnicity/Citizenship**, N (%): N.A. **Weight,** N (%): • Severely obese: 1 (4) • Obese: 9 (35) • Overweight: 11 (42) • Healthy: 5 (19) **Pathology**: Unspecified OA	Ν.Α.	**Type:** Exercise and physical activity programme **Method:** 5 individual consultations over 3 months (Physical therapists prescribed an individualised strengthening program (5–6 home exercises, 3 times/week))
**Veenhof et al. (2006)**	Netherlands	Open-ended interviews performed at the participants' home Grounded theory principles	Model of deliberate sampling for heterogeneity American College of Rheumatology Criteria	**Number**: 12 **Age,** range: 51–80 years **Gender**, N (%): • Male: 4 (33) • Female: 8 (67) **Ethnicity/Citizenship**, N (%): N.A. **Weight,** N (%): N.A. **Pathology**: • Hip: 2 (17) • Knee: 9 (75) • Hip and knee: 1 (8)	• VAS Pain (0–10), range: 3,4–5,8 • WOMAC Stiffness (0–10), mean: 3.9 • WOMAC physical function (0–100), range: 31.5–32.5 • 5-min walking test, range: 4.5–5	**Type:** Behavioural graded activity **Method:** A maximum of 18 sessions were delivered over a 12-week period, followed by five pre-set booster sessions in week 18
**Wallis et al. (2020)**	Australia	Semi-structured interviews performed after the intervention Inductive thematic analysis	Purposive sampling National Institute for Health and Care Excellence Criteria	**Number**: 20 **Age, mean:** 70 years **Sex/Gender**^a^, N (%): • Male: 6 (30) • Female: 14 (70) **Ethnicity/Citizenship**, N (%): N.A. **Weight,** N (%): N.A. **Pathology**: • Hip: 3 (15) • Knee: 7 (35) • Hip and knee: 10 (50)	N.A.	**Type:** Education and exercise program **Method:** 2 patient education sessions related to OA self-management, plus 12 sessions of joint-specific neuromuscular exercise with physiotherapist supervision
**Lawford et al. (2022)**	Australia	Semi-structured individual telephone interviews after trial completion Inductive thematic analysis	Purposive sampling Radiographic and Clinical OA Diagnosis	**Number**: 22 **Age**, mean (range): 67 years (52–79) **Sex**, N (%): • Male: 9 (41) • Female: 13 (59) **Ethnicity/Citizenship**, N (%): N.A. **Weight:** All obese (BMI >30) **Pathology**: Unspecified OA	N.A.	**Type:** Exercise program **Method:** Five individual physiotherapy sessions over 12 weeks and home exercise program
**Griesemer et al. (2020)**	USA (North Carolina)	Telephone semi structured interviews after trial completion Thematic Narrative methodology	Purposive sampling Self-reported OA Diagnosis	**Number**: 19 **Age,** mean: 61,9 years **Sex/Gender**^a^, N (%): • Male: 7 (37) • Female: 12 (63) **Ethnicity/Citizenship**, N (%): • Non-Hispanic White: 15 (79) • Black: 4 (21) **Weight:** N.A. **Pathology**: Unspecified OA	N.A.	**Type:** Exercise and physical activity program **Method:** They attended a 3-h Active Living Class. Every participants had to do less than 120 ​min of moderate-to-vigorous PA (MVPA) per week
**Ledingham et al. (2020)**	USA	Qualitative semi-structured interviews after trail completion Grounded theory	Purposive sampling Self-reported Doctor-Diagnosed OA	**Number**: 25 **Age**, mean (range); 67 years (57–79) **Sex**, N (%): • Male: 4 (16) • Female: 21 (84) **Ethnicity/Citizenship**, N (%): • White: 12 (48) • Black: 9 (36) • Other 4 (16) **Weight:** N.A. **Pathology**: All knee OA	• WOMAC pain (0–500), mean: 205 • WOMAC physical function (0–1700 ​mm), mean: 725	**Type:** Exercise program **Method:** 6-week strength-training exercise class and exercises at home once weekly
**Östlind et al. (2022)**	Sweden	Three focus groups Content analysis	Combination of purposive and convenience sampling Self-reported OA or Clinical Diagnosis	**Number**: 18 **Age**, mean: 58 years **Sex**, N (%): • Male: 5 (28) • Female: 13 (72) **Ethnicity/Citizenship**: N.A. **Weight:** N.A. **Pathology**: Unspecified OA	N.A.	**Type:** Education and exercise program **Method:** Participants received information about OA, self-management, and exercise in group lectures
**Leese et al. (2021)**	Canada	Qualitative semi-structured interviews after trial completion Phenomenographic analytical methods	Purposive Sampling Clinical OA Diagnosis	**Number**: 21 **Age**, mean (range): 58 years (40–82) **Sex**, N (%): • Male: 9V2 (57) **Ethnicity**: N.A. **Weight:** N.A. **Pathology**: All knee OA	N.A.	**Type:** Physical activity program **Method:** Participants received a 1.5-h session of education, a wearable device and an individual counseling. 8-week intervention
**Nelligan et al. (2020)**	Australia	Phone individual semi-structured interviews within two months of completing the intervention Inductive thematic analysis by Braun & Clarke	Purposive sampling National Institute for Health and Care Excellence Criteria	**Number**: 16 **Age**, mean (range): 63 (48–73) **Sex,** N (%): • Male: 8 (50) • Female: 8 (50) **Ethnicity/Citizenship**: Australian **Pathology**: All knee OA	• NRS, Pain (range): 4–8	**Type**: Exercise programme **Method**: Website and 24-week mobile phone SMS
**Petursdottir et al. (2010)**	Iceland	Interview-based study after or during the intervention Vancouver School of Doing Phenomenology	Purposeful sample Radiographic OA Diagnosis	**Number**: 12 **Age**, mean (range): 67 (50–81) **Sex**, N (%): • Male: 3 (25) • Female: 9 (75) **Ethnicity/Citizenship**: Icelanders **Pathology**: Lower Limb O	N.A.	**Type**: Exercise programme **Method**: In exercise facilities
**Battista al. (2021)**	Italy	Semi-structured interview study after or during the intervention Descriptive phenomenological approach – theme-based analysis	Purposeful sample and snowball sampling Clinical Physician OA Diagnosis	**Number**: 11 **Age**, mean (range): 61 (45–73) **Gender**, N (%): • Male: 5 (45) • Female: 6 (55) **Ethnicity/Citizenship**: Italian **Pathology**: OA • Hip: 5 (45) • Knee: 3 (27) • Hip and knee: 3 (27)	N.A.	**Type**: Exercise programme in Osteoarthritis care **Method**: Italian National Healthcare System
**Stone and Baker (2015)**	Canada	Semi-structured interview study after the intervention Interpretational Analysis & Deductive Thematic Constructionist Approach	Purposeful, voluntary and snowball sampling Clinical Physician OA Diagnosis	**Number**: 15 **Age**, mean (range): 54 (30–85) **Sex**, N (%): • Male: 6 (40) • Female: 9 (60) **Ethnicity/Citizenship**: Canadian **Pathology**: Hip: 2 (13) • Knee: 11 (73) • Hip and knee: 2 (13)	• Geriatric Depression Scale Short Form (range): 4–12	**Type**: Physician-recommended treatments, including physical activity **Method**: Not specified
**Al-Khlaifat et al. (2020)**	Jordan	Triangulation methods study adopting focus group and structured interviews after the intervention Ontological approach & framework analysis	Convenient sampling Clinical and Radiographic OA Diagnosis	**Number**: 14 **Age**, mean (range): 60 (43–77) **Gender**, N (%): • Male: 1 (7) • Female: 13 (93) **Ethnicity/Citizenship**: Arabians **Pathology**: Knee OA	• Pain (VAS, mean): 5.43	**Type**: Exercise programme **Method**: Not specified
**Moody et al. (2012)**	New Zealand	Semi-structured focus group study after the intervention The General Inductive Approach (Thomas 2006)	Convenient sampling Self-reported OA Diagnosis	**Number**: 17 **Age**, mean (range): 78 (68–89) **Sex/Gender**^a^, N (%): • Male: 4 (24) • Female: 13 (76) **Ethnicity/Citizenship**: New Zealander **Pathology**: Lower limb OA	N.A.	**Type**: Twelve-week aqua-aerobics programme **Method**: In swimming pools
**Hinman et al. (2016)**	Australia	Cross-sectional qualitative study drawing from symbolic interactionism semi-structured individual interviews after the intervention Grounded Theory	Convenient sampling American College of Rheumatology Criteria	**Number**: 6 **Age**, mean (range): 62 (50–69) **Sex**, N (%): • Male: 3 (50) • Female: 3 (50) **Ethnicity/Citizenship**: Australian **Pathology**: Knee OA	• Pain WOMAC (range): 4–7 • Physical Function WOMAC (range): 13–34 • Quality of Life (range): 0.4–0.9 • Distress^b^ (range): 2–20	**Type**: Delivery of a structured exercise and physical activity programme **Method**: Structured, informal booklet and telephone coach
**Moore et al. (2020)**	United Kingdom	Semi-structured interview study post-intervention and follow-up Layered approach to thematic Analysis	Purposive sampling National Institute for Health and Care Excellence Criteria	**Number**: 22 **Age**, mean (range): N.A. **Gender**, N (%): • Male: N.A. • Female: N.A. **Ethnicity/Citizenship**: N.A. **Pathology**: Knee OA	N.A.	**Type**: Usual physiotherapy care, individually tailored exercise, and targeted exercise adherence **Method**: Not specified.
**Campbell et al. (2001)**	United Kingdom	Semi-structured interview-based qualitative study after the intervention Thematic Analysis	Convenient sampling Clinical Physician OA Diagnosis	**Number**: 20 **Age**, mean (range): N.A. **Sex** N (%): • Male: 6 (20) • Female: 14 (70) **Ethnicity/Citizenship**: British **Pathology**: Knee OA	N.A.	**Type**: Exercise programme **Method**: Home based
**Bell et al. (2024)**	Australia	Semi-structured interview study post intervention Reflexive thematic analysis	Convenient Sampling National Institute for Health and Care Excellence Criteria	**Number**: 19 **Age**, mean (range): 62 (55–69) **Sex** N (%): • Male: 7 (37) • Female: 12 (63) **Ethnicity/Citizenship**: Australian **Pathology**: Knee OA	• KOOS (range): 20–64	**Type:** The Good Life with osteoArthritis from Denmark (GLA:D) **Method:** Structured, in person or via telehealth
**Lawford et al. (2024)**	Australia	Online short-online survey at the of their treatment Content and Reflexive Thematic Analysis	Convenient Sampling National Institute for Health and Care Excellence Criteria	**Number**: 122 **Age**, mean (range): 61 (53–69) **Sex** N (%): • Male: 33 (27) • Female: 89 (73) **Ethnicity/Citizenship**: Australian **Pathology**: Knee OA	• Knee pain NRS (mean): 6 • WOMAN (mean): 20	**Type:** Telerehabilitation exercise programme with a physiotherapist **Method:** Structured via zoom
**Allison et al. (2023)**	Australia	Semi-structured interview study post intervention Qualitative inductive thematic analysis	Purposive sampling National Institute for Health and Care Excellence Criteria	**Number**: 13 **Age**, mean: 61 **Sex** N (%): • Male: 4 (30) • Female: 9 (70) **Ethnicity/Citizens**: Australian **Pathology**: Hip OA	• Hip pain (NRS, mean): 5 • WOMAC, function score (mean): 15	**Type:** Aerobic physical activity exercise intervention **Method:** Home exercise programme
**Nielsen et al. (2024)**	Greenland	Semi-structured interview study during and post intervention Interpretive description approach	Purposive sampling Clinical Physician OA Diagnosis	**Number**: 10 **Age**, mean (range): 58 (46–79) **Sex** N (%): • Male: 4 (40) • Female: 6 (6) **Ethnicity/Citizenship**: Eight were Inuit born in Greenland, two were Caucasian born in Denmark **Pathology**: Hip or knee OA	N.A.	**Type:** Self-management education and exercise intervention **Method:** Education and supervised group-based exercise in person

Legend: N, number; N.A., not available.

a it was not specified if it was the sex assigned at birth or the gender; VAS, Visual Analogue Scale; WOMAC, Western Ontario and McMaster Universities Osteoarthritis Index; OA, osteoarthritis; BMI, Body Mass Index; NRS, Numeric Rating Scale; KOOS; Knee Injury and Osteoarthritis Outcome Score; a, Assessment of Quality of Life instrument version 2; b, Depression, Anxiety and Stress scale.

### 3.3 Appraisal results

Table 2 presents the results of the CASP assessments for each article, including responses and corresponding comments. The included studies generally had good methodological quality. However, one of the most overlooked items was the relationship between researchers and participants, as some authors did not critically examine their role, potential bias, and influence during the formulation of the research questions and data collection. In all cases, the research aims were clearly stated, and the qualitative methodology was appropriate.

**Table 2:** Quality assessment of the included studies (CASP).

	Item 1	Item 2	Item 3	Item 4	Item 5	Item 6	Item 7	Item 8	Item 9	Item 10
**Nelligan et al. (2020)**	Y	Y	CT (The researchers did not justify and report why they chose this study design)	Y	Y	CT (No explanation on the relationship with the participants)	Y	Y	Y	Yes. The study is useful and gives suggestions for the use of eHealth programs in OA. It identified new areas where research is necessary such as how health professionals might take an active role in dissemination.
**Petursdottir et al. (2010)**	Y	Y	Y	Y	Y	Y	Y	Y	Y	Yes, the study shed some light on the perceived barriers and facilitators to exercise in people with OA, identifying new areas of interests. They also discussed the transferability of the findings to a wider population.
**Battista et al. (2021)**	Y	Y	Y	Y	Y	Y	Y	Y	Y	Yes, the study clearly and significantly investigates the care process experience of people with OA, critically analysing the transferability of the results in other Italian and European regions. Moreover, they highlighted how it is necessary to explore deeply the whole care process of people with OA.
**Stone et al. (2015)**	Y	Y	CT (The researchers did not justify and report why they chose this study design)	N (Convenient sampling)	Y	CT (This detail is never addressed anywhere in the article)	Y	CT (the data analysis is not explained in detail).	Y	Yes, this study investigated the facilitators and barriers to active lifestyle, including physical activity, in OA, but it is not quite explicit how the analysis was conducted. The authors also discussed the future implications and directions. The transferability of results has been discussed
**Al-Khlaifat et al. (2020)**	Y	Y	Y	N (convenient sampling)	Y	Y	Y	CT (it was rigorous but for the last theme there are no quotes and there are no table or figure in regard of the generated themes)	Y	Yes, overall, the study is interesting and shed some light on the exercise experience in people with OA. New areas of research are discussed, as well as the transferability of results.
**Moody et al. (2012)**	Y	Y	CT (no specific declaration on this topic)	N (Convenient Sampling)	Y	N (It is just declared that the same researcher conducted all focus groups).	Y	CT (the quotations are sometimes small, and they do not refer to a single person but to the entire group. Moreover, there is no table of descriptive characteristics of sample).	Y	Yes, the study is overall interesting but ha some concerns related to the methodology robustness. The transferability has been discussed emphasising the limits and strength of the study, but there is no section on discussion on future possible studies.
**Hinman et al. (2016)**	Y	Y	Y	N (Convenient sampling)	Y	CT (it is declared just this “the same investigator conducted (mostly over the telephone) and transcribed all interviews)	Y	Y	Y	Yes, the study is interesting and is methodologically rigorous, discussing the generalisability and possible future implications, while addressing relevant issues related to adherence to treatments.
**Moore et al. (2020)**	Y	Y	Y	N (Convenient sampling).	Y	Y	Y	Y	Y	Yes, the study presents interesting results, but it is not clear who are the participants who took part in the interviews. Moreover, future research implications are well described.
**Campbell et al., (2001)**	Y	Y	Y	N (Convenient sampling).	Y	Y	CT (not possible to find references about ethical issues' consideration).	Y	Y	Yes, the study has some minor lacks in methodological rigorousness, but it is overall interesting, even though transferability of results is not considered as well as future studies implications.
**Griesemer et al. (2020)**	Y	Y	Y	Y	N (No topic guide available No discussion about data saturation)	CT (The first author conducted all of the interviews by phone, and there are not statement of any potential conflict of interest).	Y	N (It is not clear how the categories/themes were derived from the data. The researcher did not explain how the presented data were selected from the original sample to demonstrate the analysis process). Only few quotations are reported).	Y	This research likely contributes to the understanding of couple dynamics related to physical activity, which may be an underexplored area in the existing literature. By qualitatively studying how couples engage with physical activity together, the study provides insights into relationship dynamics, motivations, and barriers that influence participation in physical activity.
**Hinman et al. (2023)**	Y	Y	Y	CT (No explanation of the sampling method)	Y	Y	Y	Y	Y	The research considers their findings in relation to current practice or policy and relevant research-based literature. By exploring factors influencing treatment response in knee OA patients undergoing exercise interventions, they provide valuable insights that can inform clinical decision-making.
**Lawford et al. (2022)**	Y	Y	Y	CT (No explanation of the sampling method)	Y	Y	Y	Y	Y	The research likely contributes to existing knowledge by exploring the challenges faced by individuals with knee OA and comorbid obesity when engaging in strengthening exercises. By qualitatively examining the perspectives of both patients and physical therapists, the study highlighted previously understudied barriers and facilitators to exercise adherence in this population. The research article has the potential to contribute valuable insights to the fields of physiotherapy, OA management, and obesity treatment, by addressing important clinical and research gaps related to strengthening exercises in individuals with knee OA and comorbid obesity
**Ledingham et al. (2020)**	Y	Y	Y	Y	Y	Y	Y	Y	Y	The research added value to the field by providing insights into exercise adherence beliefs among adults with knee OA over an extended period, and by identifying potential areas for further research and intervention development in this area.
**Leese et al. (2021)**	Y	Y	Y	CT (No explanation of the sampling method)	Y	CT (It is not described the relationship between researcher and participants)	Y	Y	Y	The study contributes to the understanding of how wearable technology is experienced by individuals with knee osteo-arthritis who are participating in a physical activity counseling intervention. By qualitatively exploring participants' experiences through a relational ethics lens, the research offered valuable insights into the ethical dimensions of using wearable technology in healthcare interventions, as well as its impact on participants' relationships with their own bodies, healthcare providers, and caregivers. The article discussed its findings in relation to current practice guidelines for OA management.
**Ostlind et al. (2022)**	Y	Y	Y	CT (In this study, a combination of purposive and convenience sampling methods was used)	Y	Y	Y	Y	Y	The study contributes to the understanding of how activity monitoring and digital support are perceived among working individuals with hip and knee OA. By qualitatively exploring participants' experiences and perceptions through focus group discussions, the research offered valuable insights into the role of digital tools in supporting self-management and work-related activities in this population. The article discussed its findings in relation to current practice guidelines for OA management, policies, and existing research-based literature on digital health interventions and chronic disease management.
**Veehnof et al. (2006)**	Y	Y	CT (However, they did not discuss which method they used).	CT (We do not know who decided to recruit the participants and if there were any conflicts of interest).	Y	CT (Researcher do not critically examined their own role in sample recruitment and choice of location).	Y	CT (The authors reported a small number of quotations).	Y	The researchers likely discussed their findings in relation to current practice or policy and relevant research-based literature. By exploring factors influencing long-term adherence to behavioural graded activity in people with OA, they contribute to understanding the complexities of OA management and rehabilitation.
**Wallis et al. (2020)**	Y	Y	CT (They did not discuss the method they adopted).	CT (No explanation on the purposeful sampling method).	Y	Y	Y	Y	Y	The researchers likely discussed their findings in relation to current practice, policy, and relevant research-based literature. By exploring barriers and enablers to uptake of a contemporary guideline-based management program for hip and knee OA, they contributed to understanding the challenges and facilitators in implementing evidence-based care for this condition.
**Bell et al. (2024)**	Y	Y	Y	N (Convenient sampling).	Y	Y	Y	Y	Y	The researchers discussed the findings of their study highlighting their role in physical activity participation related to exercise-therapy. Their results matched with quantitative explored the complexities of barriers and facilitators to physical activity participation following GLA:D.
**Lawford et al. (2024)**	Y	Y	Y	N (Convenient sampling).	N (No disclosure about the choice of using an online survey).	CT (Researcher do not examined their own role).	Y	Y	Y	The study emphasises the experience and satisfaction g telerehabilitation for people with knee OA. The findings highlight the role of the expectations of people attending these programmes.
**Allison et al. (2023)**	Y	Y	Y	CT (No explanation on the purposeful sampling method).	Y	Y	Y	Y	Y	The researchers likely discussed their findings about the acceptability, facilitators and barriers to engagement with the exercise programme in people with hip OA, exploring also the perspectives of physiotherapists.
**Nielsen et al. (2024)**	Y	Y	Y	Y	Y	Y	Y	Y	Y	The study contributes to expanding the findings related to the experience of people with knee or hip OA attending a structured exercise and education interventions for their condition. Specifically, they highlighted the role of social and organisational factors influencing the acceptability of exercise programme.

Legend: Y, Yes; CT, Can't tell; N, No; OA, Osteoarthritis; GLA:D, Good Life with osteoArthritis from Denmark.

### 3.4 Results of the synthesis

Seven descriptive themes were created from the thematic analysis summarising the results of the primary studies (The Role of Beliefs, The experience of the exercise program, Individuals' mindset, Relationship with the health professional, Social aspects, Environmental Circumstances, Technological support). These descriptive themes were further interpreted, creating three analytical themes ((i) Mind-Body Connection, (ii) Social Support Systems, and (iii) Environmental Enablers) to understand the perceived barriers and facilitators to exercise adherence in OA. The codes and a summary of quotations that led to the generation of these themes are listed in Table 3. The complete list of quotations is reported inSupplementary Material 4.

**Table 3:** Analytical and descriptive themes with codes and quotations.

Analytical Themes	Descriptive Themes	Codes	Quotes
**Mind-Body Connection**	The experience of the exercise programme	Doing the exercise following clear instructions, handbooks, website or wearable devices	“Now I think I handle it more wisely. I know better because I've been fortunate to get good instruction” [21] “I think, for me, it was 2 separate. The physio [physical therapist] was concentrating and getting me better virtually, and there's she (the coach) asking how I am managing, and, in a way, yet it goes hand in glove.” [36] “Reassure me. Reassure me. Give me the right exercises to do, if it wasn't going to do any further damage, if it was arthritis. I think the cartilage problem is caused by, or could have been caused by the arthritis. I needed reassurance that it was okay to actually do the exercises and I wasn't going to cause further damage. Confidence I think because sometimes trying to do these things on your own is a bit scary if you get stuck, because my leg does lock. And I think probably him encouraging me to do the right exercises, and do them every day, which I did do.” [53]
		Knowing exactly what to do	“It got me where I had a wide variety of different exercises that I could do and I felt supported and I knew what I needed to do, so I didn't really need more [physio consults].” [35]
		Personalised exercises and progression	“And I think that it is important when people choose which exercises to do, that you enjoy it, that you feel it is rewarding … these positive factors have to be present” [21] “I thought, for me, it was beneficial to have both. Initially, I thought no, one's enough, I don't need this health coach, but I think putting the 2 together, I think it was beneficial, and it was good. They sort of complemented each other in different ways. Yeah, like I said, one was a pure business-type person, and the other was a very personal person, so they did complement each other, and it worked for me.” [36] “Overall, I think, if you have the right patients, the WBE one is fine and the [NWBE] one might be the person learning the exercises and there isn't that heavy grinding or clunking feeling when they loaded.” [37]
		Improving your confidence	“… it's helped me not only with my mobility but my self-confidence to be able to go, yeah, I can get up there all right and come down there.” [41] “But we covered the book and it got me to a stage where I was comfortable doing my exercise and there was nothing of concern really …” [35] “And it seems like everything I did here [during the exercise class], I was able to do at home with no problems.” [22]
		Feeling stronger and more flexible	“And so my aim was to … get back to closer to 15 to 20,000 steps a day – which I achieved. So doing the exercises and strengthening the knee, I was able to get back to all of that again … Realistically I knew it was not going to get me back to running.” [35] “I felt so much stronger. I could barely walk, I'd use a walker – inside the house, just to go to the sink. And when I first went there I could barely walk and I was doing just a few hundred steps a day … And then I worked up to 6000 and – I could walk to the sink without my walker. So I definitely got improvement as far as strength went.” [35] “It's quite what I needed, and also I found that I wasn't able to … I wasn't able to vacuum and mop the floors in the same—timeframe, so it's a case of mop or vacuum 1 day and not another day. But now I'm back to being able to do it all in one go and that starts the side movement as well.” [39]
		Learning to self-manage and pace	“I still do [the exercises] and I remember to stand the correct way without even thinking about it now… [The pain] has been a lot better, much better, and I can do things better. Dressing—I don't have to hold on to anything, I can balance now and in fact, you know, I find it a great improvement.” [52] “We had it for so long that I felt that at 8000 [steps] it started to get too tough afterwards, so I tried to stick to it, and I thought it worked well. Ten there was never any [pain] … So previously I activated myself a lot and then nothing … It became a much better rhythm.” [45] “I've learnt a lot about my body and what I can do. I've learnt to watch for trigger signs and what do I do when that happens. So all in all, it has been a positive experience, but a lot of having to adjust to what I felt was working for me.” [39]
		Enjoying exercising	“It's part of life, it's what I do. I Get up in the morning, I have a cup of coffee, I take my blood pressure medication, then I go and do my exercises, and then I come back and have breakfast, and that's just become a routine, which in a way is no different from people going to the gym 3 times a week or doing anything else, so it's my way of doing things. And I don't have to leave home!” [36] “We were talking about gardening whatever as I was doing my exercises as well. And she sort of mentioned things about her life and what she could do with various bits and pieces and it just made it a much more enjoyable experience I think. [.] I think it made a difference. It made me feel I wanted to do the exercises more.” [53]
		Getting positive feedback after doing the exercise	“It's a very good thing if you've got their GP onside. So, perhaps it'd be good idea to have some group sessions with the GPs. By group sessions, I don't mean just getting them in a room like this and talking to them. I Mean tell them to come along in their exercise togs and actually do it.” [37] It was very often that I looked at [the WAT] and … Oh, ok, so now I have cycled for 20 min at a high pace, and I received no credit for it. It's annoying.” [45]
		Feeling self-blamed for not exercising	“I really just think it was – I really think it was because I wasn't doing what I needed to do. I wasn't – it was mainly in the walking and things like that, I just wasn't doing it.” [35] Joseph: “I think for me it's more disappointment for not following it through like I should have followed it through I guess … At the end of the day when I did turn up it was really good.” [35] Geoffrey: “as I told him [one of the doctors running the trial] really I feel a bit guilty taking his time up because there must be a lot of people a lot worse than what I am.” [52]
		Comorbidities affecting adherence to exercise	“I get depression, so sometimes I just fall into a big hole and can't quite function very well. So, we just got through that.” [37] “I was really sick for quite a long time and I ended up with pneumonia.” [37] “… The inflammatory conditions that I've had with hyperthyroid, the whole package of things that had slowed me down tremendously over the last three or four years … hopefully if my hip settles down … That's the limiting factor with that now, not my knees …” [42]
		Fatigue	“The [WBE] group would find those exercises more of a mental - mentally tiring. Focusing, concentrating, than actually getting an actual muscular exertion sense … for them it was not about the load on their muscles necessarily, it was about how much cognitive effort it was. Mental effort, for them to do the right alignment.” [37] “Well for me, at first that's why I missed some of them. I couldn't go more than one because I was just so tired the next day and would sleep so sound, you know at the night-time, that I couldn't always wake up early enough to get myself organised to get the bus.” [50] ‘I suppose maybe I felt a bit overwhelmed by the combination of strength and aerobic exercise—and that's why I couldn't commit as much to the strengthening as I wanted to—and that's why you come up with all these reasons why you can't do it. So perhaps that was a little bit overwhelming.’ [39]
		Complexity of exercises	“The way that I had to attach the weights to my leg, it was just about impossible to do it by myself … if they were a lot easier to use I probably would've kept them up a bit more than what I did, but it was just very awkward.” [37] “it's pretty difficult to manoeuvre when it's not properly strapped in around your ankle … that was probably the hardest part on me, was preparation … It was the reconfiguration of the equipment that weighed on my mind before I said, “Oh, gee, I've got to go do that again. I'm going to blow about an hour.” [37] “The only one I had trouble with was the lightweight with the straight leg, lifting that up. I had a little bit of trouble - It was something to do with my lower back, where your spine goes down and separates towards your buttock.” [37]
		Stop exercising once feeling better	“I followed exactly what I had to do. Yes, 100%.” [35] “Since you have stopped seeing [the physiotherapist] have you stopped doing the exercises?” Geoffrey: “Yes I'm sorry I have yes. But as I said I haven't had no pain. … I wondered whether it was temperature or dampness or something like that you see. Now there is nothing wrong with them.” MT: “So you feel if there is nothing wrong with it you feel there is not much point in a …” Geoffrey: “Well that's it. It's the wrong attitude I know.” [52] “I wanted to get rid of the pain. If the pain disappears, why would I bother to continue the exercises? I Understand it is better to do the exercises to avoid the pain returning, but, if the pain returns, I will start the exercises again.” [43]
		Losing the interest in the long run	“The first time I went back it was I pretty much did them all. And then the second time I went back I did, I don't know, three-quarters. And the third time I went back I did half and sort of dwindled away so by the fifth time I went back it was hardly anything.” [35] “At end of treatment was partially adherent to exercises and was an active hill walker. At follow-up no longer did the exercises from the trial but was an active hill walker and had joined a gym.” [53] “At end of treatment was adherent to exercises, used exercise bike. At follow-up was not adherent to exercises from the trial but did other sitting exercises, cycled and walked.” [53]
		Previous negative experiences	“I played sports when I was young, but then I quit. I never had any endurance, so I was never good at it.” [21] “I felt like I wanted to do it but couldn't. So I felt limited by my body rather than attitude. I felt a high degree of frustration with my inability to do things that I wanted to do.” [42] ‘I just think if you haven't been used to doing that, I think that's with a lot of things in life, if it's something you've done all your life, if you've played cricket you'll play it in your 50s, but if you just try and take it up in your 50s … It won't work, it'll do your hammie or something. But, you know, it's like a lot of things, if that's what you're used to doing … if your body is conditioned for that you aren't so cautious.’ [39]
		Difficulty in starting the exercises	“I think it's a problem, that you can't get in … Tat I can't make it work. I feel it's like a sort of handicap. But once it works, it's amazing.” [45] “It is good for our age if we learned how to swim but it is hard to learn from our children and we are afraid of water.” [49]
		Feeling too much pain	“So it is that maybe when you are old, people back down, they lie on the couch … Surely such a pain affecting someone who does not have that drive [motivation to stay fit] makes people unwilling to get up from the couch” [48] “I can't bend down. I can't get on the floor, if I do, it is a chore for me to get up. Bending my knees hurts all the time. Walking now seems to be hurting me as well” [47] “If someone called to play ball or something I would say, “I'm busy, I can't,” and pretty soon I realised that I couldn't do it, not that I didn't want to, I just couldn't anymore. It wasn't worth the pain” [47]
		Seeing/not seeing the benefits of continuing to exercise	“The improvement it made … I suppose [I was surprised] that the type of exercise I did could make a difference, I wouldn't have thought the exercises I was doing would make any difference at all this study taught me that exercise can definitely help with mobility, with arthritis and I think a lot of people including myself were frightened of that, thinking “oh no, I'm going to hurt myself. Going to injure myself. Going to wear out my knees”, you know – it was the opposite effect. The more you move, the better it feels, you know?” [37] “He was physically incapable of sitting up and within four weeks of that program, particularly the floor exercise, he can get up and he sits. It was just amazing.” [37] “It [physical exercise] is not like taking supplements with hyaluronic acid, those (supplements) you do not see what they do.” [48]
		Personal preferences	“I hate exercise. I have to say, I hate it. I'm one of these people that never go to the gym for exercise.” [37] “Well, there was this note on the wall saying the aqua-exercise classes are about to start … But for whom?” [37] “It was boring. Every day, every other day, when you do the same thing, it's very hard to get motivated, it was a bit boring. Some of the exercises were OK, but some of the exercises … just thought of throwing in the towel virtually, but then I thought the pain versus this, and then it will balance everything out.” [36] “If perhaps my wife would work with me and you had a bit of competition, but I feel such a fool standing on one leg and going up and down on my own and I tends to drop it I do. I'm not very strong disciplined on that, no. I know some people can be so, but not me. I suppose if there was a really good reason I would.” [52]
	Individuals' mindset (way of thinking about things)	Recognising that you need to find the time	“It's not so much the time. I think everyone's got time. It's just whether mentally you can get yourself in a frame of actually doing it. That's the issue. I think we all got time. You're kidding yourself if you didn't.” [37] “It's just excuses when it comes down to basics. I mean you know you could get up in the morning and do it between 6 or 7 or something like that.” [52]
		Importance of giving a positive narration about an exercise programme	“I think reinforcement of the benefit [is important] because I think they probably have enough information now to say, well, if you do stick to it, if you do it the way you're supposed to do it, the number of times a week you're supposed to do it, you will see an improvement. But you can't, if you go at it half-heartedly then you get a half-hearted result.” [37] “I think putting a positive spin on it saying that you're going to improve, so if take part in this program, you're going to feel better, you're going to improve, you're going to walk back, so I think something positive.” [37]
		Being positive (mindset) to keep living with OA	“My general health is pretty good. I don't have any major issues.” [35] “I think that general positivism is part of your health; if you think constantly about pain and aches, then you get really sick” [21] One becomes sad because it limits one's quality of life, but when one receives such help with exercise, one becomes a bit more positive again. I am naturally positive-minded, so I try to see the good in things and make the best of it [51].
		Strong motivation	“You can go too far with this, as you said, you push yourself and then you have to do a little more and then you have to do a little more and you will never be satisfied.” [45] “Well I thought it was marvellous really it um you know got us out of bed in the morning and got us into the pool and umm the instructor we had was very, very good and ah I think it was just so good. And I think the motivation was there which is the big thing is to get you motivated you know?” [50] “I found it a little bit intimidating at first [the exercise class], ‘cause it was like an obstacle course, where you had to do this, then you went from that to a [another] thing … I'm saying, “Oh gosh, I can't do this. This is a bit much.” But then I said, “But no. Let me give it a shot and just try to do it,” and it's not like I gotta be vigorous with it, just take my time, go at it.” [22]
		No more interest in taking drugs	“I've really gone off painkillers, so I rarely take an ibuprofen now and I rarely take anything stronger.” [37] “I'm a great believer in physiotherapy anyway I think. I don't agree with drugs quite as much as, I think, if you can have it naturally.” [52]
		Accommodation is part of OA to keep living your life	“It's just there. It's just part of me now. So, I don't feel necessarily … oh, I guess, what I feel down about is when I'm with other people and they go for a big long walk, and I say, “I have to sit down, I'll wait for you here.” So, that's pretty annoying, but other than that, I just have to accommodate it into my life.” [35] “But now I've decided to quit driving.” [21]
		Lack of commitment	“I reckon – quite frankly, I reckon there's a 50/50 chance. And I couldn't commit to anything further than that, because as I said, work is of the utmost – unless, as I said before – unless there's a way in which I can go on sick leave.” [37] “I think so, for laziness. Because if you want to, you are able to find the time. So it is, therefore, laziness” [48] “Virtually I guess what I'm saying, the problem was probably 100 % my lack of 100 % commitment rather than any fault of the study.” [35]
	The role of Beliefs (strong feeling that something is true or real)	Knowing the mechanisms of pain and OA	“Just being told that it’s okay to feel pain … I think it was finding that it needed to actually be uncomfortable, nobody had ever said that, they just said you need exercises and I'd sort of slightly cheat, I'd sort of do them but not really do them because I didn't know what it was meant to feel like” [37] “When I was first diagnosed, I didn't know what to think. I knew it wasn't good, but I didn't know how bad it was going to be. After a couple of years, the pain was too much to bear and I thought, that's it … my life is over. And no one warned me … I didn't even know what to do … exercise was the farthest thing from my mind.” [47] “With the knee, I suppose I was imagining there'd be more a hands-on assessment of my knee, and it was just really ‘How are you going with the exercises?’ ‘Are you doing them?’ ‘Aren't you, and how can you do it so it's not as painful?’” [36]
		Exercise will reduce pain after/before surgery or not	“The knowledge that exercise can help. I had no idea that actually exercise could help like that … that knowledge is the big one that really surprised me” [37] “I continue with my exercises, they are integrated in my daily living. I really know these exercises have beneficial effects and that motivates me to continue with my exercises. The main motivation to do all this is to prevent an operation to get a new hip” [43] “Well, I – the thing with surgery, it seems to me, is that once the surgery is taking place, and there's been the particular period of physiotherapy, that there's very little pain, as I have heard. Is that going to be the outcome of this sort of course?” [37]
		An active role will improve OA symptoms and prevent worsening	“I worked out new ways to cope, to keep my arthritis from getting in the way too much” [21] “… The body has to move …” [48] “I think there's nothing negative that can happen, even if it didn't get better, it's not a negative thing because you tried.” [35] “I know exercise is correct. That's obviously just to strengthen what you have got there and it does work. As far as I don't know, massage or manipulation or TENS machines, braces and that I don't know if that makes any difference but I agree with, well, just exercise in general. I know that works.” [35]
		Strengthening your muscles will improve OA symptoms	“If you don't keep your legs stronger – mine aren't strong enough. I know that, and the more strength you lose in the muscles around – that support your knees, the more limited you become in what your capabilities are and what you can do, and so you lose some. Without the strength in your legs, you lose your life. You lose a desire to go and do things.” [35] “It got worse and worse and I started falling down … Since I started strengthening these muscles it seems I don't fall over so much which is good … It's so embarrassing.” [52] “She explained even though the exercises might cause pain, as said, she sort of suggested that the, the problem amongst other things was the lack of strength in the muscle. So she said by building the muscles up that will support the knee better [yeah] in the long run.” [53]
		Knowing that pain related to exercise is not dangerous	“Well, now I understand it is very important, understanding that a little bit of pain is OK and how to deal and manage that pain and understand that some pain to do with any sort of physical activity is OK and I'm not doing any further damage …” [35] “Although I experience the same level of pain, I have learned to continue with my activities and I realise that I achieve more because of that” [43]
		Knowing that losing weight can help improve OA symptoms	“I think if I lost 20 kilos, which should be my ultimate game, maybe 30, I suspect my knees would improve out of sight. And I mean, I lost 10 kilos and actually after losing 10, my knees did feel a bit better. So if I lost another 20, I probably think they'd be a lot better.” [35] “And then I lost – I can't remember how much it was now, it was like a fair bit in 6 months – like 8 kilos or something – and I think nothing else would – well, apart from my physical fitness would have been pretty poor – so I think those 2 things. If I could have got more weight off more quickly, I reckon I might have seen more benefits, you know?” [35]
		No pain no gain/Ignoring pain to keep going	“You ignore pain, that's the thing too. So it comes and goes. You treat it, you get an antiinflammatory, you get a massage, do what you can and just keep going.” [35] “There's always some pain to have a gain [laughs]. So sometimes doing the exercises, yeah, I would find that there'd be some sort of pain … Yes, it hurts, yes, it's uncomfortable but hopefully it will keep it moving and going and whatever.” [35]
		Being confident that exercises will provide benefits	“Once you understand that the program will provide the benefit, then you'll finish it, but it's that first one or two sessions and until you're confident that doing this slightly painful – not necessarily painful, but difficult exercises –- until you're convinced of that, you won't complete it. And once people like me that have completed the program, helping the cause to tell other people and other volunteers it's – that's the way to get them through. That will give them the encouragement they need.” [37] “I cannot let the arthritis overtake you … I was not going to let the arthritis stop me.” [21] “That is, there were some, just some things [decisions in the care process] … Erm … I don't know … they were left to our intuition, to our perception but just because you understand that by acting in a certain way, maybe you will limit its progress [of OA] …” [48]
		Believing that exercise will not improve as joints are worn out	“The right knee was – worn out, wearing away on the inner side of the joint just because of the structure of my legs so that's what caused it. My knees are plain old worn out.” [35] “Not only does it hurt when you [move], but it would hurt the next day. The pain never lets you forget… and believe me, I don't. The only thing I can do is not do it again. Avoid exercise, avoid the pain” [47] “I don't know if exercises could prevent worsening of knees” [49]
		Believing that exercise will hurt joints	“I had been afraid to exercise because of the pain, and because of the study, I'm now aware that I can actually do something about it rather than just sit on the couch like I had been doing” [38] “I wasn't attempting any exercise on my legs. I wasn't even going there because I was just too worried about incurring more damage.” [42]
		Believing that OA is due to biomechanical causes	“… the bones now have become weak at the very end of the leg bones, where they would normally be cushioned on the meniscus, so they've become soft. And, yeah, it's just they're more tender, that's my understanding of it.” [35] “… there must be a link surely that things have to wear out. Just like your car wears out after a certain amount of kilometres.” [35] “The cartilage, yeah. So I've had three arthroscopies and meniscus tears but along that pathway, it was explained to me that I had very little articular cartilage in both knees, so I was expecting that it’s going to be a problem.” [37] “The doctor told me: “You know that if I did not know that these x-rays belong to you, I would think that they belong to another person who is at least 30 years older than you” … but, I guess I did not feel as bad as he was describing me.” [48]
		Believing that OA is part of life	“I was extremely unhappy with myself … I couldn't work as hard as before, and I just could not understand why. It was one of the hardest things, to accept myself as what I had become.” [21] “Oh look, I've had it for so long, I just, it's just part of life. It's a limiter, but just puts boundaries on things.” [35] “So, unfortunately, it had no effect because I [my hip] is so terribly bad and consequently, I could not walk as much as I would like.” [45]
		Exercising affects other body areas	“I thought it was doing me OK but then no, I just couldn't deal with it anymore … I ended up having to have injections in my hips afterwards. Because I do have bursitis in my hips, so it actually created more problems for me.” [35] “You really need to have a strong back to start this because you're going to be doing exercises and your back has to be strong.” [37] “I had a shoulder issue at some point and so I just went and saw about that, and I had bursitis of the hips.” [38]
		Lack of access to information	“There are many 60-year-olds who don't use computers to get information. And these are the people with arthritis! I think it is much easier to get information to the younger people. We use the Internet.” [21] “It is useless to start doing physiotherapy/exercise if I am undertaking surgery in a month.” [48] “Well nobody knows about the GLA:D program.” [37]
		Fear of feeling pain	“I'm always in pain and agony, every movement is a chore. Sometimes, I just stare at my stairs, dreading what comes next.” [47] “I was advised to walk but if my knees hurt, I would stop walking.” [49] “I feel much better but I am afraid the pain will be back again once my sessions finish.” [49]
		Increasing weight can affect OA symptoms	“I feel it right away if I gain a pound; I feel it in my hips and knees.” [21] “Well, if you don't move, you get fat, no matter how little you eat.” [21] “Because when you've got knees like this, you like to do other things, you think I'm gonna go—I'd like to get back to how I was before, but I don't think that's ever going to happen now. I'm sure the weight is the biggest problem. …. .I don't eat as much as I use to, nowhere near and I was slim then. But I love me food so.” [52]
		No expectations	“I guess when I signed up, I didn't have an expectation. I thought, you know, anything's better than nothing.” [35] “I was hopeful but I wasn't unrealistic. So I didn't expect, I did not expect a miracle.” [35] “I think the other reason I probably fell down a little bit on doing the work, the exercises, was that I think after a point I wasn't convinced that even though I knew strengthening would help, I wasn't convinced that it would allow me to change my lifestyle back to what it used to be” [22]
**Social support Systems**	Relationship with the health professional	Good communication skills to explain exercises in detail	“I think he was really good. He was very easy to talk to, get along with. I think he explained everything really well.” [35]
		Good listening skills to take on board every aspect and put at ease patients	“[My physical therapist] was really good. Really approachable and listened, and really took on board whatever I said as well.” [35] “I mean, he always had time to talk to you, and say, you know, ‘Any questions or anything?’ He didn't rush you in and rush you out or, like, you know, it does happen sometimes but, with people, but, no, he was very good.” [53] “Well, I always say that my physical therapist is as good as any psychologist.” [21] “The coaching was very pleasant, very nice conversations in the evening. The physio [physical therapist] was possibly a bit business-minded or a bit focused on the work.” [36]
		Being guided by the physiotherapist to understand what to do	“I would prefer to use the gym at the hospital with a therapist.” [49] “The physiotherapist determined the gradual increase of the exercises; he told me, for example, to increase the exercises by 5 min. I liked it that he told me what to do, nevertheless, he was my physiotherapist.” [43] “I'd like to know how much attention you'd get because you can be doing something, you can attend the program and be doing it wrong.” [37] “It is that someone is there to guide you and make sure you do the right things. They ensure that you get it done. Because I know myself well enough to know that I won't do it at home.” [51]
		Connecting with the physiotherapist	“By going seeing someone every week for a period of time, I think you, you develop some trust, some openness comes from the … from my part, comes from that as well, some understanding.” [53] “I felt comfortable with talking to the Physio and built a good rapport with him. It was easier to perform the tasks so that he could see what I was doing.” [40]
		Fear of being scolded by the physiotherapist	“I guess the fact that I suppose I knew somebody was going to be marking my homework so to speak meant that there was that element as well. If I skip a day or whatever, what's [my physical therapist] going to say?” [35] ^“^The fact that I was going (to the physiotherapist) was encouraging to me because I had to produce my worksheets and he'd have a look through it and say yes yeah. And you could see what I was doing … It was also important that I could sort of show the physio that I achieved something you know. And I mean anyone can write in the sheets a figure but you can't cheat the Fitbit.” [39]
		Going to the physiotherapist to do the exercises and be motivated	“If it wasn't for a programme that someone was going to use the results, well, I probably would've thrown the towel … some sort of motivation to do it is the biggest thing, which is probably having ongoing contact with a physio or something, maybe, cracking the whip sort of thing” [37] “I think that physical therapists are the best to help those who have a physical dilemma to start exercising … and start carefully, and under supervision. I think that is very important” [21] “The most important thing is listening to the physio [physical therapist] and doing the exercises because he motivated me to do the exercises. It was for my benefit, right, so he kept on pushing me, ‘You have to do it, gradual buildup, don't go at once, start slowly, work yourself up through the stage,’ and that advice motivated me to do it.” [36] “The thing is you do the exercise ‘cause you feel that you don't want to let the other person down. You know you do them ‘cause in the first instance you think, ‘Oh that's going to do me good, it's going to yeah’, but also there's a secondary thing there you think, ‘Oh he's gone out of his way to explain these things to me and shown me what to do it's only fair that I do them so at least I can tell him what sort of effect its having the next time I meet him’, you know.” [53]
		Having external support	“I could do it with someone's support - but as soon as the study ended I just sort of dribbled off and I stopped doing it - I can't seem to self-motivate without that outside support” [37] “I was surprised I was committed to it, but part of it was because I felt like I didn't want to let the program down.” [37] ‘It's the physical seeing someone that reminds you no, you've not got the angle right or you're not holding enough.’ [39]
		Negotiation with the physiotherapist to reach a consensus about the exercises	“When I first started, it hurt a lot; the first lot of exercises. And it made it that every time I took a step it felt like someone stabbed me in the front of the kneecap with a knife; it was that sharp. And then I went back to [the physio] for my next visit and he changed one of the exercises because it was irritating the knee … and when he changed that one, even though we still did the same exercise but minus the band, it was much better …” [35] “The approach of the physiotherapist was very democratic, which I appreciated. Together, we discussed the activities and the increase of the activities. I could indicate to what extent I wanted to increase the activities, to what extent I could maintain the exercises” [43] ‘Definitely what worked for my body was the exercises when the physio and I found the ones that were not aggravating pain for me.’ [39]
		Physiotherapists showing trust in patients' capabilities	“[the physical therapist] had, I think, more faith in what I could achieve than what I did first and it was right. I could achieve it because I continued on and trusted him.” [37] “I would say that this is a bloody hard exercise and he'd say “well, just get on with it” sort of - I didn't resent what was going on, it was some parts were difficult.” [37]
		Physiotherapists pushing patients' limits	“the physiotherapist challenged me to up the weight, like rather than just keep the same weight on all the time and do more repetitions it was, actually put greater weight on.” [37] “. you have a mentor or a physiotherapist that you meet every three months or when necessary to update your exercises, steps, and the Fitbit. Help with that … Find the level, get that support. – Kind of like a diabetes nurse. - Yes, it might be like that.—OA physiotherapist.” [45]
		Flexibility to adapt the exercise programme to the patients	“I thought she was excellent. She was good at looking at what was happening and trying to change the program to fit, and I thought that she had a very positive approach …” [35] “Yes, I think the instructor was sort of aware of our capabilities and kept the challenge up. And it made it more interesting that way, because if you did the same thing over and over at the same level, it would be boring.” [50]
		Trusting the physiotherapist	“Certainly, if it's anything to do with the knee I'll seek [my PEAK physical therapist] out again and if it's to do with anything else is a very high probability that I'll seek him out again.” [35] “I thought she was very good. I thought she was very professional. And, yes, I trusted her with what she was telling me to do.” [35] “Well, if my specialist had recommended it, I would've had my decision validated.” [37]
		Need to be dedicated time or listened	“You're lucky if you'd get a quarter of an hour at our physiotherapist, don't you?” [37] “They [the physicians] are positive if you ask [for a referral to a physical therapist], but you have to ask.” [21] “They do not ask if you exercised at home.” [49]
		Passive solution with no other indication affects trust in patients	“I was told I needed some tablets. And then I went down – I saw the specialist and because the knee was such in a poor state, we virtually jumped straight into surgery.” [37] “GP? He doesn't do anything because he just prescribe the medication but nothing else.” [37] “I was having trouble with my knees every so often it did hurt you know with one thing and another. Working in the construction industry there is a lot of lifting and a lot kneeling you see and I felt well I wonder if that's got anything to do with it. So I go to the doctor and all he just simply done was put his hand on my knee, he said “move your leg, … you are getting old you've got rheumatism.” You see that was it I didn't take any more notice of it [the knee pain] …” [52]
		Lack of health professionals' support	“The physiotherapist would set up the machine and tell me to exercise then leave.” [49] “I had very little from GPs [general practitioner] or any other professionals. I'd only had a GP do couple of scans or x-rays … and that's it. No treatment, no exercises, no referrals.” [42] “I probably haven't received any education [about exercises]. I mean the only thing the surgeon ever said to me was, “I'll see you when you're ready.” [42]
	Social Aspects	Preferring to exercise independently	“I like being free when it comes to training time and just decide for myself when I do it and when I don't.” [21]
		Having a partner who actively participates in exercising and making new proposals	I've always asked her if she thought that this exercise we were doing would work for both of us, or did she need to do something else. She would think of different exercises we could do, and I'd participate with her. I'd think of stuff we could do, and she'll participate with me.” [44] “Not really negotiate … I'll make the offer and sometimes he joins me and sometimes he doesn't.” [44]
		Having a partner or relative who supports and motivates you	“… we do a lot of it together instead of separate because it's easier. We find it's easier if we're both doing it. Then you both want to go do it. If we try to do it separately, that's when the old habits creep in. You think of some reason and you do not want to go do it. With the other one it helps motivate you to keep doing it.” [44] “Because it makes a difference. It's more motivating when you're doing it [exercise] with someone else. It's easy to go back to your regular of doing nothing. I mean, if it's just me, I don't, I don't care about me. [laughs] But it's different, I'd care about someone else.” [22] “When you've got somebody there to encourage you and say, ‘Come on, let's get it done,’ you know? It helps a lot. Every day you might not feel like getting out of that door, but my wife will say, ‘Come on, get them bones loose, let's go.’ We stayed at it, and we stay at it.” [44] “It [the experience of lack of support] was, just, what should I say, totally pathetic … I guess men are not all equally understanding.” [21]
		Mirroring the others to understand if you're performing the exercises correctly	“I knew exactly what the expectation was in terms of getting it [the exercise] done correctly and for safety's sake. I certainly didn't want to be injured. So just being here with you guys watching closely … the movements that I was making, correcting them when [they] needed to be corrected, constantly – it made a big difference, ‘cause to ghome and not be sure exactly how to do … the steps or the movements for safety's sake, would've been a cause for concern, I would be like, ‘Am I doing this right?’ You know, and that in itself for me is stressful. I don't want stress.” [22]
		Exercising in a group is a good motivator	“I think it is the best exercise class I've ever attended.” [21] “Well, some people would, say … “I got fifteen pounds of weight on my [leg],” and I said, “Oh my gosh, how do they do it? I can't even do four.” [laughs] “And they can do that many?” I said, “Oh, my, maybe I can do that too, eventually” [22] “Being with the group of elderly people … of same age and we related to so many things that we did, you know. We talked about what helped us and what didn't help us, you know?” [50] “I also think that because they see you trying, it motivates them as well.” [53]
		Exercising in a group is like having a sense of obligation	“Just having that consultation and someone working with you along the way, there's a sense of obligation to yourself and to the other.” [35] “Well, it's a commitment, and if you don't show up, then the group notices.” [22] “Yeah, so it's just funny little things that keep you thinking you have a responsibility to attend ‘cause someone's gonna miss you.” [50]
		Feeling as a weight for the others	“I've got one complaint and it is only really my complaint. It was that most of them could manage so much quicker than me.” [50] “He's more active … He walks faster. He'll slow down for me, so usually if he walks with me and does stuff for me, that's really not his routine. He does it with me just to be with me because it makes him happy to see me get out and do some stuff. If he does it with me, it's more a side thing for him versus a routine.” [44]
		Exercising only if the partner is exercising too (never alone)	“If I did not do it with her, I do not know if I would do it at all. It is something that we have begun doing together. Although, let me back up. She does it with or without me. She is much more diligent about that than I am. I generally do not do it unless I do it with her.” [44]
		Having different tastes from the partner can affect the type of exercises they can perform together	“Well, he and I don't really like many of the same things except for walking … He loves to golf and I picked up golf a little bit but we don't seem to find the time to do that much together. He definitely doesn't like Zumba or water aerobics and he doesn't like swimming at all. Really the only thing we share in common is the walking.” [44]
		Misunderstanding the partner	He would remind me, every day, to go exercise, and everything, and I always kind of took it the wrong way, that he was saying exercise because you need to lose weight. To hear him say, ‘Don't forget. You need to exercise today.’ To me, I heard, ‘You are overweight. You need to lose weight. That is what I heard. Which is probably my fault. Well, it is.” [44] “… it's a little stressful in the marriage anyway. We just got to learn how to communicate with one another. Because when I talk to him he always thinks that I'm nagging or worrying him or whatever or complaining … he's easy to get upset” [44]
		Need to have a matching partner in terms of available time, motivation, and preferences	“My husband would not allow me to exercise in a gym.” [49] “It's a lot easier to exercise by myself. Frequently, since we're both retired, and we both work part time, frequently our schedules don't match. I end up exercising by myself. When we are at the house together and I'm ready to exercise, more than likely when I say I'm going for a walk he will join me, but, like I said, many times our schedules don't jibe.” [44] “When it comes to physical activity, things that he likes to do that I don't like to. He likes to go out with his boys and have fun and whatever and I'm just not a ‘going’ person. I just stay at home. We don't agree … I go to church a lot. If they have an activity at church, I go to the activities at church, but he don't go to church.” [44]
		Preferring to perform the exercises alone if the partner tries to patronise	“I prefer to do it separately, because I do it at a different pace than my wife does … I kind of push her I kind of wonder if like, make her go a little bit sometimes a little bit too much … a lot of times she would like to do it at her own pace and both of our paces are different.” [44] “I prefer separately. Only because my partner tends to want to tell me what to do. I want to do it at my speed, and how I do it … and not be told what to do, like a child.” [44]
**Environmental Enablers**	Environmental Circumstances	Everywhere is a good spot to exercise	“It was an easy process for me, because I had everything laid out. I kept my bag right there in the kitchen, so while I'm in the kitchen, before I start my day, I would just do my exercises.” [22] “… if you didn't have time to do all of ‘em, you could just start some of them during the day, just as long as you finished it during the day, and I know I would get tired, and I said, ‘Oh no. I forgot to do the lunges,’ you know, that was the last thing, OK. But you know, if I started out in the morning [leaving her home], I was doing them everywhere … I would go quilting, and … I see the stairway [to perform step-up exercises].” [22] “It wasn't so much at home I am able to do it, it's more at work. … Perhaps not as often as I would really like to, but I can do it quite freely then, because I'm totally on my own.” [52]
		Financial burden	“Generally most costs because I'm a pensioner, my wife still working though. I have to consider it. And you come to a certain age where your body is falling apart and I need, for example at the moment, another hearing aid. There are lots of things that I need at the moment. Yes financial considerations do matter.” [21,37] “And this costs money. Walking, however, is free. Such things matter when you only have your pension” [21] “Parking around any hospital, not just [Hospital], is a nightmare and you do not want to be in a situation where you have to use the hospital parking because it costs a fortune.” [37]
		Time burden	“We are trapped into a spiral in which work, we can say, takes up a lot of energy and a lot of time, and then that time is taken away from us …” [52] “I like to walk, but I do not because of housework, the children, and winter timing” [49] ^‘^The only downside was really just going to the physiotherapy clinic every week. That was the thing—I just found—it did stress me a little, just with the commute there and back because I'm busy.’ [39]
		Logistics burden	“I would like to go to the gym but there is not one near my home and my husband would not allow me” [49] “So where I lived, it was more than an hour travel to [location] and back and I thought I'd rather spend that hour in the gym.” [37] ‘I've found because it's the physiotherapist half hour drive it's sort of not always (easy), it didn't always suit.’ [39] “(…) And I am (job title), so I do not always finish work at four o'clock. I can't just say, “Now it's four o'clock, I'm leaving”. And that's what I had to do, so there were many days when I simply could not make it down there. So it probably started a little after four. I think I would be able to attend if it started at five, for example” [51]
		Accessibility issues	“Walking upstairs is the worst thing for me” [21] “Whether my daughter's got the time to be taking me to all of this.” [37]
		Unpredictable life events affecting daily routine	“She'd had a car crash. She herself, her leg's badly damaged. So she had got an insight into sort of what it was all about, you know.” [53] “So many things happening … The boys used to come in from school or work … people come and see [wife] and ugh… I'm out twice at least a week to band practice and I have two engagements as well.” [52] “Kept it up right up until most probably just before I left. And then I was packing. So it was more a time factor, and I was – the packing – I had to do most of it myself. And I was really struggling … So there really was no thought of, “Oh yes, I must do my exercises,” because by the end of the day I could barely move. So yes, I dropped off a fair bit in that few months leading up to moving.” [35]
	Technological support	Wearable devices and monitoring can help you monitor and reach your goal	“I'm amazed at how controlled I am by it, 7000 Steps, it was like, that's what I walked every day. And now that I don't have this [the WAT] anymore, I don't think I take that many steps anymore. I'm really affected by it.” [45] “a little person on my wrist … a little friend.” [46] “I mean I've got access to the videos too if I get a little bit stuck.” [42] “Never having done care remotely before, I was unsure as to the effectiveness, but it worked well.” [40] ‘I think the Fitbit probably helped, it actually became a bit of a, almost a motivation to do more, if I had 3000 steps at the end of work, well, I was probably more likely to get it up to five.’ [39]
		The role of reminders	“It's positive that it beeps when you haven't walked 250 steps in an hour. When it “beeps” you get to move and take a turn in the corridors at work …” [45] “I mean, I think that's something incredibly important, I would love you to keep calling me. And I hate them, I hate the um, the what-do-you-call-it voice. I hate the idea of it [laughs], I mean I, because I [laughs], I hate that the whole thing was happening to us all the time … the automated voices [laughs]. But I really appreciate it.” [22] ‘The Fitbit probably influenced me a little bit in terms of like the 10,000. Or if it was buzzing at the 250 and I wasn't in a meeting or there wasn't a clear reason, then I probably would be a quick reminder to move.’ [39] “That was kind of a good little challenge to have your little [activity tracker] on your arm and see how many steps and you know if you need to go for an extra walk, well, I would.” [35]
		Devices easy to use	“I'm not the smartest computer user in the world, but if I can do it, I reckon anybody can do it.” [41] “It's just simple [41].” “Sometimes with the SMS, I'd put the letters and the things around the wrong way … it was very particular, you know, you had to do it in the right … But other than that, no worries at all.” [41]
		Wearable tracking your movements can make you understand that you have bad attitudes	“Shows perhaps an inherent bad attitude.” [46] “I wasn't invested that I absolutely had to do, come hell or high water, these steps so I'd be like yeah, I just didn't walk very much today.” [46]
		Wearable are considered annoying for the reminders	“It probably gives me some incentive to walk a little further just to placate the Fitbit … I forget to check but I think it does give you an incentive to get out and do something because it's there and it nags you … I'm fine with that … it's probably a good thing to have something that makes you get up and go.” [46] “it was a reminder of the bleeding obvious.” [41] “When I got the planter fasciitis and the texts were coming through … they just kept coming, and it was kind of like a little shame thing.” [41]
		Knowing to be constantly monitored can increase pressure in patients	“But for me, the problem was that I went through a transition. I went through a transition from work, from workin' in the office, to workin' at home, and I'm on the phone, and on the computer, and I'm always on the phone all day for, for almost, for pretty much 8 h. So at the end of the day, I'm talked out. I don't wanna be bothered [with the BOOST-TLC].” [22] “At the beginning, it's very encouraging, but after a while, it's kind of, to me it's kind of tedious.” [22] “You get disquiet if you do not reach 7000 steps … I think it happened to me one day and that was very tough …” [45]

#### 3.4.1 Analytical theme 1: Mind-Body Connection

The first analytical theme, “Mind-Body Connection,” examined how the mind influences adherence positively or negatively. This theme was generated from the descriptive themes “The Role of Beliefs,” “Individuals' mindset”, and “The Experience of the Exercise Programme.”

#### 3.4.2 Descriptive theme 1 – the role of beliefs

This theme focuses on people's beliefs about the role of exercise in OA. In the primary studies, participants emphasised the significance of their views on the usefulness of exercise for OA and how this belief could impact their adherence [36,37,42,45,47,51]. They especially highlighted that strengthening muscles and exercising could improve OA symptoms and provide other benefits [21,35,37,43,48,49,52,53]. They also believed that having an active lifestyle could benefit them [21,35,48,52]. The participants appreciated that the healthcare professionals indicated the expected benefits of pursuing an exercise regimen [38]. Conversely, a lack of education or education based on outdated concepts (such as the mechanical concept underlying the symptoms of OA) reinforced negative beliefs about OA and exercise [37,38,48,50]. Some studies have shown that some people believe that exercising causes other body parts to deteriorate or that improvement is impossible because the joints are already worn out [35,37,38,47,49,52]. Specific beliefs about pain had an impact on someone's levels of exercise adherence. Individuals accepted that pain is part of their condition and that they should move anyway [35,43]. Others feared feeling pain, limiting their ability to perform exercises and everyday life activities [42,47,49,52].

#### 3.4.3 Descriptive theme 2 – individuals' mindset

Within this theme, the term mindset referred to the “way of thinking about things”. In particular, primary studies underlined how important a positive attitude is in managing OA and dealing with an exercise programme [21,35,37,38,51]. Participants stressed the importance of a positive mindset in adhering to exercise regimens. This mindset is characterised by motivation and willingness, understanding that symptom improvement requires active participation, identifying a goal and trying to achieve it without frequently taking medications [37,45,52]. A positive mindset was also characterised by the willingness to find the time to exercise in someone's routine. Some participants believed that it is not difficult to carve out time to carry out the exercise programme, finding a way to insert it into the daily routine [35,37,52]. Conversely, lacking motivation led to a loss of commitment and a progressive accommodation within daily life [21,22,35,37,45,48,50]. Finally, participants also highlighted how some psychological issues interfere with approaching an exercise programme [35].

#### 3.4.4 Descriptive theme 3: the experience of the exercise programme

The primary studies indicated that certain factors specific to the exercise programme could enhance adherence. Participants appreciated having a structured and personalised exercise programme that allowed progression, making them feel stronger and more flexible [35,36,42]. Experiencing a structured exercise program can assist individuals in learning how to self-manage their condition [39,41,45,52]. Participants adhered more if they enjoyed exercising and benefitted from it [21,36,37,48,49,52]. The exercise programme experience produced a sense of self-blame for not following it and concerns about wasting physiotherapists' time and not reaching the desired goal. Despite appearing negative, these factors motivated participants to persist with their exercise routine [35]. However, primary studies identified several factors related to the exercise programme that could be potential barriers to adherence. Some participants ceased exercising either upon feeling improvement in their condition or due to a gradual loss of interest over time [35,43,52]. Others reported that the exercise experience was mentally and physically exhausting, also because they felt too much pain [39,47,50].

#### 3.4.5 Analytical theme 2: Social Support Systems

The second analytical theme, “Social Support Systems,” highlights the role of social aspects of exercise and related factors in relationships with healthcare professionals. This theme was generated from the descriptive themes “Social aspects” and “Relationship with the Health Professional.”

#### 3.4.6 Descriptive theme 4: social aspects

This theme encompasses the social aspects that impacted exercise adherence, such as exercising with a partner and participating in group classes. Participants in the primary studies emphasised how they enjoyed having someone to exercise with. For example, exercising with a supportive partner increases motivation to exercise adherence. Some participants also reported that having an active person who proposed new activities was stimulating and challenging [22,44]. However, some participants experienced exercising with a partner as a barrier, as they felt like a burden to their partner or that their preferences in exercising were too different [44]. Some participants reported that they appreciated group classes since the context helped them to mirror others to ensure correct exercise form and motivate them to exercise in a group while progressing together and sharing the enjoyment [22,[49], [50], [51]]. At the same time, this context was not helpful for some participants as seeing people around them progress quicker than them brought negative feelings. Aligning preferences, progression, and exercise modalities among different people was challenging [50].

#### 3.4.7 Descriptive theme 5 - relationship with the health professional

This theme highlights the factors related to the relationship between the healthcare professional and the patient that affect exercise adherence. From participants' perspectives, good communication and listening skills fostered the relationship with the health professional and ultimately facilitated exercise adherence by feeling understood and supported [39,51]. These skills were also valuable for explaining exercise thoroughly and getting the preferences and goals of patients to develop a shared-personalised exercise programme [35,40,43,50]. Positive mutual trust between the healthcare professionals and the participants was perceived as a requirement and facilitator as it helped to stay motivated and navigate challenges [35,53]. In this sense, it was fundamental for the healthcare professional to show trust in patients' capabilities, and participants had to trust what the healthcare professional asked, ultimately adhering to the exercise programme [35,37]. Conversely, a lack of support from healthcare professionals or the suggestion of only passive solutions was identified as a barrier to exercise adherence. When individuals do not feel supported or when the interventions lack active exercise, their motivation to continue exercising diminishes [37,42,49,52].

#### 3.4.8 Analytical theme 3: environmental enablers

This final theme, “Environmental Enablers,” examines the environmental and technological factors that affect exercise adherence. It was generated from the descriptive themes of “Environmental Circumstances” and “Technological Support.”

#### 3.4.9 Descriptive theme 6 – environmental circumstances

This theme explores the factors surrounding the individuals able to affect exercise adherence, such as logistics, material time, economic resources, and daily unforeseen events. In particular, participants reported that reaching the place where the exercise is carried out is not always easy due to distance or accessibility (e.g., too many stairs before reaching the place) [21,37,39,49,51]. At the same time, time availability could also affect exercise adherence [37,39,40,48,49,52]. Only two studies reported that exercise can be done anywhere and anytime, improving adherence [22,52]. Another relevant factor that can affect exercise adherence is the economic resources of participants, as high costs may prevent individuals from joining exercise programmes [21,37,41,42,48,50]. Finally, participants in primary studies report that some unforeseen elements can appear in daily life and interact negatively with their commitments, such as weather conditions and family circumstances [35,52,53].

#### 3.4.10 Descriptive theme 7: technological support

This last theme encompasses the role of technology in supporting exercise adherence. Participants emphasised how smartphone reminders were perceived as valuable for sustaining motivation and engaging individuals in exercise or avoiding prolonged inactivity [22,39,40,42,45,46]. Participants also highlighted other relevant technological factors, such as monitoring physical activity, supporting goal achievement, and providing positive feedback [45,46]. These features were considered instrumental in promoting adherence to the exercise programme. However, in some cases, constant monitoring led to decreased adherence, as individuals felt data undervalued their efforts and became frustrated or pressured by reminders and notifications [22,46]. Few participants also reported that technologies made them realise they could not handle the exercise programme, ultimately leading them to quit [45].

#### 3.4.11 Confidence of evidence

Table 4 reports the confidence of the evidence analysed with the CerQual approach. The three analytical themes were assessed as moderate confidence, meaning a good confidence, because of minor concerns regarding the methodological limitations, the coherence and adequacy of data within and across all studies included, and substantial concerns regarding relevance. Most studies had women participants, reflecting the higher prevalence of OA in women than men, and therefore, this constituted a minor concern not strongly impacting the confidence of our results.

**Table 4:** CERQual qualitative evidence profile.

**Objective**: To identify, appraise and synthesise relevant articles addressing the perceived barriers and facilitators of adherence to exercise in OA							
**Perspective**: The experience of an exercise program in people with hip and knee OA, defined by a healthcare professional, that explored the perceived barriers and facilitators to exercise adherence							
**Included program**mes: We intended any planned, structured and repetitive physical activity.							
**Review Finding**	**Studies Contributing to the Review Finding**	**Assessment of Methodological Limitations** [1]	**Assessment of Relevance** [2]	**Assessment of Coherence** [3]	**Assessment of Adequacy** [4]	**Overall CERQual Assessment of Confidence** [5]	**Explanation of Judgment**
Mind-Body Connection	Studies [21,22,[35], [36], [37], [38], [39],[41], [42], [43],^45^,[47], [48], [49], [50], [51], [52], [53]]	Minor methodological limitations (four studies with no, eleven with minor, and three with moderate methodological limitations)	Minor concerns about relevance (thirteen studies had most women; there were no studies in African continent and four did not report the nationality of participants; the exercise setting varied among studies, with the majority being in clinical and home settings; some studies did not specify the exercise programme)	Minor concerns about coherence (data consistent within and across all studies)	Minor concerns about adequacy (studies [21,35,37,38,42,53] together offered high rich data overall)	**Moderate confidence**	This finding was graded as moderate confidence because of minor concerns regarding methodological limitations, relevance, coherence, and adequacy
	1. Was downgraded to minor concern as the most of studies were not with ‘no methodological limitations’, but with minor methodological limitations; 2. Was downgraded to minor concern as the studies included all addressed our phenomenon of interest, but there were some differences in the nationality of participants, and in the exercise setting. Even if most of participants were women, that did not impact our finding because it reflects the prevalence of OA; 3. Was downgraded to minor concern as the studies presented consistent results when exploring the role of beliefs, mindset and the experience of exercise programme, with slight differences among the experiences of exercise programmes; 4. Was downgraded to minor concern as all studies presented a consistent quantity of data, but some studies presented high rich data; 5. Was downgraded of one due to the complete absence of concerns among the previous four domains.						
Social Support Systems	Studies [21,22,[35], [36], [37],^39^,^40^,[42], [43], [44], [45],^47^,[49], [50], [51], [52], [53]]	Minor methodological limitations (three with no, nine with minor, and five with moderate methodological limitations)	Minor concerns about relevance (fourteen studies had most women; no studies in African continent; the exercise setting varied among studies, with the majority being in clinical and home settings; some studies did not specify the exercise programme)	Minor concerns about coherence (data consistent within and across all studies)	Minor concerns about adequacy (studies [21,35,37,44,53] together offered moderate rich data overall)	**Moderate confidence**	This finding was graded as moderate confidence because of minor concerns regarding methodological limitations, relevance, coherence, and adequacy
	1. Was downgraded to minor concern as the most of studies were not with ‘no methodological limitations’, but with minor methodological limitations; 2. Was downgraded to minor concern as the studies included all addressed our phenomenon of interest, but there were some differences in the nationality of participants, and in the exercise setting. Even if most of participants were women, that did not impact our finding because it reflects the prevalence of OA; 3. Was downgraded to minor concern as the studies presented consistent results when exploring the role of social aspects, and relationship with the health professional, with slight differences related to the social aspects (e.g., exercising alone or in group); 4. Was downgraded to minor concern as all studies presented a consistent quantity of data, but some studies presented moderate rich data; 5. Was downgraded of one due to the complete absence of concerns among the previous four domains.						
Environmental Enablers	Studies [21,22,[35], [36], [37],^39^,^40^,^42^,[45], [46], [47], [48], [49], [50], [51],^53^]	Minor methodological limitations (five with no, ten with minor, and two with moderate methodological limitations)	Minor concerns about relevance (thirteen studies had most women; no studies in African continent and four did not declare the Ethnicity; the exercise setting varied among studies, with the majority being in clinical and home settings) Indirect relevance as assessing technologies was not the main aim of our study, but it was considered a facilitator to adherence	Minor concerns about coherence (data consistent within and across all studies)	Minor concerns about adequacy (studies [22,39,40,46] offered moderate rich data overall)	**Moderate confidence**	This finding was graded as moderate confidence because of minor concerns regarding methodological limitations, relevance, coherence, and minor concerns regarding adequacy
	1. Was downgraded to minor concern as the most of studies were not with ‘no methodological limitations’, but with minor methodological limitations; 2. Was downgraded to minor concern as the studies included all addressed our phenomenon of interest, but there were some differences in the nationality of participants, and in the exercise setting. Even if most of participants were women, that did not impact our finding because it reflects the prevalence of OA. Moreover, this theme presents indirect relevance as it was not the main aim of our study; 3. Was downgraded to minor concern as the studies presented consistent results when exploring the role of environmental circumstances, and technological support, with slight differences related to the technology use; 4. Was downgraded to minor concern as all studies presented a consistent quantity of data, but some studies presented moderate rich data; 5. Was downgraded of one due to the complete absence of concerns among the previous four domains.						

## 4 Discussions

This thematic synthesis of qualitative studies investigated the main perceived facilitators and barriers to exercise adherence in people with OA. The analysis generated three analytical themes crucial for exercise adherence: 1) ‘Mind-Body Connection’, highlighting the role of cognitive and psychological factors; 2) ‘Social Support Systems’, emphasising the social environment's critical role; and 3) ‘Environmental Enablers’, underscoring the importance of external and environmental factors such as the settings where the exercise is performed and the use of technology.

The first analytical theme stressed the effect that ‘mind’, as an umbrella term covering people's beliefs, experiences, expectations, and mindset, has on exercise adherence. Misconceptions about pain and OA, coupled with a lack of education, often led to fear and giving up exercise programmes. In contrast, personalised goals, education about OA, and a positive mindset were identified as critical facilitators for long-term adherence. Our qualitative findings align with preliminary quantitative evidence indicating that improving mindsets about exercise and behavioural change techniques might increase adherence [54,55].

The effectiveness of these strategies is often linked to contextual factors such as communication with clinicians and feeling cared for by them and relevant others, which align with the second analytical theme, ‘Social Support Systems’ [56,57]. This second theme explored social contexts from two sides: (i) the professional context, where effective communication and empathy from healthcare professionals enhanced motivation and commitment, and (ii) the personal context, where exercising with a partner or group fostered motivation through shared goals and community support. Conversely, misalignment in preferences or progression within the patient and their healthcare professionals, partners, or exercise groups could negatively affect adherence. Other findings suggested that support from a group (family, friends and professionals) can contribute to long-term adherence [58]. These groups must be cohesive to improve adherence, showing similarity in salient attributes [59]. As highlighted in our study, these factors can either undermine or increase the success of an exercise programme. Contextual factors, such as communication, the ability to motivate, and the social environment, can positively influence outcomes through a placebo response. However, the opposite effect, known as the ‘nocebo effect,’ can also significantly jeopardise individuals' results, and clinicians should be aware of it [60].

The third theme, ‘Environmental Enablers’, explores how contextual factors extend beyond patients' relationships, including external factors like time constraints, financial limitations, unforeseen events, and mixed perceptions of technology. Lack of time is a commonly cited barrier in the literature [61,62], yet research suggests this is often a perception rather than an actual phenomenon [63]. Heesch et al. highlighted a gap between perceived and actual lack of time, underscoring the importance of addressing this misconception during patient education [63]. Financial limitations also play a significant role, especially for people in lower socioeconomic positions who have less access to OA interventions compared to those who can afford private options [64]. As a result, people in lower socioeconomic positions experience reduced benefits from OA interventions, more severe symptoms, and higher OA incidence, raising the need for a call to action at higher healthy-policy levels [65]. Unforeseen events, like changes in weather or family obligations, also impacted adherence to OA treatment routines. Exploring whether teaching patients flexible strategies for maintaining their routines could help them adapt and find alternative ways to exercise despite unexpected disruptions may be valuable. Finally, technology was perceived as both a facilitator and a barrier. Some participants appreciated monitoring tools and reminders as they boosted motivation [66]. Conversely, other studies reported that reminders caused them to feel annoyed and stressed. According to the literature, positive perceptions about digital technologies are more common among younger generations compared to older ones, likely due to greater digital literacy, which helps to correctly and critically use technology [[67], [68], [69]].

To conclude, our thematic synthesis, compared to previous research, emphasised the role of the relationship between the healthcare professional and the people doing exercises in terms of soft skills such as empathy and effective communication techniques. Additionally, our findings highlight the role of technology in enhancing or reducing exercise adherence was in people with OA, which role was instead known in other chronic conditions [14,25]. Beyond these novel insights, our findings resonate with existing reviews on barriers and facilitators to exercise adherence. The role of beliefs, experiences, expectations, education and exercise programme personalisation in influencing exercise adherence is common among people with OA and the general population [14,25]. Also, social support and contextual factors, such as economic availability, flexibility in time, and an intimidating gym environment, have been emphasised as key determinants in exercise adherence [23,70,71]. A pragmatic, patient-centred approach, rooted in shared-decision making, can help overcome exercise adherence barriers by exploring patients' preferences from the outset, considering all identified factors, and fostering a collaborative negotiation to develop a mutually agreed exercise programme. Engaging people in shared-decision making ensures that their values, concerns, and goals are integrated into the process, enhancing their commitment [72]. In this context, strategies such as motivational interviewing could be valuable in fostering behavioural changes and improving adherence to OA exercise programmes. However, evidence on the use of motivational interviewing in exercise adherence is still limited, and further research is needed [73].

Few limits need to be declared. Given the qualitative nature of included studies, standardisation was impossible, as data collection methods varied. Most studies were from economically developed countries, reducing the experience variability. The exercise types also varied among the studies. Nevertheless, the varying approaches, cultures and exercise modalities revealed similar perspectives regarding the exercise experience. Our studies drew on diverse theoretical underpinnings, a common challenge in synthesising qualitative research [74]. To ensure rigour, we adopted several strategies. We began with a well-defined research question and selected studies based on specific criteria relevant to addressing it. Our multidisciplinary research team, comprising physiotherapists, sport scientists and occupational therapists, helped capture the distinct nuances of the primary studies. Moreover, our study focused on a broader definition of exercise programmes, not only on exercise provided during Osteoarthritis Management Programme (OAMP) programmes delivered only in a few countries. Future studies may focus only on the experience of OAMP programmes. In some studies, the OA diagnosis was self-reported, but they all reported to perform an exercise programme specific and tailored made for OA [22,44,45,50]. It must be noted that the presence of comorbidities may have affected the results of individual studies. Future research should compare findings between people with OA and comorbidities and those with OA alone. Finally, five of the studies included came from the same Australian research team [35,36,38,40,41], but with different members. These studies were conducted on different samples with OA, had different contexts (e.g., responders vs non-responders, telephone coaches, comorbid obesity, and eHealth intervention), and according to CASP, four had minor concerns [35,36,38,41], while one had moderate concern [40]. Therefore, we are confident that the inclusion of these studies has not compromised the confidence of our findings. Despite the varied approaches, the studies revealed a shared understanding among participants. The strengths of our research include rigorous methodology, collaboration with a librarian, and the use of the CerQual tool to assess the confidence of our findings. Future research may investigate the experiences of individuals in less developed countries and analyse other joint districts to explore and share similar patterns of meaning.

## 5 Conclusion

This study underscored the complex interplay of cognitive, social, and environmental factors that influence exercise adherence in individuals with OA. The findings reveal that a ‘one-size-fits-all’ approach is inadequate for fostering long-term exercise engagement, as personal motivations, social support, financial constraints, and environmental conditions (among others) all play significant roles in adherence. These insights emphasise the need for more nuanced, individualised strategies that consider the diverse challenges and facilitators faced by people with OA. Future quantitative research should expand on these findings to design targeted interventions that address the specific needs of different subgroups, ultimately improving exercise adherence and overall quality of life for those with OA.

## Author contributions

BG, SB, DM, AN and GL Concepted and designed the study. BG, SB, DM, AN and GL conducted the acquisition of data. BG, DM, AN and SB analysed and interpreted the data. BG, SB, DM, AN, YP, and AD drafted or revised the article critically for important intellectual content. All authors approved the final version of the manuscript.

## Data statement

All data are available as supplementary materials.

## Role of the funding source

Authors should declare the role of study sponsors, if any, in the study design, in the collection, analysis and interpretation of data; in the writing of the manuscript; and in the decision to submit the manuscript for publication. If the study sponsors had no such involvement, the authors should state this.

## Declaration of competing interest

Nothing to declare.
