# Supplementary material for: Perceived barriers and facilitators to exercise adherence in osteoarthritis: A thematic synthesis of qualitative studies
Source: Osteoarthr Cartil Open. 2025 Feb 15;7(2):100584. doi: 10.1016/j.ocarto.2025.100584 (PMC11889972; doi:10.1016/j.ocarto.2025.100584)
Supplement: Multimedia component 1 [file mmc1.docx]

**Supplementary Material 1**: Research Strings

Sample, Phenomenon of Interest, Design, Evaluation, Research type tool (SPIDER) was applied to create the research strings^1^.

S: People with OA

PI: Barriers/Facilitators to exercise adherence

D: questionnair*, surve*, interview*, focus group*, case stud*, observ*

E: attitud*, emotion*, perception*, experienc*, opinion*, belie*, understand*, feel*, view*, know*

R: qualitative, mixed method

| **SPIDER Research String via Pubmed** | |
| --- | --- |
| **Simple** | "Osteoarthritis"[Mesh] OR Osteoarthritides OR Osteoarthrosis OR Osteoarthroses OR Degenerative Arthritides OR Degenerative Arthritis OR Arthrosis OR Arthroses OR Osteoarthrosis Deformans |
| **“AND”** | |
| **Phenomenon of Interest** | "exercise"[Mesh] OR exercis* OR "exercise therapy"[Mesh] OR “motor activity” [Mesh] OR motor activity OR motor activities OR physical activity OR physical activities |
| **“AND”** | |
| **Design** | "Surveys and Questionnaires"[Mesh] OR "Interview"[Publication Type] OR "Focus Groups"[Mesh] OR "Observation"[Mesh] OR "Nursing Methodology Research"[Mesh] OR "Hermeneutics"[Mesh] OR "Patient Acuity"[Mesh] OR "grounded theory"[Mesh] OR "Narration"[Mesh] OR Questionnaire OR Interview OR Focus group OR case stud* OR observ* OR Qualitative OR Hermeneutics OR Phenomenology OR grounded theory OR Narration OR story telling OR Storytelling OR story-telling OR Thematic Analysis OR Content analysis |
| **“AND”** | |
| **Evaluation** | "Life Change Events"[Mesh] OR "Attitude"[Mesh] OR "Behavior"[Mesh] OR "Emotions"[Mesh] OR "quality of life"[Mesh] OR "Activities of Daily Living"[Mesh] OR "Social Participation"[Mesh] OR "Patient Participation"[Mesh] OR "Knowledge"[Mesh] OR "health knowledge, attitudes, practice"[Mesh] OR "Metacognition"[Mesh] OR "Perception"[Mesh] OR "Pain Perception"[Mesh] OR "Social Perception"[Mesh] OR "self-concept"[Mesh] OR "Attitude"[Mesh] OR "Attitude to Health"[Mesh] OR "Emotions"[Mesh] OR "Behavior and Behavior Mechanisms"[Mesh] OR experience* OR opinion* OR "Quality of Life"[Mesh] OR belie* OR feel* OR attitude* OR Participation OR Emotional Involvement OR self-concept OR self-concept OR Image OR view* OR Perspective OR Perception OR feeling* OR behavi* |
| **“AND”** | |
| **Research Type** | "Qualitative Research"[Mesh] OR Qualitative OR mixed method OR multi method OR mixed methods OR multi methods OR mixed-method OR multi-method OR mixed-methods OR multi-methods |

| **Pubmed Searched on 21 October 2024 -> 1062 Results** |
| --- |
| (((("Osteoarthritis"[Mesh] OR Osteoarthritides OR Osteoarthrosis OR Osteoarthroses OR Degenerative Arthritides OR Degenerative Arthritis OR Arthrosis OR Arthroses OR Osteoarthrosis Deformans) AND ("exercise"[Mesh] OR exercis* OR "exercise therapy"[Mesh] OR "motor activity" [Mesh] OR motor activity OR motor activities OR physical activity OR physical activities)) AND ("Surveys and Questionnaires"[Mesh] OR "Interview"[Publication Type] OR "Focus Groups"[Mesh] OR "Observation"[Mesh] OR "Nursing Methodology Research"[Mesh] OR "Hermeneutics"[Mesh] OR "Patient Acuity"[Mesh] OR "grounded theory"[Mesh] OR "Narration"[Mesh] OR Questionnaire OR Interview OR Focus group OR case stud* OR observ* OR Qualitative OR Hermeneutics OR Phenomenology OR grounded theory OR Narration OR story telling OR Storytelling OR story-telling OR Thematic Analysis OR Content analysis)) AND ("Life Change Events"[Mesh] OR "Attitude"[Mesh] OR "Behavior"[Mesh] OR "Emotions"[Mesh] OR "quality of life"[Mesh] OR "Activities of Daily Living"[Mesh] OR "Social Participation"[Mesh] OR "Patient Participation"[Mesh] OR "Knowledge"[Mesh] OR "health knowledge, attitudes, practice"[Mesh] OR "Metacognition"[Mesh] OR "Perception"[Mesh] OR "Pain Perception"[Mesh] OR "Social Perception"[Mesh] OR "self-concept"[Mesh] OR "Attitude"[Mesh] OR "Attitude to Health"[Mesh] OR "Emotions"[Mesh] OR "Behavior and Behavior Mechanisms"[Mesh] OR experience* OR opinion* OR "Quality of Life"[Mesh] OR belie* OR feel* OR attitude* OR Participation OR Emotional Involvement OR self-concept OR self-concept OR Image OR view* OR Perspective OR Perception OR feeling* OR behavi*)) AND ("Qualitative Research"[Mesh] OR Qualitative OR mixed method OR multi method OR mixed methods OR multi methods OR mixed-method OR multi-method OR mixed-methods OR multi-methods) |
|  |
| **Cochrane Central Searched on 21 October 2024 -> 60 Results** |
| #1 [mh Osteoarthritis] OR Osteoarthritides OR Osteoarthrosis OR Osteoarthroses OR "Degenerative Arthritides" OR "Degenerative Arthritis" OR Arthrosis OR Arthroses OR "Osteoarthrosis Deformans" 11651  #2 [mh exercise] OR exercis* OR [mh "exercise therapy"] OR [mh "motor activity"] OR "motor activity" OR "motor activities" OR "physical activity" OR "physical activities" 170538  #3 [mh "Surveys and Questionnaires"] OR Interview:pt OR [mh "Focus Groups"] OR [mh Observation] OR [mh "Nursing Methodology Research"] OR [mh Hermeneutics] OR [mh "Patient Acuity"] OR [mh "grounded theory"] OR [mh Narration] OR Questionnaire OR Interview OR "Focus group" OR ("case" NEXT stud*) OR observ* OR Qualitative OR Hermeneutics OR Phenomenology OR "grounded theory" OR Narration OR "story telling" OR Storytelling OR story-telling OR "Thematic Analysis" OR "Content analysis" 521928  #4 [mh "Life Change Events"] OR [mh Attitude] OR [mh Behavior] OR [mh Emotions] OR [mh "Quality of Life"] OR [mh "Activities of Daily Living"] OR [mh "Social Participation"] OR [mh "Patient Participation"] OR [mh Knowledge] OR [mh "Health Knowledge, Attitudes, Practice"] OR [mh Metacognition] OR [mh Perception] OR [mh "Pain Perception"] OR [mh "Social Perception"] OR [mh "Self-Concept"] OR [mh "Attitude to Health"] OR [mh Emotions] OR [mh "Behavior and Behavior Mechanisms"] OR experience* OR opinion* OR [mh "Quality of Life"] OR belie* OR feel* OR attitude* OR Participation OR "Emotional Involvement" OR "Self-Concept" OR Image OR view* OR Perspective OR Perception OR feel* OR behavi* 525848  #5 [mh "Qualitative Research"] OR Qualitative OR "mixed method" OR "multi method" OR "mixed methods" OR "multi methods" OR mixed-method OR multi-method OR mixed-methods OR multi-methods 26155  #6 #1 AND #2 AND #3 AND #4 AND #5 59 |
|  |
| **Embase Searched on 21 October 2024 -> 614 Results** |
| ('osteoarthritis'/exp OR osteoarthritides OR osteoarthrosis OR 'degenerative arthritides' OR 'degenerative arthritis' OR arthrosis OR 'osteoarthrosis deformans') AND ('exercise'/exp OR exercis* OR 'exercise therapy'/exp OR 'motor activity'/exp OR 'physical activity'/exp OR 'exercise movement techniques'/exp OR 'movement therapy'/exp OR 'resistance training'/exp OR 'aerobic exercise'/exp OR 'strength training'/exp OR 'stretching exercise'/exp OR 'walking'/exp) AND ('surveys and questionnaires'/exp OR 'focus groups'/exp OR 'observation'/exp OR 'nursing methodology research'/exp OR 'hermeneutics'/exp OR 'patient acuity'/exp OR 'grounded theory'/exp OR 'narration'/exp OR questionnaire OR interview OR 'case stud*' OR observ* OR qualitative OR hermeneutics OR phenomenology OR 'thematic analysis'/exp OR 'content analysis'/exp) AND ('life change events'/exp OR 'attitude'/exp OR 'behavior'/exp OR 'emotions'/exp OR 'activities of daily living'/exp OR 'social participation'/exp OR 'patient participation'/exp OR 'knowledge'/exp OR 'health knowledge, attitudes, practice'/exp OR 'metacognition'/exp OR 'perception'/exp OR 'pain perception'/exp OR 'social perception'/exp OR 'self concept'/exp OR 'attitude to health'/exp OR 'behavior and behavior mechanisms'/exp OR experience* OR opinion* OR 'quality of life'/exp OR belie* OR feel* OR attitude* OR participation OR 'emotional involvement' OR 'self concept' OR image OR view* OR perspective OR perception OR feeling* OR behavi*) AND ('qualitative research'/exp OR qualitative OR 'mixed methods'/exp OR 'mixed method*' OR 'multi method*' OR 'mixed methods' OR 'multi methods') |
|  |
| **Cinhal + Psyincho Searched on 21 October 2024 -> 118** |
| (((((MH Osteoarthritis+) OR Osteoarthritides OR Osteoarthrosis OR Osteoarthroses OR "Degenerative Arthritides" OR "Degenerative Arthritis" OR Arthrosis OR Arthroses OR "Osteoarthrosis Deformans" ) AND ((MH exercise+) OR exercis* OR (MH "exercise therapy+") OR (MH "motor activity+") OR "motor activity" OR "motor activities" OR "physical activity" OR "physical activities" )) AND ((MH "Surveys and Questionnaires+") OR (PT Interview) OR (MH "Focus Groups+") OR (MH Observation+) OR (MH "Nursing Methodology Research+") OR (MH Hermeneutics+) OR (MH "Patient Acuity+") OR (MH "grounded theory+") OR (MH Narration+) OR Questionnaire OR Interview OR "Focus group" OR "case stud*" OR observ* OR Qualitative OR Hermeneutics OR Phenomenology OR "grounded theory" OR Narration OR "story telling" OR Storytelling OR story-telling OR "Thematic Analysis" OR "Content analysis" )) AND ((MH "Life Change Events+") OR (MH Attitude+) OR (MH Behavior+) OR (MH Emotions+) OR (MH "quality of life+") OR (MH "Activities of Daily Living+") OR (MH "Social Participation+") OR (MH "Patient Participation+") OR (MH Knowledge+) OR (MH "health knowledge, attitudes, practice+") OR (MH Metacognition+) OR (MH Perception+) OR (MH "Pain Perception+") OR (MH "Social Perception+") OR (MH self-concept+) OR (MH Attitude+) OR (MH "Attitude to Health+") OR (MH Emotions+) OR (MH "Behavior and Behavior Mechanisms+") OR experience* OR opinion* OR (MH "Quality of Life+") OR belie* OR feel* OR attitude* OR Participation OR "Emotional Involvement" OR self-concept OR self-concept OR Image OR view* OR Perspective OR Perception OR feeling* OR behavi* )) AND ((MH "Qualitative Research+") OR Qualitative OR "mixed method" OR "multi method" OR "mixed methods" OR "multi methods" OR mixed-method OR multi-method OR mixed-methods OR multi-methods ) |

**Reference**:

[1] Cooke, A., Smith, D., & Booth, A. (2012). Beyond PICO: The SPIDER Tool for qualitative evidence synthesis. Qualitative Health Research, 22(10), 1435–1443. https://doi.org/10.1177/104973231245293
