# Supplementary material for: Perceived barriers and facilitators to exercise adherence in osteoarthritis: A thematic synthesis of qualitative studies
Source: Osteoarthr Cartil Open. 2025 Feb 15;7(2):100584. doi: 10.1016/j.ocarto.2025.100584 (PMC11889972; doi:10.1016/j.ocarto.2025.100584)
Supplement: Multimedia component 2 [file mmc2.docx]

**Supplementary Material 2** – Analysis of the Excluded Full Texts

| **Studies** | **Main Reasons for exclusion** |
| --- | --- |
| Webber, S. C., Ripat, J. D., Pachu, N. S., & Strachan, S. M. (2020). Exploring physical activity and sedentary behaviour: perspectives of individuals with osteoarthritis and knee arthroplasty. Disability and Rehabilitation, 42(14), 1971–1978. https://doi.org/10.1080/09638288.2018.1543463 | Wrong intervention |
| Christiansen, M. B., Dix, C., Master, H., Jakiela, J. T., Habermann, B., Silbernagel, K. G., & White, D. K. (2020). “I've been to physical therapy before, but not for the knees.” A qualitative study exploring barriers and facilitators to physical therapy utilization for knee osteoarthritis. *Musculoskeletal care*, *18*(4), 477-486. | Wrong population |
| Cheung, C., Wyman, J. F., & Peden-McAlpine, C. (2022). Long-term yoga and aerobic/strength exercise adherence in older women with knee osteoarthritis: a mixed methods approach. *International journal of yoga therapy*, *32*(2022), Article-4. | No full text available |
| Poitras, S., Rossignol, M., Avouac, J., Avouac, B., Cedraschi, C., Nordin, M., ... & Hilliquin, P. (2010). Management recommendations for knee osteoarthritis: how usable are they?. *Joint bone spine*, *77*(5), 458-465. | Wrong population |
| Gay, C., Eschalier, B., Levyckyj, C., Bonnin, A., & Coudeyre, E. (2018). Motivators for and barriers to physical activity in people with knee osteoarthritis: a qualitative study. *Joint Bone Spine*, *85*(4), 481-486. | Wrong intervention |
| Wallis, J. A., Webster, K. E., Levinger, P., Singh, P. J., Fong, C., & Taylor, N. F. (2019). Perceptions about participation in a 12-week walking program for people with severe knee osteoarthritis: a qualitative analysis. *Disability and rehabilitation*, *41*(7), 779-785. | Wrong population |
| Soto, S. H., Berry, D. C., & Callahan, L. F. (2022). Qualitative Exploration of Dyadic Influence on Physical Activity Between Latina Patients With Osteoarthritis and a Supporter of Their Physical Activity. *Arthritis care & research*, *74*(2), 281-290. | Wrong intervention |
| McKevitt, S., Jinks, C., Healey, E. L., & Quicke, J. G. (2022). The attitudes towards, and beliefs about, physical activity in people with osteoarthritis and comorbidity: A qualitative investigation. *Musculoskeletal care*, *20*(1), 167-179. | Wrong population |
| Ananian, C. A. D., Wilcox, S., Abbott, J., Vrazel, J., Ramsey, C., Sharpe, P., & Brady, T. (2006). The exercise experience in adults with arthritis: a qualitative approach. *American journal of health behavior*, *30*(6), 731-744. | Wrong intervention |
| Hendry, M., Williams, N. H., Markland, D., Wilkinson, C., & Maddison, P. (2006). Why should we exercise when our knees hurt? A qualitative study of primary care patients with osteoarthritis of the knee. *Family practice*, *23*(5), 558-567. | Wrong intervention |
| Cheung, C., Justice, C., & Peden-Mcalpine, C. (2015). Yoga adherence in older women six months post-osteoarthritis intervention. *Global Advances in Health and Medicine*, *4*(3), 16-23. | Wrong outcome |
| Papi E, Belsi A, McGregor AH. A knee monitoring device and the preferences of patients living with osteoarthritis: a qualitative study. BMJ Open. 2015 Sep 7;5(9):e007980. doi: 10.1136/bmjopen-2015-007980. PMID: 26346873; PMCID: PMC4563249. | Wrong outcome |
| Middleton KR, Magaña López M, Haaz Moonaz S, Tataw-Ayuketah G, Ward MM, Wallen GR. A qualitative approach exploring the acceptability of yoga for minorities living with arthritis: 'Where are the people who look like me?'. Complement Ther Med. 2017 Apr;31:82-89. doi: 10.1016/j.ctim.2017.02.006. Epub 2017 Mar 1. PMID: 28434476; PMCID: PMC5583513. | Wrong population |
| Bossen D, Buskermolen M, Veenhof C, de Bakker D, Dekker J. Adherence to a web-based physical activity intervention for patients with knee and/or hip osteoarthritis: a mixed method study. J Med Internet Res. 2013 Oct 16;15(10):e223. doi: 10.2196/jmir.2742. PMID: 24132044; PMCID: PMC3806355. | Wrong study design |
| Evans, Georgie & Adams, Jo & Donovan-Hall, Maggie. An exploration of the facilitators and barriers for people with osteoarthritis to engage in exercise. International Journal of Therapy and Rehabilitation 2023. 182-188. 10.12968/ijtr.2016.23.4.182. | Wrong outcome |
| Park E, Park HR, Choi ES. Barriers to and Facilitators of Physical Activity among Korean Female Adults with Knee Osteoarthritis and Comorbidity: A Qualitative Study. Healthcare (Basel). 2020 Jul 23;8(3):226. doi: 10.3390/healthcare8030226. PMID: 32717904; PMCID: PMC7551821. | Wrong population |
| Kaptein SA, Backman CL, Badley EM, Lacaille D, Beaton DE, Hofstetter C, Gignac MA. Choosing where to put your energy: a qualitative analysis of the role of physical activity in the lives of working adults with arthritis. Arthritis Care Res (Hoboken). 2013 Jul;65(7):1070-6. doi: 10.1002/acr.21957. PMID: 23335584. | Wrong population |
| Drum EE, Kovats A, Jones MD, Dennis S, Naylor J, Mills K, Thom JM. Creaky knees: Is there a reason for concern? A qualitative study of the perspectives of people with knee crepitus. Musculoskeletal Care. 2023 Dec;21(4):1114-1124. doi: 10.1002/msc.1793. Epub 2023 Jun 21. PMID: 37341878. | Wrong population |
| Ledingham, A.; Cohn, E.; Baker, K.; Keysor, J. Exercise and adherence over two years: Beliefs of adults with knee osteoarthritis | No full text |
| Gell NM, Smith PA, Wingood M. Physical Therapist and Patient Perspectives on Mobile Technology to Support Home Exercise Prescription for People With Arthritis: A Qualitative Study. Cureus. 2024 Mar 10;16(3):e55899. doi: 10.7759/cureus.55899. PMID: 38601402; PMCID: PMC11006223. | Wrong population |
| Matile F, Nast I, Niedermann K. Facilitators, barriers and support needs to GLA:D exercise adherence - a mixed method study. BMC Sports Sci Med Rehabil. 2024 Jun 13;16(1):130. doi: 10.1186/s13102-024-00913-6. PMID: 38872226; PMCID: PMC11170889. | No quotes |
| Gustafsson K, Areskoug Josefsson K, Eriksson M, Rolfson O, Kvist J. Perspectives on health care and self-management of osteoarthritis among patients who desire surgery: A qualitative interview study. Physiother Theory Pract. 2024 Aug;40(8):1784-1794. doi: 10.1080/09593985.2023.2215302. Epub 2023 May 29. PMID: 37246837. | Wrong intervention |
