# Supplementary material for: Perceived barriers and facilitators to exercise adherence in osteoarthritis: A thematic synthesis of qualitative studies
Source: Osteoarthr Cartil Open. 2025 Feb 15;7(2):100584. doi: 10.1016/j.ocarto.2025.100584 (PMC11889972; doi:10.1016/j.ocarto.2025.100584)
Supplement: Multimedia component 3 [file mmc3.docx]

**Supplementary Material 3** – Primary Studies’ Themes, Subthemes and Quotes

| **STUDY** | **THEMES** | **SUBTHEMES** | **QUOTES** |
| --- | --- | --- | --- |
| **Hinman et al. (2023)**  **“Absence of Improvement With Exercise in Some Patients**  **With Knee Osteoarthritis: A Qualitative Study of Responders**  **and Nonresponders**” | **Facilitators** | Accountability and  monitoring | *“I guess the fact that I suppose I knew somebody was going to be marking my homework so to speak*  *meant that there was that element as well. If I skip a day or whatever, what’s [my physical therapist] going*  *to say?”*  *Lesley: “Just having that consultation and someone working with you along the way, there’s a sense of obligation to yourself and to the other person.”*  *Gregory: “That was kind of a good little challenge to have your little [activity tracker] on your arm and see how many steps and you know if you need to go for an extra walk, well, I would”*  *“When I was filling out the booklet all the time, that was an incentive to make sure I kept doing the exercises. And I’ve noticed since I haven’t got to fill it out all the time, I’m not doing them 3 times a week, I’ve let it slip…I was using the stepper all the time – yes, I used the stepper every day. Even when I wasn’t doing the exercise and that thing, I was putting it on every day and measuring my daily steps. That was good actually, that was a real motivator.”*  *Michelle: “I found going to [the physio], it made you do it.”*  *David: “Well, it just gave you something to work on. Yeah, look, it was something that I used to do every night before I went to bed, I’d fill it in…I thought that was good.”* |
|  |  | Individualization | *“I guess certainly the physio treatment and the tailoring of the selection of the exercises and the ability to have that reviewed on a regular basis and ratchet it up accordingly [helped achieve results].”*  *Nancy: “I was able to negotiate away from the ones that were awkward or difficult for me, to the ones that were easier or more present or physically possible in my house.”*  *Melissa: “You know, I think there was a couple that, they were hard to do and we just adjusted them and then worked towards the harder ones…I liked that you could build on it, so you weren’t expected to just, you know, do a mammoth effort in the beginning.”*  *“With [the physio], we sort of worked on trying to not do it – not go down as far, and so just played that one by ear as to a point where you were continuing to do them, but not to the full degree that it was originally required.” Michelle: “When I first started, it hurt a lot; the first lot of exercises. And it made it that every time I took a step it felt like someone stabbed me in the front of the kneecap with a knife; it was that sharp. And then I went back to [the physio] for my next visit and he changed one of the exercises because it was irritating the knee…and when he changed that one, even though we still did the same exercise but minus the band, it was much better…” Kathleen: “She changed a couple of them because I just said to her, “I can’t squat down on that chair, it just doesn’t happen,” and she did change a couple of them around for me…”* |
|  |  | Positive therapeutic  relationship | *“Certainly if it’s anything to do with the knee I’ll seek [my PEAK physical therapist] out again and if it’s to do with anything else is a very high probability that I’ll seek him out again.”*  *Lesley: “I think he was really good. He was very easy to talk to, get along with. I think he explained everything really well.”*  *Melissa: “[My physical therapist] was really good. Really approachable and listened, and really took onboard whatever I said as well.”*  *“I thought she was excellent. She was good at looking at what was happening and trying to change the program to fit, and I thought that she had a very positive approach…”*  *David: “I must say, in the past I haven’t been all that fussy about physios, because I didn’t feel that they were as hands-on as I would like, but he seemed to be – yeah, sort of easy to talk to and understood the problems quite well.”*  *Joyce: “I thought she was very good. I thought she was very professional. And, yes, I trusted her with what she was*  *telling me to do.”* |
|  | **Personal**  **attitudes and**  **expectations** | Nothing to lose | *“…it was only ever going to do nothing or improve things…So for me it was just like if you do this and it makes you feel better, that’s awesome. If you do this and it doesn’t make any difference, well, it’s actually still making me get off my arse and do something. So that’s good.”*  *Melissa: “I guess when I signed up, I didn’t have an expectation. I thought, you know, anything’s better than nothing.”*  *Bill: “I was fairly confident that would give at least some benefit.”*  *“I didn’t expect great improvements. All I wanted was to either maintain it, get a little bit better, but not get worse.” Susan: “I was hopeful but I wasn’t unrealistic. So I didn’t expect, I did not expect a miracle.”*  *Kathleen: “I think there’s nothing negative that can happen, even if it didn’t get better, it’s not a negative thing because you tried.”* |
|  |  | Acceptance and  realism | *“There’s always some pain to have a gain [laughs]. So sometimes doing the exercises, yeah, I would find that there’d be some sort of pain…Yes, it hurts, yes, it’s uncomfortable but hopefully it will keep it moving and going and whatever.”*  *Judith: “And so my aim was to…get back to closer to 15 to 20,000 steps a day – which I achieved. So doing the*  *exercises and strengthening the knee, I was able to get back to all of that again…Realistically I knew it was not going to get me back to running.”*  *Lesley: “So the exercises were 1 aspect of it, but the other aspect of it is just understanding my limits, and maybe*  *tapering my expectations a little bit as well.”*  *“You ignore pain, that’s the thing too. So it comes and goes. You treat it, you get an antiinflammatory, you get a massage, do what you can and just keep going.”*  *Lisa: “Oh look, I’ve had it for so long, I just, it’s just part of life. It’s a limiter, but just puts boundaries on things.”*  *Susan: “It’s just there. It’s just part of me now. So, I don’t feel necessarily…oh, I guess, what I feel down about is when I’m with other people and they go for a big long walk, and I say, “I have to sit down, I’ll wait for you here.” So, that’s pretty annoying, but other than that, I just have to accommodate it into my life.”* |
|  |  | Perceived good health | *“…so I would say my general health was very good. If I’m giving myself a rating out of 10, I would say my general health was a 8.”*  *Elizabeth: “I would say it’s certainly above average…I have atrial fibrillation which I manage, and then I’ve got the*  *osteoarthritis…other people will say we can’t believe that you’re 70 and your energy and activity and stuff like that.”*  *“My general health is fine…I mean, like my knee issues is something that’s been there for a long, long time, so I don’t think my general health affected that program at all; and I’m in good health, touch wood.”*  *Kathleen: “My general health is pretty good. I don’t have any major issues.”*  *Patrick: “Again, I think my general health is pretty good. The only thing I’ve got is osteoarthritis in the knees, which I tend to ignore and work around.”* |
|  |  | Accepting responsibility | *“I really just think it was – I really think it was because I wasn’t doing what I needed to do. I wasn’t – it was mainly in the walking and things like that, I just wasn’t doing it.”*  *Catherine: “Virtually I guess what I’m saying, the problem was probably 100% my lack of 100% commitment rather than any fault of the study.”*  *Matilda: “So I think I get – when I say I didn’t benefit from it, it’s more that I probably didn’t adhere to the exercise program well enough to benefit from it. But I think if I did, I probably would. Does that make sense?”*  *Joseph: “I think for me it’s more disappointment for not following it through like I should have followed it through I guess…At the end of the day when I did turn up it was really good.”* |
|  | **Osteoarthritis**  **beliefs** | Exercise is important | *“…It’s the answer. If you can’t strengthen those muscles, you’re not going to see any improvement. Without strengthening those muscles, you’re just going to become a couch potato.”*  *Gregory: “… Increasing the muscle strength helps – I don’t know what the term is but it helps support the knee, helps the function in the knee.”*  *Patricia: “But I’m pretty confident that it was, you know, and it was whatever the damage that I had could only be improved by doing strengthening exercises.”*  *“You just can’t pop a pill for relief. You have to do other things, other logical things like the exercises and strengthen and what have you.”*  *Joseph: “I know exercise is correct. That’s obviously just to strengthen what you have got there and it does work. As far as I don’t know, massage or manipulation or TENS machines, braces and that I don’t know if that makes any difference but I agree with, well, just exercise in general. I know that works.”*  *Matilda: “If you don’t keep your legs stronger – mine aren’t strong enough. I know that, and the more strength you lose in the muscles around – that support your knees, the more limited you become in what your capabilities are and what you can do, and so you lose some. Without the strength in your legs, you lose your life. You lose a desire to go and do things.”* |
|  |  | It’s degenerative | *“…the bones now have become weak at the very end of the leg bones, where they would normally be cushioned on the meniscus, so they’ve become soft. And, yeah, it’s just they’re more tender, that’s my understanding of it.” Lesley: “…there must be a link surely that things have to wear out. Just like your car wears out after a certain amount of kilometres.”*  *Gregory: “The right knee was – worn out, wearing away on the inner side of the joint just because of the structure of my legs so that’s what caused it. My knees are plain old worn out.”*  *“And as I said, they’re stuffed anyway. There’s no cartilage in either knee so there’s only so much you can do. So it’s a maintenance – it’s not an improvement program, it’s a maintenance program.”*  *Catherine: “I just think osteoarthritis is just a part of life. It’s incurable, if that’s the word, and you’ve got to live with it and therefore manage it.”*  *Michelle: “…they’ve worn out. I did 50 years of hairdressing. I’ve done a lot of heavy work in my time like concreting and stuff like that. A lot of gym work which most probably wasn’t real brilliant for them. So I reckon they’re just worn out.”* |
|  |  | Body weight as  contributor | *“I think that was half my problems with my knee is because I’m overweight so that doesn’t help in the first place.” Cynthia: “I think if I lost 20 kilos, which should be my ultimate game, maybe 30, I suspect my knees would improve out of sight. And I mean, I lost 10 kilos and actually after losing 10, my knees did feel a bit better. So if I lost another 20, I probably think they’d be a lot better.”*  *Michelle: “And then I lost – I can’t remember how much it was now, it was like a fair bit in 6 months – like 8 kilos or something – and I think nothing else would – well, apart from my physical fitness would have been pretty poor – so I think those 2 things. If I could have got more weight off more quickly, I reckon I might have seen more benefits, you know?”* |
|  | **Self-efficacy** | Exercise adherence | *“I was very, very diligent with the exercise program certainly through the first 3 months. I didn’t miss a single day. Did all the exercises as required and to the level required.”*  *Judith: “I felt, as I worked on the exercises – like religiously, doing them every second day.”*  *Valerie: “I must admit, towards the end, I did flag off a little bit. Mainly because my knee was feeling so good.”*  *“I followed exactly what I had to do. Yes, 100 percent.”*  *Catherine: “Probably because I didn’t do the exercises. I did some exercises with trepidation for fear of causing my back pain…There was times that I just didn’t do the exercises due to other factors, whatever, at the time.” Joseph: “The first time I went back it was I pretty much did them all. And then the second time I went back I did, I don’t know, three-quarters. And the third time I went back I did half and sort of dwindled away so by the fifth time I went back it was hardly anything.”* |
|  |  | Empowered for selfmanagement | *“But we covered the book and it got me to a stage where I was comfortable doing my exercise and there was nothing of concern really…”*  *James: “Well, now I understand it is very important, understanding that a little bit of pain is OK and how to deal and manage that pain and understand that some pain to do with any sort of physical activity is OK and I’m not doing any further damage…”*  *Melissa: “It got me where I had a wide variety of different exercises that I could do and I felt supported and I knew what I needed to do, so I didn’t really need more [physio consults].”* |
|  | **Barriers** | Comorbid health  conditions | *“I thought it was doing me OK but then no, I just couldn’t deal with it anymore…I ended up having to have injections in my hips afterwards. Because I do have bursitis in my hips, so it actually created more problems for me.”*  *Catherine: “I have had for some years, quite a long time ago diagnosed with depression and I am on medication for that that works but different issues in life that come up are still very hard to live with and I did have a very severe bout of depression, and that happens.”*  *Joyce: “Well I think it was the overriding factor – my general health with my hips and my back and that were the major contributor for me easing up and not doing as much as I should have been.”* |
|  |  | Stressors and life  events | *“I persevered with it until a couple of months ago, because I had a lot of bad news in the family and things – stress just took over.”*  *Michelle: “Kept it up right up until most probably just before I left. And then I was packing. So it was more a time factor, and I was – the packing – I had to do most of it myself. And I was really struggling…So there really was no thought of, “Oh yes, I must do my exercises,” because by the end of the day I could barely move. So yes, I dropped off a fair bit in that few months leading up to moving.”*  *Matilda: “Other things happened in my life that changed as well about the same time. That always complicates outcomes, and they were not something that could be avoided. Just some of my physical activity ceased due to other issues, other people’s injuries, actually. So there was a bit of a sudden change in lifestyle.”* |
|  | **Outcomes** | Strength gains | *“It did in terms of resistance and reps and stuff, yes, it progressed a lot. I got a lot stronger, definitely, yeah.” Judith: “I felt all the muscles leading into my knee really – so you know my quad, my hammy, I felt all of that starting to build strength, which was taking a bit of pressure off the poor old knee as well.”*  *Lesley: “You could just definitely feel the strengthening in the quads and things like that, that were actually taking the load off the knee a little bit.”*  *“I must admit at one stage I did feel better. When I was doing the exercises initially I did feel stronger and more flexible and that side of things.”*  *David: “Well, I do feel that it did – I got stronger in the legs, and that was a help.”*  *Michelle: “I felt so much stronger. I could barely walk, I’d use a walker – inside the house, just to go to the sink. And when I first went there I could barely walk and I was doing just a few hundred steps a day…And then I worked up to 6,000 and – I could walk to the sink without my walker. So I definitely got improvement as far as strength went.”* |
|  |  | Measurement  limitations | *“When I filled out that final part of that survey, I was just recovering from being really, really sick…And yes, but unfortunately that survey asked me how I felt in the last 2 weeks and I wrote on it that I had been very sick so I didn’t think it was really fair to have to write how I felt in that 2 weeks.”*  *Catherine: “But if I’m having to mow a lawn, lift a lawnmower into a car, which I did and ended up with 2 bulging discs in my back, you’re not doing the survey justice at those points in time.”*  *Matilda: “I don’t think it helped with the pain, put it that way, because that’s not – but that wasn’t my issue anyway…And so, my knee issue was more learning to live with the dysfunction and how to avoid falling over, learning how to use my legs differently…So that’s why, I suppose, the questions, when you do a survey, questions are always limited in terms of how you can answer them because of the way they’re worded…they’ve been worded for a particular answer, and you can’t always give that answer.”* |
| **Veenhof C et al. (2006):**  **“Active involvement and long-term goals influence long-term adherence to behavioural graded activity in patients with osteoarthritis: a qualitative study”** | **Exercise adherence** | adherent | *“Although I experience the same level of pain, I have learned to continue with my activities and I realise that I achieve more because of that”* |
|  |  | Non adherent | *“Because my complaints disappeared, I was no longer motivated to continue with the exercises and activities”* |
|  | **Factors relating to exercise adherence** | High satisfaction with intervention | *[none]* |
|  |  | Positive experience with physiotherapist | *[none]* |
|  |  | Former experience with physiotherapist | *[none]* |
|  |  | Positive attitude towards physical activity | *[none]* |
|  |  | High self-efficacy | *[none]* |
|  |  | Social support | *[none]* |
|  |  | Time available | *[none]* |
|  |  | Motivation for treatment: short term | *“I wanted to get rid of the pain. If the pain disappears, why would I bother to continue the exercises? I understand it is better to do the exercises to avoid the pain returning, but, if the pain returns, I will start the exercises again”* |
|  |  | Motivation for treatment: long term | *“I continue with my exercises, they are integrated in my daily living. I really know these exercises have beneficial effects and that motivates me to continue with my exercises. The main motivation to do all this is to prevent an operation to get a new hip”* |
|  |  | Active involvement of patient | *The approach of the physiotherapist was very democratic, which I appreciated. Together, we discussed the activities and the increase of the activities. I could indicate to what extent I wanted to increase the activities, to what extent I could maintain the exercises* |
|  |  | Passive involvement of patient | *The physiotherapist determined the gradual increase of the exercises; he told me, for example, to increase the*  *exercises by five minutes. I liked it that he told me what to do, nevertheless, he was my physiotherapist* |
| **Wallis JA et al. (2020):**  **“Barriers and enablers to uptake of a contemporary guideline-based management program for hip and knee osteoarthritis: A qualitative study”** |  | Program access (barrier) | *“Generally most costs because I’m a pensioner, my wife still working though. I have to consider it. And you come to a certain age where your body is falling apart and I need, for example at the moment, another hearing aid. There are lots of things that I need at the moment. Yes financial considerations do matter.” Patient 4*  *“Well, I think that you must always consider the cost of it, I think that's the first one.” Patient 20*  *“Whether my daughter’s got the time to be taking me to all of this.” Patient 7*  *“Parking around any hospital, not just [Hospital], is a nightmare and you do not want to be in a situation where you have to use the hospital parking because it costs a fortune.” Patient 15*  *“I reckon – quite frankly, I reckon there's a 50/50 chance. And I couldn't commit to anything further than that, because as I said, work is of the utmost – unless, as I said before – unless there's a way in which I can go on sick leave.” Patient 19*  *“So where I lived, it was more than an hour travel to [location] and back and I thought I’d rather spend that hour in the gym.” Patient 8* |
|  |  | Misinformation about osteoarthritis (barrier) | *“And I’ve been told that I’ve got bone on bone and I do need a new knee.” Patient 18*  *“He’s just saying this is a one-way ticket, basically. When it gets bad enough, we’ll give you a new knee.” Patient 1*  *“The cartilage, yeah. So I’ve had three arthroscopies and meniscus tears but along that pathway, it was explained to me that I had very little articular cartilage in both knees, so I was expecting that it’s going to be a problem.” Patient 6*  *“Well, I went to sleep and I’m guessing he put something in and scrape – he’s told me, he scraped a lot of arthritis out because there was a lot of broken stuff in there. That’s what he told me.” Patient 11* |
|  |  | Patient and program factors (barrier) | *“I don't know what the exercises involve, but I would have to consider the impact of those on my back.” Patient 2*  *“You really need to have a strong back to start this because you’re going to be doing exercises and your back has to be strong.” Patient 18*  *“If I was experiencing pain after the sessions or I didn’t feel it was any benefit as the weeks went by, I might consider stopping. Certainly if it was causing pain but that doesn't happen.” Patient 6*  *“I’d like to know how much attention you’d get because you can be doing something, you can attend the program and be doing it wrong.” Patient 18*  *“It’s not so much the time. I think everyone’s got time. It’s just whether mentally you can get yourself in a frame of actually doing it. That's the issue. I think we all got time. You're kidding yourself if you didn’t.” Patient 21*  *“I'm hoping to go overseas in five weeks and I'll be away for three weeks. So, I wouldn't be ready to start before eight weeks.” Patient 2*  *“Well nobody knows about the GLA:D program.” Patient 12* |
|  |  | Health professional trust, feedback and advice (barrier/enabler) | *“Well, if my specialist had recommended it, I would’ve had my decision validated.” Patient 6*  *“I just trust the professionals what they tell me that it needs.” Patient 18*  *“If I’m happy and I have confidence in a physio that I go to, I will trust that they will do the right thing by me as a patient.” Patient 15*  *“You’re lucky if you’d get a quarter of an hour at our physiotherapist, don’t you?” Patient 20*  *“My GP wasn’t keen on surgery, so their attitude was just keep going and try and avoid it at all costs, whilst my approach is different. I’m trying to fix the issue and particularly at the age I am so I prefer to have it rectified and then I’ll be good for another period of time.” Patient 21*  *“I was told I needed some tablets. And then I went down – I saw the specialist and because the knee was such in a poor state, we virtually jumped straight into surgery.” Patient 21*  *“GP? He doesn’t do anything because he just prescribe the medication but nothing else.” Patient 5*  *“It was me that asked the GP if I could come back here. She did not suggest it. She was into anti-inflammatories.” Patient 9*  *“I said is there anything I can do she said, ‘No, nothing,’ and I think that would be the general answer for most GPs.” Patient 1* |
|  |  | Opportunity to achieve positive outcomes and potentially avoid joint replacement surgery (enabler) | *“I’m very keen to start it because I’m looking for anything that would improve my ability to walk, and it’s not surgical, to be honest, if I can get away with it.” Patient 18*  *“I’ve really gone off painkillers, so I rarely take an ibuprofen now and I rarely take anything stronger.” Patient 8*  *“If I felt generally more mobile and a bit better.” Patient 6*  *“He was physically incapable of sitting up and within four weeks of that program, particularly the floor exercise, he can get up and he sits. It was just amazing.” Patient 1*  *“It was only during the education that I discovered that exercise is actually the best way to strengthen the joint which takes away – well, in my case, is taking away all the pain.” Patient 1*  *“Well, I – the thing with surgery, it seems to me, is that once the surgery is taking place, and there's been the particular period of physiotherapy, that there's very little pain, as I have heard. Is that going to be the outcome of this sort of course?” Patient 19* |
|  |  | Better program promotion, patient and health professional education and efficient referral processes (enabler) | *“Once you understand that the program will provide the benefit, then you’ll finish it, but it’s that first one or two sessions and until you’re confident that doing this slightly painful – not necessarily painful, but difficult exercises –- until you’re convinced of that, you won’t complete it. And once people like me that have completed the program, helping the cause to tell other people and other volunteers it’s – that’s the way to get them through. That will give them the encouragement they need.” Patient 1*  *“I think putting a positive spin on it saying that you’re going to improve, so if take part in this program, you’re going to feel better, you’re going to improve, you’re going to walk back, so I think something positive.” Patient 18*  *“It’s a very good thing if you’ve got their GP onside. So, perhaps it’d be good idea to have some group sessions with the GPs. By group sessions, I don’t mean just getting them in a room like this and talking to them. I mean tell them to come along in their exercise togs and actually do it.” Patient 10* |
| **Lawford BJ et al. (2022)**  **“Challenges With Strengthening Exercises for Individuals With Knee Osteoarthritis and Comorbid Obesity: A Qualitative Study With Patients and Physical Therapists”** | Psychological challenges | False assumptions  about exercise | *The improvement it made… I suppose [I was surprised] that the type of exercise I did could make a difference, I*  *wouldn’t have thought the exercises I was doing would make any difference at all this study taught me that exercise can definitely help with mobility, with arthritis and I think a lot of people including myself were frightened of that, thinking “oh no, I’m going to hurt myself. Going to injure myself. Going to wear out my knees”, you know – it was the opposite effect. The more you move, the better it feels, you know?* |
|  |  | Fear of pain | *“I had been afraid to exercise because of the pain, and because of the study, I'm now aware that I can actually do*  *something about it rather than just sit on the couch like I had been doing just being told that it’s okay to feel pain…I think it was finding that it needed to actually be uncomfortable, nobody had ever said that, they just said you need exercises and I’d sort of slightly cheat, I’d sort of do them but not really do them because I didn’t know what it was meant to feel like”* |
|  |  | Disliking exercise | *“I hate exercise. I have to say, I hate it. I'm one of these people that never go to the gym for exercise”*  *“I’m a person if I start the gym, I go swimming and I do it a few times and I stop. I’m lazy, whatever”*  *“I mean, I'm a bit lazy, I don't really like exercise”* |
|  |  | Underestimating  capability | *“[the physical therapist] had, I think, more faith in what I could achieve than what I did first and it was right. I could*  *achieve it because I continued on and trusted him”*  *“I would say that this is a bloody hard exercise and he’d say “well, just get on with it” sort of - I didn’t resent what*  *was going on, it was some parts were difficult.”*  *“the physiotherapist challenged me to up the weight, like rather than just keep the same weight on all the time and do more repetitions it was, actually put greater weight on”* |
|  | Physical challenges | Complexity of WBE  program | *“the only exercise I wasn’t keen on was - because I could never balance myself very well - the step-up… I always felt that that particular exercise, for me, I probably never really did it correctly”*  *“I couldn’t do all of the step-downs, because of physical limitation with that, but I certainly tried to do most of them and with the physio, we varied them as much as I could so that I could, you know, get as much out of it as I could with my physical limitations”* |
|  |  | Cuff weights  problematic in NWBE  program | *“the way that I had to attach the weights to my leg, it was just about impossible to do it by myself …if they were a lot easier to use I probably would’ve kept them up a bit more than what I did, but it was just very awkward”*  *“it’s pretty difficult to manoeuvre when it’s not properly strapped in around your ankle… that was probably the*  *hardest part on me, was preparation…It was the reconfiguration of the equipment that weighed on my mind before I said, “Oh, gee, I’ve got to go do that again. I'm going to blow about an hour””* |
|  |  | Straight leg raise  difficult in NWBE  program | *“I liked them all except the straight leg lift; that was the hardest one and still is the hardest one of them all. Very demanding”*  *“The only one I had trouble with was the lightweight with the straight leg, lifting that up. I had a little bit of trouble - It was something to do with my lower back, where your spine goes down and separates towards your buttock”* |
|  |  | Other health conditions | *“I had a shoulder issue at some point and so I just went and saw about that, and I had bursitis of the hips.”*  *“I get depression, so sometimes I just fall into a big hole and can’t quite function very well. So, we just got through that”*  *“I was really sick for quite a long time and I ended up with pneumonia”*  *“I’ve actually got quite unwell over the years. So, I ended up being in hospital and off work”* |
|  | Overcoming challenges | Incentives to exercise | *“seeing some results early on I think, and then getting keen to do it…That was the main thing, that I got some improvement fairly quickly. Even now, the more I do the better it feels most times.”*  *“When I first started I had to sort of talk myself into it but once I saw results I was able to keep going very consistently.”* |
|  |  | Accountability | *“If it wasn’t for a programme that someone was going to use the results, well, I probably would’ve thrown the towel… some sort of motivation to do it is the biggest thing, which is probably having ongoing contact with a physio or something, maybe, cracking the whip sort of thing”*  *“I could do it with someone's support - but as soon as the study ended I just sort of dribbled off and I stopped*  *doing it - I can’t seem to self-motivate without that outside support”*  *“I was surprised I was committed to it, but part of it was because I felt like I didn’t want to let the program down”* |
|  |  | Education and  reassurance | *“the knowledge that exercise can help. I had no idea that actually exercise could help like that…that knowledge is the big one that really surprised me”*  *“I think reinforcement of the benefit [is important] because I think they probably have enough information now to*  *say, well, if you do stick to it, if you do it the way you’re supposed to do it, the number of times a week you’re supposed to do it, you will see an improvement. But you can’t, if you go at it half-heartedly then you get a half-hearted result.”*  *“I think if it was made clear at outset there was benefits, then I would've probably stuck to it or tried hard to stick to the programme more....I could see it helping but I think a statement at the start would've been helpful.”* |
| **Griesemer I et al. (2020):**  **“Developing a couple typology: A qualitative study of couple dynamics around physical activity”** | **Couple typology** | Working Together Works | *“[PALS] would not be worth a dime without your partner.” And later, “…she’s the motivator… She really helps me out.”*  *“I like to do physical activities with my wife. We’re together most of the time, so most of it is together.”*  *“… my partner was very supportive in my activity and he encouraged me. ‘Let’s go for a walk after dinner’… instead of, ‘Well no let’s stay home and watch TV’.”*  *“No, he did not discourage me one bit. He didn’t do anything to prevent me from being active.”* |
|  |  | Doing Our Own Thing | *“Well, he and I don’t really like many of the same things except for walking…He loves to golf and I picked up golf a little bit but we don’t seem to find the time to do that much together. He definitely doesn’t like Zumba or water aerobics and he doesn’t like swimming at all. Really the only thing we share in common is the walking.”*  *“He would remind me, every day, to go exercise, and everything, and I always kind of took it the wrong way, that he was saying exercise because you need to lose weight. To hear him say, ‘Don’t forget. You need to exercise today.’ To me, I heard, ‘You are overweight. You need to lose weight. That is what I heard. Which is probably my fault. Well, it is.”*  *“I prefer separately. Only because my partner tends to want to tell me what to do. I want to do it at my speed, and how I do it…and not be told what to do, like a child.”* |
|  |  |  | *“He’s more active…He walks faster. He’ll slow down for me, so usually if he walks with me and does stuff for me, that’s really not his routine. He does it with me just to be with me because it makes him happy to see me get out and do some stuff. If he does it with me, it’s more a side thing for him versus a routine.”*  *“It’s a lot easier to exercise by myself. Frequently, since we’re both retired, and we both work part time, frequently*  *our schedules don’t match. I end up exercising by myself. When we are at the house together and I’m ready to exercise, more than likely when I say I’m going for a walk he will join me, but, like I said, many times our schedules don’t jibe.”* |
|  |  | Different Realities | *“It’s hard to be motivated for two. If she was more motivated, that would be awesome,”*  *“When you’ve got somebody there to encourage you and say, ‘Come on, let’s get it done,’ you know? It helps a lot. Every day you might not feel like getting out of that door, but my wife will say, ‘Come on, get them bones loose, let’s go.’ We stayed at it, and we stay at it.”*  *“I’ve always asked her if she thought that this exercise we were doing would work for both of us, or did she need to do something else. She would think of different exercises we could do, and I’d participate with her. I’d think of stuff we could do, and she’ll participate with me.”* |
| **Ledingham et al. (2020):**  **“Exercise adherence: beliefs of adults with knee osteoarthritis over 2 years”** | Monitoring | Group exercise | *“I liked the camaraderie, hearin’ from other people who were experiencing similar problems…it gave me the motivation to also keep doin’ what I was doin’ to stay active.”*  *“I could see where I was, as opposed to where other people were. And I could see how we were coming along, as the program progressed. Who seemed to be doing much better than when they first started, and then it [observing other group members] gives you incentive to keep on with what you have to do.”*  *“Well, some people would, say…“I got fifteen pounds of weight on my [leg],” and I said, “Oh my gosh, how do they do it? I can’t even do four.” [laughs] “And they can do that many?” I said, “Oh, my, maybe I can do that too, eventually”*  *“Well, it’s a commitment, and if you don’t show up, then the group notices.”*  *“I knew exactly what the expectation was in terms of getting it [the exercise] done correctly and for safety’s sake. I certainly didn’t want to be injured. So just being here with you guys watching closely…themovements that I was making, correcting them when [they] needed to be corrected, constantly – it made a big difference, 'cause to go home and not be sure exactly how to do…the steps or the movements for safety’s sake, would’ve been a cause for concern, I would be like, ‘Am I doing this right?' You know, and that in itself for me is stressful. I don’t want stress.”*  *“I really liked the structure of having to show up and go through the exercises”.* |
|  |  | Telephone technology | *“Knowing that I’m gonna get that phone call!.”*  *“Even thought I don’t like it, [automated telephone calls] [laughs] it’s a good motivator.”*  *“I mean, I think that’s something incredibly important,I would love you to keep calling me. And I hate them, I hate the um, the what-do-you-call-it voice. I hate the idea of it [laughs], I mean I, because I [laughs], I hate that the whole thing was happening to us all the time… the automated voices [laughs]. But I really appreciate it.”*  *“It [BOOST-TLC] would ask you more specific questions, how many times did you exercise, what were your goals, and I thought that was good. It was kind of a pain in the neck sometimes [laughs]. I said “Oh, I don’t feel like doing this,” but I thought it was good, because it made me think, “OK. When am I gonna exercise?.”*  *“…it [the telephone calls] shows that the BOOST Program cared about you. And they wanted to make sure you do your exercise, make sure you was, followin’ the protocol, it was very beautiful.”*  *“At the beginning, it’s very encouraging, but after a while, it’s kind of, to me it’s kind of tedious.”*  *“But for me, the problem was that I went through a transition. I went through a transition from work, from workin’ in the office, to workin’ at home, and I’m on the phone, and on the computer, and I’m always on the phone all day for, for almost, for pretty much eight hours. So at the end of the day, I’m talked out. I don’t wanna be bothered [with the BOOST-TLC].”* |
|  | Knowledge of exercise | Self-management | *“it’s the one step at a time. You do not have to…race at a certain pace. You do it as much as you can.”*  *“…while we were in the group each person was able to proceed and progress as they were physically able and mentally able.”*  *“…if you didn’t have time to do all of ‘em, you could just start some of them during the day, just as long as you finished it during the day, and I know I would get tired, and I said, ‘Oh no. I forgot to do the lunges,’ you know, that was the last thing, OK. But you know, if I started out in the morning [leaving her home], I was doing them everywhere… I would go quilting, and… I see the stairway [to perform step-up exercises].”* |
|  | Perception of exercise benefits |  | *“…[I] could do more work in the house, and OK, maybe go out and take my grandson, to the park by myself. No need to wait for my husband. If my leg is not strong enough, I have to wait for my husband, ‘cause I’m afraid that – he’s [grandson] running around, [I] won’t be able to catch him.”* |
|  | Beliefs about adherence at 2 years | Low adherence | *“I think the other reason I probably fell down a little bit on doing the work, the exercises, was that I think after a point I wasn’t convinced that even though I knew strengthening would help, I wasn’t convinced that it would allow me to change my lifestyle back to what it used to be.”*  *“Because it makes a difference. It’s more motivating when you’re doing it [exercise] with someone else. It’s easy to go back to your regular routine of doing nothing. I mean, if it’s just me, I don’t, I don’t care about me. [laughs] But it’s different, I’d care about someone else.”*  *“…a suggestion might be to have like a reunion… Do the exercises, …I guess, hearing people’s experiences with the exercises, seeing if we were doing them right, tweaking them, you know, getting some feedback.”*  *“I missed the group. Maybe sometimes we could have, during the interim, have one group study again, come in and see how everybody’s doing, and then go back. So in between the phone calls, every now and then there’s… I don’t know, quarterly or whatever, just come in that one time. See who’s havin’ any difficulties, and how you can change that.”* |
|  |  | High adherence | *“if you want something bad enough you do it”.*  *“…if you had any problem, you had a takehome manual, if you go through that, that would help you…if you forgot the procedure, you know, the right way to stand – or whatever –”*  *“the instructor went through everything with us, and it was like, it was uh, yeah, that’s a challenge I have to do this, I have to do this. I got to do this to try to overcome some of the pains and get some of these stiff joints movin’, you know. Because I had committed myself to ‘em, [exercise] and I had got into a pattern that I was doing them, and I did them.”*  *“It was an easy process for me, because I had everything laid out. I kept my bag right there in the kitchen, so while I’m in the kitchen, before I start my day, I would just do my exercises.”*  *“…and it seems like everything I did here [during the exercise class], I was able to do at home with no problems.”*  *“found it a little bit intimidating at first [the exercise class], ‘cause it was like an obstacle course, where you had to do this, then you went from that to a [another] thing… I’m saying, “Oh gosh, I can’t do this. This is a bit much.” But then I said, “But no. Let me give it a shot and just try to do it,” and it’s not like I gotta be vigorous with it, just take my time, go at it”* |
| **Östlind E et al. (2022):**  **“Experiences of activity monitoring and perceptions of digital support among working individuals with hip and knee osteoarthritis - a focus group study”** | **A WAT may aid in optimization of PA, but is not a panacea** |  | *“...it will be easy to push or trigger yourself to go those steps extra if you are at 6,500, it is easy to motivate and take another walk to reach the goal.”*  *“I’m amazed at how controlled I am by it, 7,000 steps, it was like, that’s what I walked every day. And now that I don’t have this [the WAT] anymore, I don’t think I take that many steps anymore. I’m really affected by it.”*  *“It’s positive that it beeps when you haven’t walked 250 steps in an hour. When it “beeps” you get to move and take a turn in the corridors at work…”* |
|  |  | Increased awareness of one’s limitations | *“- And that you learn the relationship with how you feel. - Yes, exactly. - The leg or the knee or the hip or whatever it is... That you learn how many steps I must walk so that it does not hurt.”*  *“We had it for so long that I felt that at 8,000 [steps] it started to get too tough afterwards, so I tried to stick to it, and I thought it worked well. Then there was never any [pain]... So previously I activated myself a lot and then nothing... It became a much better rhythm.”*  *“I could see [in the app] that I should probably quit now. Plus, you can say it to others: I have taken the steps that*  *I can manage, and I can’t tag along any longer.* |
|  |  | WATs are not always encouraging | *“It is not a purpose in itself to have a digital app, you must be spurred by it as well. So just putting on a Fitbit does not help if you are not interested.”*  *“So, unfortunately, it had no effect because I [my hip] is so terribly bad and consequently, I could not walk as much as I would like.*  *“You get disquiet if you do not reach 7,000 steps… I think it happened to me one day and that was very tough…”*  *“You can go too far with this, as you said, you push yourself and then you have to do a little more and then you have to do a little more and you will never be satisfied.”* |
|  | **Digital support is an appreciated part of OA care** | Individualized, early, and continuous support | *“I feel that this… I participated in the SOASP… that it was me and then it was 90-year-olds.”*  *“It [SOASP] should be sort of more separated in the age groups maybe because I have no one... but it felt like they were not in the same stage as I was. I would probably like to have that.”*  *“Early, yes. So that you can come to this realization about losing weight and that you need to train certain things or so. Otherwise, it’s just; ‘Well, now I have a little worse mobility, now I lean forward…’ ”*  *“.. you have a mentor or a physiotherapist that you meet every three months or when necessary to update your exercises, steps, and the Fitbit. Help with that…Find the level, get that support. – Kind of like a diabetes nurse. -Yes, it might be like that.—OA physiotherapist.* |
|  |  | PT is essential but needs to be modernized | *“That’s what I miss about Joint Academy (digital platform). I have never shown how I do my exercises. So theoretically, I can do them completely wrong.”*  *“Someone could help me check what it is that makes me feel so bad today, if it’s because I did too much or I did too little or what could be the cause... Then I was grateful because I can’t find a pattern myself and don’t really know...”*  *“It can be a good discussion basis for the follow-up visit: “You have walked far too much” or “you have not moved enough.””* |
|  |  | Digital support should be easy, comprehensive, and reliable | *“I think it’s a problem, that you can’t get in… That I can’t make it work. I feel it’s like a sort of handicap. But once it works, it’s amazing.”*  *“… someone must probably instruct me what to do and how to set it up because as I said, I’m not interested in sitting and looking among the apps and what features they have and so on...”*  *“I would like to have an increased support so that you get the whole concept of diet and other things as well, it would have been great, I think.”*  *“It was very often that I looked at [the WAT] and... Oh, ok, so now I have cycled for twenty minutes at a high pace, and I received no credit for it. It’s annoying.”* |
| **Leese et al. (2021):**  **“Experiences of Wearable Technology by Persons with Knee Osteoarthritis Participating in a Physical Activity Counseling Intervention: Qualitative Study Using a Relational Ethics Lens”** |  | Making Choices About Physical Activity With or  Without a Wearable | *1_Hazel: the Fitbit…It’s like a little person on my wrist…it’s a little friend…and I tap into it and go, “I’m almost to my 10,000 but I could do a bit more”…throughout my day I can refer to it and I think it’s on my side. It’s happy when I do 10,000 steps…just like a friend supporting me, encouraging me…whether I achieve it or not it’s still there with me…Whether I do it or not, it’s up to me. There’s a gentle persuasion…a Fitbit helps me feel less*  *alone…it’s huge for me because you know most of my life I’ve not had a lot of support and so support is huge, just huge.*  *2_Sansa: when I go on there and I see that I haven't reached my six or whatever, I'll in the evening go for you know a five-minute walk or a 10-minute walk or you know try to do that after dinner. So it does give me a little bit more kind of accountability or kind of check where I'm at. I think it's kind of like I have a partner in crime, it's just kind of there to keep me accountable.*  *3_Martha: It probably gives me some incentive to walk a little further just to placate the Fitbit…I forget to check but I think it does give you an incentive to get out and do something because it’s there and it nags you…I’m fine with that…it’s probably a good thing to have something that makes you get up and go.*  *4_Denny: I finally get home say around seven in the evening…just kind of want to eat and then just do nothing…I know you’re supposed to move [laughs]…But sometimes I’m just too tired and in the evenings, I’m forced to kind of do some more activities…having the Fitbit it does make me feel I need to move more…I have definitely gone for more walks.*  *5_Joe: I max out in my activities, so I don’t need this monitoring as a way of positive feedback or gratification to give me incentive. I personally don’t need that…I’m actually walking and all of that. If somebody says, “Oh you should dance more in the evening,” I say, “Well no I can’t dance anymore.” I can only go so far then I drop dead right? So I’m maxed out. I can’t add much more here…So when this six months is over, I’ll take the Fitbit and*  *throw it away because it has no relevance to my life…I know what I’m doing and I don’t care what this little machine tells me.* |
|  |  | Emotional Dimensions of Adding Awareness About  Physical Activity | *1_Daenerys: I will always keep the Fitbit and always have one I think because it lets me feel as though I’m accomplishing something every day, like I have it set to a pretty low number of steps every day. It’s set to 3000 but when I look at my results I can be over 3000. When it goes off during the day, I feel pretty happy about, “Okay, I’ve accomplished that much today,” and then it’s kind of fun to see how much more I can do.*  *2_Logan Kale: when I would be close to my steps, if I would see…you just tap it [the Fitbit] and then you’ll know if you’re close or not and I would just make that extra effort to meet that mark. Like instead of driving to work, I would walk to work. On most days it was easy…just seeing that number of how you’re so close. “Got to get over that hump.” Once a goal you’ve set and, you know, when you reach that goal, you feel good about it. It’s just*  *happiness, accomplishment. The other days I’m just in too much pain. I’m like, “I’m not walking home.” I want to be that person going on those hikes. I don’t want to be that person just sitting there. You want to always try to do better the next week but then if it doesn’t happen, I try not to beat myself up over it anymore because, you know, the next day could be better. I’m like, “Okay well you didn’t do well this week. What’s the problem? You shouldn’t be doing that. You shouldn’t be doing this. You should be doing that,” and I just kind of get stuck on that hamster wheel of negative thoughts and have to zip it.*  *3_Biker: [responding to you mentioned that there were some goals that you didn’t meet…] I don’t feel bad at all. [Laughs] I just kind of go, “Oh that’s life” because I know that I’m keeping really active so yeah it’s not a problem to me. I guess if I thought, “Gee I’m not very active and I’m not meeting any of my goals” then I might feel sad about it but because I know that I like to exercise and so being active is not an issue for me. So the goals I set are kind of like…in a perfect world this is what I would like to do but the world isn’t perfect and it’s okay. You know, I’m working, I’m certainly getting tons of weekly exercise in and so if I don’t meet some aspect of it, it’ll be okay. You know also I guess I can also look at it and kind of go, “If I really, really wanted to do Yoga, I could put a DVD on and do some at home” but again, I like the Yoga for the social aspect so that’s not much of an incentive to do it on my own in my living room…actually I kind of find it a kind of cozy feeling thinking, “I haven’t completed everything. There’s still more to do.” So I’m not taking it kind of like I’m a failure, I’m taking it more as, “Oh there’s still more to do and you can keep on growing, keep on improving” so.* |
|  |  | Reviewing Wearable Data With the Study PT: Issues  of Accountability and Trust | *1_Denny:…you have a knowledgeable person telling you, you’re doing the right thing type thing…I tried a little bit harder [laughs] maybe for a few weeks…because you actually have another person kind of monitoring you and you also…you want to try…it was very, very encouraging…I think it’s really good for her to be able to see and for me to know that somebody is monitoring me. I think maybe that makes me [laughs] take a few more steps maybe.*  *2_Darius: It’s just a little bit of pressure of keeping the, the steps they...Because I, I need to watch to keep my promise, you know, as far as I can…she [the physiotherapist] called me like every two weeks. I think her purpose is to motivate me for keeping my, my promise to keep activities.*  *3_Gavin: I do have a grump with the Fitbit over the times where it’s gone into sleep mode so many times…Before I know it I’ve lost 3000 steps…you go like, “I get a lot more steps today than that’s showing me. I know it’s slipped into the sleep mode activity and it’s not”…I figured I should have my entire European boot badge by now so it’s not fair…I knew I was getting a phone call from the physio and I said, “Oh yeah but it wasn’t my fault I didn’t make my 10,000. This stupid band didn’t log on properly.” Oh she said, “Oh yeah, it happens.”*  *4_Gavin: I think [Fitbit data] at least gave a better conversational point in terms of seeing how you were doing, a reference check for the physio when we’re checking in. If it wasn’t there it would really…there would be nothing for her to gauge because then, “What did you do?” “Oh yeah I climbed Mt. Everest this weekend and am feeling really good. Kilimanjaro is tomorrow. Not bad.”… we would’ve been talking in a fairy land because I could’ve been making up anything about that…”* |
| **Nelligan RK et al. (2020):**  **“Exploring Attitudes and Experiences of People With Knee Osteoarthritis Toward a Self-Directed eHealth Intervention to Support Exercise: Qualitative Study”** | Technology Easy to Use and Follow | Website ease of use | *I’m not the smartest computer user in the world, but if I can do it, I reckon anybody can do it. [George]* |
|  |  | Sms ease of use | *“It's just simple” [Olivia] Sometimes with the SMS, I'd put the letters and the things around the wrong way… it was very particular, you know, you had to do it in the right…But other than that, no worries at all. [Lucy]* |
|  | Facilitators to Exercise Participation | Credible OA and exercise information | *[none]* |
|  |  | Website features | *“More than what the doctor has given you” [Emily].* |
|  |  | Prescribed exercises simple to do unsupervised | *An allied health (person) to actually monitor the exercises was not necessary. [Harry]* |
|  |  | Freedom to adapt the exercise to  suit needs | *[none]* |
|  |  | Influence of other healthcare experiences | *[none]* |
|  | Sense of Support and Accountability | SMS good reminder and prompt | *[none]* |
|  |  | Accountable | *You felt like you had to do it because you were going to get checked up… [Chloe]*  *It was like a devil sitting on my shoulder going “have you done your exercises?” Oh, my God, I can only put two in for an answer this week; I’ve got to do better next week. [Sophie]* |
|  |  | SMS tone and automation could trigger negative emotions (eg, guilt/shame) | *“it was a reminder of the bleeding obvious” [Amelia]*  *When I got the planter fasciitis and the texts were coming through…they just kept coming, and it was kind of like a little shame thing. [Charlotte]* |
|  |  | Inability to contact someone when needed | *[none]* |
|  | Positive Outcomes | Knee symptom improvements | *[none]* |
|  |  | Confidence to self-manage | *…it’s helped me not only with my mobility but my self-confidence to be able to go, yeah, I can get up there all right and come down there. [Grace]*  *Not only has it changed the strength in my knees and reduced the amount of ongoing pain that I have with them, it’s also inspired me to lose weight. I’ve actually lost at this stage — about 14 and a half kilos. [George]* |
|  |  | Encouraged active living | *[none]* |
|  | Suggestions for Real-World Application | Provided by a health professional preferred | *[none]* |
|  |  | Should be provided at subsidized or low out-of-pocket cost | *“so long as it wasn’t too expensive” [Grace]* |
| **Petursdottir U et al. (2010): “Facilitators and barriers to exercising among people with osteoarthritis: a phenomenological study”** | Internal Factors—Individual Attributes | Motivation | *“I have always enjoyed physical activity” (Audrey)*  *“I did it because I knew it was good for me, but not because I liked it” (Ellen)*  *“It is dead boring, so I just don’t do it and never will” (Mary)* |
|  |  | Personality | *“I worked out new ways to cope, to keep my arthritis from getting in the way too much” (Betty)*  *“I think that general positivism is part of your health; if you think constantly about pain and aches, then you get really sick” (Indy)* |
|  |  | Self-image | *“I was extremely unhappy with myself… I couldn’t work as hard as before, and I just could not understand why. It was one of the hardest things, to accept myself as what I had become” (Audrey)*  *“Well, you have to face the fact that you are not young anymore, and you just have to slow down” (Hans)* |
|  |  | Health and exercise attitude | *“I cannot let the arthritis overtake you… I was not going to let the arthritis stop me” (Audrey)* |
|  |  | Exercise history | *“I played sports when I was young, but then I quit. I never had any endurance, so I was never good at it” (Mary)* |
|  |  | Disease knowledge | *“Now I think I handle it more wisely. I know better because I’ve been fortunate to get good instruction” (Audrey)*  *“There are many 60-year-olds who don’t use computers to get information. And these are the people with arthritis! I think it is much easier to get information to the younger people. We use the Internet” (Audrey)* |
|  | Internal Factors—Personal Experience | Effect of pain | *“I know that when I’m done I feel better. That’s what I’m constantly after” (Nancy)* |
|  |  | Effect of stiffness and fatigue | *“It was like my body was made of lead” (Kirsten)*  *“The effort to get clean afterward is really hard… You just don’t have the energy to take a shower” (Kirsten)* |
|  |  | Finding suitable exercise | *“I think that physical therapists are the best to help those who have a physical dilemma to start exercising… and start carefully, and under supervision. I think that is very important” (Audrey)*  *“And I think that it is important when people choose which exercises to do, that you enjoy it, that you feel it is rewarding… these positive factors have to be present” (Audrey)* |
|  |  | Perceived benefits of exercising | *“Exercising has a good effect on everything, including the heart” (Carl)*  *“I am more vivacious, both physically and mentally” (Carl)*  *“I feel it right away if I gain a pound; I feel it in my hips and knees” (Laura)*  *“Well, if you don’t move, you get fat, no matter how little you eat” (Gerda)* |
|  |  | Quality of sleep | *“Then you get lazy [because of a bad night’s sleep]” (Carl)*  *“All activity is good for sleep” (Audrey)* |
|  | External Factors—Social Environment | Family support | *“Yes, my wife, naturally, she encourages me” (Carl)*  *“It [the experience of lack of support] was, just, what should I say, totally pathetic… I guess men are not all equally understanding” (Audrey)* |
|  |  | Physical therapists’ professional care | *“Well, I always say that my physical therapist is as good as any psychologist” (Gerda)* |
|  |  | Physicians’ encouragement | *“He encourages me in every way” (Carl)*  *“They have not done it [encouraged exercising]” (Gerda)*  *“They [the physicians] are positive if you ask [for a referral to a physical therapist], but you have to ask” (Gerda)* |
|  |  | Training partners | *“I like exercising in a group the most… I’m more reluctant to go alone into the gym” (Gerda)*  *“I like being free when it comes to training time and just decide for myself when I do it and when I don’t” (Betty)* |
|  |  | Socioeconomic status | *“And this costs money. Walking, however, is free. Such things matter when you only have your pension” (Carl)* |
|  | External Factors—Physical Environment | Effect of weather | *“And this costs money. Walking, however, is free. Such things matter when you only have your pension” (Carl)* |
|  |  | Availability of exercise classes | *“The problem was that I never found any that suited me” (Kirsten)*  *“Well, there was this note on the wall saying the aqua-exercise classes are about to start… But for whom?” (Audrey)* |
|  |  | Accessibility of facilities | *“If I was a boy or a man, I would kick those machines; I hate adjusting them, it takes half the time” (Audrey)*  *“Walking upstairs is the worst thing for me” (Gerda)* |
|  |  | Transportation | *“But now I’ve decided to quit driving” (Kirsten)* |
|  | Exercise Behavior | Self-directed exercise | *“The swimming pools are what I would recommend to every person with OA” (Nancy)* |
|  |  | Individual physical therapy | *“What keeps me going now is attending physical therapy sessions” (Gerda)* |
|  |  | Group exercise | *“I think it is the best exercise class I’ve ever attended” (Audrey)* |
|  |  | No exercising | *“There is nothing that can be done about the OA; therefore, I do nothing” (Mary)* |
| **Battista S et al. (2021):**  **“Giving an account of patients' experience: A qualitative study on the care process of hip and knee osteoarthritis”** | Understanding the facilitators of the adherence to therapeutic exercise | Importance of being active | *“…The body has to move…” (P3, female, 73)*  *“And, this kind of movement (walking), I realised, was good for me” (P2, female, 68)* |
|  |  | Perceived exercises as concrete support to the cure | *“Then the situation improved, always with training, thanks to the workouts” (P7, female, 45)*  *“It [physical exercise] is not like taking supplements with hyaluronic acid, those (supplements) you do not see what they do” (P1, male, 49)* |
|  |  | Mean to maintain functionality | *“OA is difficult to cure, even impossible, it is a natural tear, only some palliatives exist. I believe that the only way, or rather, the best way is to strengthen the muscle structure so that bones and joints suffer less from the weight load on them” (P11, male, 56)*  *“I felt well, because I kept on walking… and this allowed for reducing OA impact’. (P10, male, 65)* |
|  |  | Willingness to change life‐habits | *“…Determination and willpower [to change life‐habits]” (P7, female, 45)*  *“A great willpower is necessary [to change life‐habits]” (P9, female, 73)* |
|  | Understanding the barriers to the adherence to therapeutic exercise | Cost and lack of time | *“Yes… but also from an economic point of view [it is difficult to do supervised exercises]” (P2, female, 68)*  *“We are trapped into a spiral in which work, we can say, takes up a lot of energy and a lot of time, and then that time is taken away from us…” (P6, male, 55)* |
|  |  | Lack of clear indications | *“That is, there were some, just some things [decisions in the care process]… Erm… I don't know… they were left to our intuition, to our perception but just because you understand that by acting in a certain way, maybe you will limit its progress [of OA]…” (P2, female, 68)*  *“The doctor told me: “You know that if I did not know that these x‐rays belong to you, I would think that they belong to another person who is at least 30 years older than you”… but, I guess I did not feel as bad as he was describing me” (P11, male, 65)* |
|  |  | Lack of willpower and fatigue in changing life habits | *“So it is that maybe when you are old, people back down, they lie on the couch… Surely such a pain affecting someone who does not have that drive [motivation to stay fit] makes people unwilling to get up from the couch” (P1, male, 49)*  *“I think so, for laziness. Because if you want to, you are able to find the time. So it is, therefore, laziness” (P5, female, 72)* |
|  |  | Exercise perceived useful only after surgery | *“But I imagine that someone can do this… let's call it preventive activity. Activity that can help with the recovery process following the intervention” (P6, male, 55)*  *“It is useless to start doing physiotherapy/exercise if I am undertaking surgery in a month” (P9, female, 73)* |
| **Stone RC and Baker J. (2015)**  **“Painful Choices: A Qualitative Exploration of Facilitators and Barriers to Active Lifestyles Among Adults With Osteoarthritis”** | Barriers | Pain | *“I’m always in pain and agony, every movement is a chore. Sometimes, I just stare at my stairs, dreading what comes next” (P2)*  *“I can’t bend down. I can’t get on the floor, if I do, it is a chore for me to get up. Bending my knees hurts all the time. Walking now seems to be hurting me as well” (P5)*  *“If someone called to play ball or something I would say, “I’m busy, I can’t,” and pretty soon I realized that I couldn’t do it, not that I didn’t want to, I just couldn’t anymore. It wasn’t worth the pain” (P7)*  *“Not only does it hurt when you [move], but it would hurt the next day. The pain never lets you forget . . . and believe me, I don’t. The only thing I can do is not do it again. Avoid exercise, avoid the pain” (P11)* |
|  |  | Psychological Distress | *“Mentally, it’s so depressing. I can’t tell you how I just want to sit down and cry sometimes because I can’t move” (P13)*  *“It definitely wears on you, on your mind because it stops you from doing what you want to do. Even if my body wanted to [exercise], my mind won’t let me… I feel helpless and worthless” (P5)* |
|  |  | Lack of medical support | *“My doctor told me to go on a [recumbent] bicycle for 20 minutes a day, or whatever was easiest for me. So she tells me to pick up my hands [to the sky], which I can’t do because of arthritis in my back. So then she tells me to pick up my legs or do sit ups… But I can’t do those either! I’m so confused. I just find it easier to do nothing” (P3)*  *“I was never prescribed exercise. My family doctor and rheumatologist have never even mentioned it. If my doctors don’t think it’s important, why should I?” (P1)*  *“When I was first diagnosed, I didn’t know what to think. I knew it wasn’t good, but I didn’t know how bad it was going to be. After a couple of years, the pain was too much to bear and I thought, that’s it… my life is over. And no one warned me… I didn’t even know what to do… exercise was the farthest thing from my mind.” (P6)* |
|  | Facilitators | Pain relief | *“Sometimes, after a long day, I’ll throw some ice on my knees, take a hot bath after. It feels great… Using the heat was my favorite part of my physiotherapy… That is probably the only way I could handle exercising” (P5)*  *“The physiotherapist professionally guided me to feel less pain. It made me want to do exercises on my own” (P2)* |
|  |  | Medical support | *“If my doctor tells me to [exercise], then I will” (P1, P4, P5, P6, P7, P8, P10, P11, P12)*  *“If [my doctor] told me what to do and how to do it, I’d be at the gym right now” (P6).* |
|  |  | Social support | *“One of my friends who knows about my arthritis asked me if I ever exercise. “Exercise?!” I said, “What could I do with exercise?!” Then she said she would work out with me if I wanted to. That was the first time I ever seriously thought about exercising” (P7)*  *“I know [my husband] supports being active, but I never really felt overly supported until my youngest asked if she could come with me on walks. The shocking interest in my exercise habits motivated me to want to exercise more, and be a great role model” (P13)*  *“I walked into one of the community centers near my house, and was shocked to see on a bulletin board, they had exercise programs running for people with arthritis, and I just felt, “Hey, I belong here!” (P6)* |
| **Al-Khlaifat L et al. (2020):**  **“Perceptions and performance of exercise in people with knee osteoarthritis in the Middle East: are they different to countries in the developed world? A qualitative study in Jordan”** | Individual level | Knowledge of the role of exercise in knee OA | *“Any part that moves would feel better and energized” (2)*  *“Exercises are good for my knees; they are better than taking chemicals” (10)*  *“I don’t know if exercises could prevent worsening of knees” (8)*  *“I don’t know if exercises would improve my knees” (9)*  *“I feel much better but I am afraid the pain will be back again once my sessions finish” (6)*  *“I do not think exercises could worsen my condition” (9)* |
|  |  | Personal factors | *“I have too much work to do, at the end of the day I do not have energy to do the exercises” (8)*  *“When I feel better I stop exercising but when the pain increases I register for new sessions” (9)*  *“I was advised to walk but if my knees hurt, I would stop walking” (14)*  *“It is good for our age if we learned how to swim but it is hard to learn from our children and we are afraid of water” (7)* |
|  | Sociocultural level | Cultural attitudes and beliefs | *“My husband would not allow me to exercise in a gym” (13)*  *“I like to walk, but I do not because of housework, the children, and winter timing” (14)* |
|  |  | Social interaction | *“I would exercise more if there were group sessions, something social, a pool, something that would not be expensive” (1)* |
|  | Organizational and political level | Service delivery process – at the physician’s clinic | *“The Dr. wrote a referral in my last appointment after I have asked for one. I want to try physiotherapy since it helped my other knee” (14)* |
|  |  | Service delivery process – at the physiotherapy department | *“No, she only showed them to me once and quickly” (6)*  *“I do not know, for instance my leg cannot raise fully. Is that what is required from me??” (8)*  *“The physiotherapist would set up the machine and tell me to exercise then leave” (11)* |
|  |  | Delivery of home exercises | *“Only at the end of the last session the physiotherapist told me about the exercises and to do them at home” (9)*  *“They do not ask if you exercised at home” (1)*  *“I exercise in the gym using the machine to bend and straighten my legs with weights and sometimes I use the stationary bike. At home, I would pull my foot up (dorsiflexion) and straighten my leg without weights” (10)* |
|  |  | d. Accessibility of services affects exercise adherence | *“I would like to go to the gym but I am afraid they would not know what I need for my knee. I would prefer to use the gym at the hospital with a physiotherapist” (10)*  *“I would like to go to the gym but there is not one near my home and my husband would not allow me” (14)* |
|  |  | Opportunities for improving service delivery | *“They should focus more on exercises. It takes me five hours to get to the hospital and wait for my turn to just have ice packs” (8)*  *“I would exercise more if I see that I am actually improving” (12)*  *“I would prefer to use the gym at the hospital with a therapist” (10)* |
| **Moody J et al. (2012):**  **“Perceptions of a water-based exercise programme to improve physical function and falls risk in older adults with lower extremity osteoarthritis: barriers, motivators and sustainability.”** | Wonderful | The social part of it | *“the social side of things is really, really good” (FG1)*  *“all in the same boat” (FG1)*  *“Yes, I think the instructor was sort of aware of our capabilities and kept the challenge up. And it made it more interesting that way, because if you did the same thing over and over at the same level, it would be boring.” (FG2)* |
|  |  | It woke me up and got me going | *“Well I thought it was marvellous really it um you know got us out of bed in the morning and got us into the pool and umm the instructor we had was very, very good and ah I think it was just so good. And I think the motivation was there which is the big thing is to get you motivated you know?” (FG1)*  *“Yeah, so it’s just funny little things that keep you thinking you have a responsibility to attend ‘cause someone’s gonna miss you.” (FG1)*  *“…sometimes being actually on your own to be motivated uh it’s harder. It’s harder: A lot harder.” (FG1)* |
|  |  | It’s better to go there than going to the doctor | *“...exercise in the water, it’s not like walking or running…You’re not jarring any limbs or bones…And for old people I’m, I’m sure that’s the best sort of exercise that you could do.”(FG1)*  *“Anything that will help me continue with it? You put it on and I will be there! Let’s start tomorrow!” (FG1)* |
|  | Sustainability | I’ve got one complaint and it is only really my complaint | *“I’ve got one complaint and it is only really my complaint. It was that most of them could manage so much quicker than me.” (FG4)*  *“Umm, I suppose the things that sort of do prevent you are if you get ill. One thing, that’s probably the only thing would be if I got ill…I probably wouldn’t be able to go, but only that would keep me away.” (FG1)*  *“Well for me, at first that’s why I missed some of them. I couldn’t go more than one because I was just so tired the next day and would sleep so sound, you know at the night-time, that I couldn’t always wake up early enough to get myself organized to get the bus.”(FG4)* |
|  |  | We don’t want much do we? | *“Being with the group of elderly people…of same age and we related to so many things that we did, you know. We talked about what helped us and what didn’t help us, you know?” (FG2)* |
|  |  | I’d sooner have a leader | *“No, I’d sooner have a leader.” (FG3)*  *“…couldn’t probably afford a big amount, twice a week” (FG5)* |
| **Hinman RS et al. (2016):**  **“Physical Therapists, Telephone Coaches, and Patients With Knee Osteoarthritis: Qualitative Study About Working Together to Promote Exercise Adherence”** | Genuine interest and collaboration |  | *“The fact that they were genuinely, or seemed genuinely, interested and were monitoring your progress, you know what I mean, and you sort of go back to your health coach, for example, and you feel a little bit proud that you’ve achieved what you, what’s been set for you, to achieve, and similarly with the physio [physical therapist], you know, I’d say ‘I’ve done it’ with a big smile on my face.” (Tom)*  *“I thought, for me, it was beneficial to have both. Initially, I thought no, one’s enough, I don’t need this health coach, but I think putting the 2 together, I think it was beneficial, and it was good. They sort of complemented each other in different ways. Yeah, like I said, one was a pure business-type person, and the other was a very personal person, so they did complement each other, and it worked for me.” (John)*  *“It was just hopeless. She rings up for a chat, and I don’t think she was bossy enough, or clear enough about ‘Now we’re going to have a conversation, and this is what we have to achieve out of this session.’ It was a chat.” (Sally)*  *“With the knee, I suppose I was imagining there’d be more a hands-on assessment of my knee, and it was just really ‘How are you going with the exercises?’ ‘Are you doing them?’ ‘Aren’t you, and how can you do it so it’s not as painful?’” (Mary)* |
|  | Information and accountability |  | *“There were techniques for how to motivate yourself to do the exercises, and that was good, that was very good, because as I said, knowing that someone’s assessing you, I suppose, makes you more, made me more, responsible for doing the exercises.” (Mary)*  *“I know now it’s going to be for my benefit. I keep on doing these exercises… if I stop, pain comes on again, and I can’t do any activities.” (Peter)*  *“The most important thing is listening to the physio [physical therapist] and doing the exercises because he motivated me to do the exercises. It was for my benefit, right, so he kept on pushing me, ‘You have to do it, gradual buildup, don’t go at once, start slowly, work yourself up through the stage,’ and that advice motivated me to do it.” (Peter)*  *“I was a bit skeptical at first and when the exercises came I thought ‘hang on, this has got nothing to do with the knee as far as I understand’ being ignorant, you know. Now I sort of feel that ‘hang on, yeah there is a difference’ because the work has paid off, the pain is different pain and I feel that I can do things easier now than I could a while back so I think there’s been a benefit.” (John)* |
|  | Program structure |  | *“They (the exercises) were good because you could just work your way up, you know, and make them harder. They weren’t so difficult that you couldn’t do them and you didn’t want to do them.” (Lisa)*  *“It’s part of life, it’s what I do. I get up in the morning, I have a cup of coffee, I take my blood pressure medication, then I go and do my exercises, and then I come back and have breakfast, and that’s just become a routine, which in a way is no different from people going to the gym 3 times a week or doing anything else, so it’s my way of doing things. And I don’t have to leave home!” (Peter)*  *“Yeah, it probably is just a bit boring.” (Lisa)*  *“It was boring. Every day, every other day, when you do the same thing, it’s very hard to get motivated, it was a bit boring. Some of the exercises were OK, but some of the exercises… just thought of throwing in the towel virtually, but then I thought the pain versus this, and then it will balance everything out.” (Peter)* |
|  | Roles and communication in teamwork |  | *“I think, for me, it was 2 separate. The physio [physical therapist] was concentrating and getting me better virtually, and there’s she (the coach) asking how I am managing, and, in a way, yet it goes hand in glove.” (Peter)*  *“One was giving me pain, the other was trying to make it easier for me.” (John)*  *“The coaching was very pleasant, very nice conversations in the evening. The physio [physical therapist] was possibly a bit business-minded or a bit focused on the work.” (John)*  *“Yeah, the roles were separate, and my physio [physical therapist] never suggested that he was communicating with the health coach, and I’m not sure, did he or not?” (Tom)*  *“I don’t think they communicated. I don’t think the physio [physical therapist] and the coach knew what they were each doing.” (Sally)* |
| **Moore AJ et al. (2020):**  **“Therapeutic alliance facilitates adherence to physiotherapy-led exercise and physical activity for older adults with knee pain: a longitudinal qualitative study”** | Mutual Investment | Equity in the work/reciprocity | *“The thing is you do the exercise ‘cause you feel that you don’t want to let the other person down. You know you do them ‘cause in the first instance you think, ‘Oh that’s going to do me good, it’s going to yeah’, but also there’s a secondary thing there you think, ‘Oh he’s gone out of his way to explain these things to me and shown me what to do it’s only fair that I do them so at least I can tell him what sort of effect its having the next time I meet him’, you know.” (UC 7058 FU)*  *“At end of treatment was partially adherent to exercises and was an active hill walker. At follow-up no longer did the exercises from the trial but was an active hill walker and had joined a gym.” (UC 7058)* |
|  |  | Appreciative of other | *“So I, I think, and I think it’s because of [physiotherapist] in a way, I didn’t, don’t feel as though I wanted to let him down ‘cause he’d been so good, got me so far.” (TEA 3657 PI)*  *“I also think that because they see you trying, it motivates them as well.” (ITE 1481 FU)*  *“At end of treatment was adherent to exercise and joined a gym. At follow-up was partially adherent, joined different gym and cycled.” (TEA 3657)*  *“At end of treatment was adherent to exercises, used exercise bike. At follow-up was not adherent to exercises from the trial but did other sitting exercises, cycled and walked.” (ITE 1481)* |
|  |  | Joint motivations Negotiation/agreement | *“Watched me doing all the exercise, then she’d write on the list how many times she wanted me to do, you know. But I said to her, I said, ‘Well I’m doing them once, once a day.’ She said, ‘Well as long as you do thoroughly, but do the ten times of each exercise, you know.’ “(UC 6153 PI)*  *“At end of treatment was adherent to exercises, withdrawn from study at follow-up.” (UC 6153)* |
|  | Personal interactions/affective bond | Feeling at ease/relaxed/valued | *“I mean, he always had time to talk to you, and say, you know, ‘Any questions or anything?’ He didn’t rush you in and rush you out or, like, you know, it does happen sometimes but, with people, but, no, he was very good.” (ITE 61 FU)*  *“At end of treatment adherent to exercises from the trial and active through dancing. At follow-up continued to dance but not doing exercises from the trial as knee worsened and awaiting knee replacement.” (ITE 61)* |
|  |  | Perception of therapist (eg, good, nice) | *“She was a nice young lass, you know, bedside manner, the fact that she straightaway sort of suspected what it was, do you know what I mean?” (TEA 30 FU)*  *“At end of treatment was partially adherent to exercises from the trial, cycled, walked dog and did morning stretches. At follow-up continued to cycle, and walk and did exercises from the trial 2 or 3 times a week.” (TEA 30)* |
|  |  | Getting to know each other/making connections | *“We were talking about gardening whatever as I was doing my exercises as well. And she sort of mentioned things about her life and what she could do with various bits and pieces and it just made it a much more enjoyable experience I think. [.] I think it made a difference. It made me feel I wanted to do the exercises more.” (ITE 26 FU)*  *“At end of treatment partially adherent to exercises from the trial. At follow-up partially adherent as tried to do some exercises but others were too painful (due to Baker’s cyst).” (ITE 26)*  *“She’d had a car crash. She herself, her leg’s badly damaged. So she had got an insight into sort of what it was all about, you know.” (TEA 30 FU)*  *“At end of treatment partially adherent to exercises from the trial, cycled, walked dog and did morning stretches. At follow-up continued to cycle, and walk and did exercises from the trial 2 or 3 times a week.” (TEA 30)* |
|  |  | Attentiveness to other | *“I think she was far more realistic and she seemed more interested in me as a person and what I did. I know the other girl talked about it but it was more as a matter of course, not out of . made you feel particularly valuable I suppose.” (ITE 26 PI)*  *“At end of treatment partially adherent to exercises from the trial. At follow-up partially adherent as tried to do some exercises but others were too painful (due to Baker’s cyst).” (ITE 26)* |
|  | Communication | Openness, honesty and trust | *“I do think it was probably seeing that (trial) physio that really made me open up and think this is a shared thing, he is trying to help me. I wanted to be helped. But he was on such a level that I could share things that I’d maybe found in the past difficult to share.” (UC 1135 FU)*  *“Adherent both at end of the trial intervention and at follow-up. Continued with the exercises because felt left knee was deteriorating.” (UC 1135)*  *“Of course she said, ‘Three times a day’ and I’d look at her. ‘How many times you do that?’ I said ‘Once.’ [..] Oh yes, I was honest with her, yes. I said, ‘Once but very thorough.’ [yes], you know.” (UC 6153 PI)*  *“At end of treatment was adherent to exercises, withdrawn from study at follow-up.” (UC 6153)*  *“And then you start asking more... you develop a bit of a rapport and I think that from the study I’m sure the physio gets a little bit more out of it because you start expanding on, on what you’re saying.” (ITE 1481 PI)*  *“At end of treatment was adherent to exercises, used exercise bike. At follow-up was not adherent to exercises from the trial but did other sitting exercises, cycled and walked.” (ITE 1481)* |
|  |  | Listening | *“So I think, I, I was quite impressed with the physio in that she listened and understood what I was saying with regard to both the pain in the knee and my mental health problems and the hernia.” (TEA 7880 PI)*  *“At end of treatment partially adherent and did mountain biking and walking. At follow-up partially adherent, did exercises from the trial but irregularly.” (TEA 7880)* |
|  |  | Explanations offered and understood (exercise self-efficacy) | *“She explained even though the exercises might cause pain, as said, she sort of suggested that the, the problem amongst other things was the lack of strength in the muscle. So she said by building the muscles up that will support the knee better [yeah] in the long run.” (TEA 7880 PI)*  *“At end of treatment partially adherent and did mountain biking and walking. At follow-up partially adherent, did exercises from the trial but irregularly.” (7880 TEA)*  *“By going seeing someone every week for a period of time, I think you, you develop some trust, some openness comes from the... from my part, comes from that as well, some understanding.” (ITE 1481 PI)*  *“At end of treatment was adherent to exercises, used exercise bike. At follow-up was not adherent to exercises from the trial but did other sitting exercises, cycled and walked.” (ITE 1481)* |
|  |  | Reassurance/confidence (exercise self-efficacy) | *“Reassure me. Reassure me. Give me the right exercises to do, if it wasn’t going to do any further damage, if it was arthritis. I think the cartilage problem is caused by, or could have been caused by the arthritis. I needed reassurance that it was okay to actually do the exercises and I wasn’t going to cause further damage. Confidence I think because sometimes trying to do these things on your own is a bit scary if you get stuck, because my leg does lock. And I think probably him encouraging me to do the right exercises, and do them every day, which I did do.” (UC 1135 PI)*  *“Adherent both at end of the trial intervention and at follow-up. Continued with the exercises because felt left knee was deteriorating.” (UC 1135)*  *“I’ll be a lot more confident, I think I’ll be a bit, say, worried, but at the moment, I’ve got sort of motivation that I’m being seen sort of every month. Um, just reassurance really, I suppose isn’t it. Um, I’m doing my exercises and I’m going and um, the physio’s going through the exercises with me and telling me this is – well I know it’s improved, but it’s just a bit of reassurance and – and motivation to keep going a bit longer [mmm] with them.” (ITE 6878 PI)*  *“At end of treatment was adherent to exercises from the trial, declined interview at follow-up.” (ITE 6878)* |
| **Campbell R et al. (2001):**  **“Why don't patients do their exercises? Understanding non-compliance with physiotherapy in patients with osteoarthritis of the knee”** | Initial Compliance |  | *Geoffrey: “Well I felt because [physiotherapist] took the trouble of explaining it all to me I couldn’t turn around and say, “well blow it, why bother sort of thing? you know?” And when I first turned round and said that I would do it, I felt well alright I wasn’t obligated to do it but I felt let’s do my bit towards it, you know. I didn’t want her to simply think that she was wasting her time.”*  *Kenneth: “I’m prepared to pile in and help out sort of thing, if that’s what you want ’cause it helps both ways . . . . . . . . .I think it was worthwhile and if anything has come out in the form of knowledge or expertise, call it what you will, for any of the people I have seen [the physiotherapist and the researcher running the trial] then fine, I’m all for it.”*  *Graham: “I felt that I was contributing in some ways to research which would probably benefit other people, and that really was why I went ahead with it ...I anticipated some benefit for myself, but I thought well, this is great if this is going on, then I am participating in something really worthwhile.”*  *Alan: I went along, my own attitude in the back of my mind was, I don’t think it’s going to do me a lot of good, but the way I look at it was, sometime in the future, what they learn from here, might possibly benefit somebody else . . .So I did it more for the sake of the research than anything else”*  *Prue: “I am not a one for taking a lot of tablets. I get a bit dubious, you know, so I just learnt to live with it for a bit and then I had the chance of [the trial].”*  *June: “I’m a great believer in physiotherapy anyway I think. I don’t agree with drugs quite as much as, I think, if you can have it naturally.”* |
|  | Continued Compliance | Attitudes towards exercise | *Alan: “It wasn’t so much at home I am able to do it, it’s more at work . . .. Perhaps not as often as I would really like to, but I can do it quite freely then, because I’m totally on my own.”*  *Kenneth: “So many things happening ... The boys used to come in from school or work… people come and see [wife] and ugh . . . I’m out twice at least a week to band practice and I have two engagements as well.”*  *Eileen: “Weekends I try to do [the exercises] but I am very busy on the weekend really it is theonly chance I get to do sort of any cleaning. Then my family usually come up in the afternoon, my sister and her husband, because on the way to Mum’s they always come in. Sunday’s I have Mum on Sunday see, then I have her two sisters because they are older.”*  *Kenneth: “It’s just excuses when it comes down to basics. I mean you know you could get up in the morning and do it between 6 or 7 or something like that.”*  *ME: “So would you say that some weeks you do them [the exercises] two or three times?” Eileen: “Some weeks I can’t . . ..” ME: “Sometimes you can’t make it at all?” Eileen: Yeah. Latterly. I think this is my own fault. I mean when I was going [to see the physiotherapist] every week you make yourself sort of do it don’t you . . ...I must admit I am not so good now I am not going”*  *Stanley: “If perhaps my wife would work with me and you had a bit of competition, but I feel such a fool standing on one leg and going up and down on my own and I tends to drop it I do. I’m not very strong disciplined on that, no. I know some people can be so, but not me. I suppose if there was a really good reason I would.”* |
|  |  | Perceived severity of knee symptoms | *June: “It got worse and worse and I started falling down ... Since I started strengthening these muscles it seems I don’t fall over so much which is good ... it’s so embarrassing.”*  *Bridie: “When I did the exercises in the beginning, it wasn’t painful with the tape on, so I think that was how I was able to get on with them so well . . . whereas if the tape came off and I didn’t put it on it was more painful.”*  *Beryl: “I see people come in with arthritis and I think oh... god they are in terrible trouble and absolute agony and I think well I’ve got nothing to complain about.”*  *Geoffrey: “There was a time when I missed one [session with the physiotherapist]. I don’t know why . . . . . .I think it was taking the wife some where I don’t know and I must honestly admit that her needs come as a priority as far as I am concerned.”* |
|  |  | Ideas about the cause of arthritis | *Ethel: “[the exercise and taping]might not help me because I’m getting old but it might help somebody else ...I just think I’m too old really to improve.” Geoffrey: “I was having trouble with my knees every so often it did hurt you know with one thing and another. Working in the construction industry there is a lot of lifting and a lot kneeling you see and I felt well I wonder if that’s got anything to do with it. So I go to the doctor and all he just simply done was put his hand on my knee, he said “move your leg, . . . you are getting old you’ve got rheumatism.” You see that was it I didn’t take any more notice of it [the knee pain]…” MT “Since you have stopped seeing [the physiotherapist] have you stopped doing the exercises?” Geoffrey: “Yes I’m sorry I have yes. But as I said I haven’t had no pain . . .. I wondered whether it was temperature or dampness or something like that you see. Now there is nothing wrong with them.” MT: “So you feel if there is nothing wrong with it you feel there is not much point in a...” Geoffrey: “Well that’s it. It’s the wrong attitude I know.” Geoffrey: “as I told him [one of the doctors running the trial] really I feel a bit guilty taking his time up because there must be a lot of people a lot worse than what I am.”*  *Vi: “Because when you’ve got knees like this, you like to do other things, you think I’m gonna go—I’d like to get back to how I was before, but I don’t think that’s ever going to happen now. I’m sure the weight is the biggest problem . . . . . .I don’t eat as much as I use to, nowhere near and I was slim then. But I love me food so.”*  *June: “I think there is a lot to be learnt and a lot to be done for [arthritis] because even a simple thing like plastering [taping], that is cheap, quick and easy, isn’t it?”* |
|  |  | The perceived effectiveness of the intervention | *Bridie: “I still do [the exercises] and I remember to stand the correct way without even thinking about it now . . . . . .. [The pain] has been a lot better, much better, and I can do things better. Dressing—I don’t have to hold on to anything, I can balance now and in fact, you know, I find it a great improvement.”*  *Beryl: “She [the physiotherapist] said the kneecap is out, so she taped it up and pushed it back . . . So now if it starts aching, that’s what I do. I tape it up and push it back to where it should be.”*  *Arthur: I was able to do [the exercises] pretty easily but it didn’t appear to me to make a lot of di Verence . . . I carried them on during the time I was taking part in the programme although I’ve dropped them since.*  *Vi: “I found that when I didn’t have the tape on I missed it. But I don’t know whether that was psychological or—but I found it helps because when I was walking down the stairs, it was supporting—you know what I mean? But the only trouble with that was, I found that by using it quite so often I used to get a reaction [to the sticking plaster] on my knees, it was sore.”* |
| **Bell et al. (2024): “I feel more confident”: a mixed methods evaluation of**  **the influence of Good Life with osteoarthritis Denmark**  **(GLA:D) on physical activity participation, capability,**  **barriers, and facilitators in people with knee**  **osteoarthritis** | Theme 1: fear of knee joint damage, and scarcity of exercise and physical activity information for osteoarthritis prior to GLA:D | Participants report fear of pain and damage to the knee | *“I wasn’t attempting any exercise on my legs. I wasn’t even going there because I was just too worried about incurring more damage.” - P8*  *“I was cautious. My knee hurt, it was pretty stiff, and I was incapacitated but I was now used to it.” – P18*  *“I was a bit hesitant and reluctant too because, of course, my pain I was worried, I’d cause myself an injury.” - P12* |
|  |  | Previous experience and beliefs about physical activity reduces willingness to participate in physical activity | *“I had no problem with doing any exercise and it I was sort of physically active anyway that [GLA:D] was just an add-on to what I was already doing.” - P10*  *“I was always keen on exercise and physical activity. I just thought I would have to greatly reduce it because of my knees.” - P17*  *“I felt like I wanted to do it but couldn’t. So I felt limited by my body rather than attitude. I felt a high degree of frustration with my inability to do things that I wanted to do.” - P6*  *“I wouldn’t even have considered the daily exercise routine.” - P5* |
|  |  | Health professional education rarely included information about exercise-therapy | *“I had very little from GPs [general practitioner] or any other professionals. I’d only had a GP do couple of scans or x-rays... and that’s it. No treatment, no exercises, no referrals.” - P10*  *“I probably haven’t received any education [about exercises]. I mean the only thing the surgeon ever said to me was, “I’ll see you when you’re ready.” - P14*  *“The knee surgeon I saw said... you can take a few pain killers, but that’s not gonna get rid all of the pain totally...Get a new knee.” - P18*  *“I mean I went to see a knee surgeon and he was the one that suggested I did the GLA:D program.” - P4*  *“I’d seen physios over the years and got fairly general advice. But I didn’t know it was general advice until I started doing [GLA:D].” - P2*  *“One other physio who’d said to me, “You cannot exercise if it hurts.” - P1*  *“I was already going to the chiro and he recommended a physio.” - P9* |
|  |  | Uncertainty of quality of online information and bias towards surgical interventio | *“I was looking for information about knee replacements. So, I didn’t find a lot about exercise. However, on one Australian site ... there was stuff about general exercise being good to keep especially if you suffer from osteoarthritis, to keep the joints sort of moving. But when I was looking at that stage, I was getting pain in my knee at times... I wasn’t taking notice of the exercise suggestions. And there was nothing specific was like walking, swimming, bike riding... Nothing with dosages, nothing to help explain...the problem is a lot of it is American stuff and it seems to be connected to a place or sponsored by people trying to get you to have knee replacements...” - P2*  *“I think I always remember what reading about and bringing up on the surgery I suppose, yeah, a little bit. I can’t remember [exercise] stuff from the website.” - P17*  *“Online is very confusing. There’s too much information.... Then you work out whether you trust what they say.” - P18* |
|  | Theme 2: varied exercise-therapy and physical activity participation following GLA:D | Engagement in varied exercise therapy following GLA:D | *“I’ve gone back to the gym and hopefully talking to the physiologist so I can incorporate some of GLA:D into it.” - P3 “And in doing the GLAD program, it made me more aware of what I might be doing in my Pilates and Barre classes and RPM [cycling] classes and things like that...” - P19*  *“I was sort of physically active anyway that it was just an add-on to what I was already doing.” - P10*  *“... going for walks with my husband, the dogs, on the weekend. I can’t walk too fast. But now, we will walk a long way. I was also going back to the gym doing circuit classes, doing the weights.” - P13* |
|  |  | Varied physical activity participation following GLA:D | *“I’ve gone back to the gym and hopefully talking to the physiologist so I can incorporate some of GLA:D into it.” - P3*  *“And in doing the GLAD program, it made me more aware of what I might be doing in my Pilates and Barre classes and RPM [cycling] classes and things like that...” - P19*  *“I was sort of physically active anyway that it was just an add-on to what I was already doing.” - P10*  *“... going for walks with my husband, the dogs, on the weekend. I can’t walk too fast. But now, we will walk a long way. I was also going back to the gym doing circuit classes, doing the weights.” - P13* |
|  | Theme 3: physical activity participation facilitators following GLA:D included reduced fear of knee damage, increased  confidence, routine, strategies and support | Changes in knowledge and  beliefs about physical  activity following GLA:D | *“Made me realise I have to be more active and more specifically active... do things that are going to help strengthen the knee as well as improve my cardio activity... The knowledge I gained from the program... a) that may not fix things anyway and b) I can actually strengthen the knee... Knowledge most important thing is knowledge and the fact that I was doing exercise regularly because of the sessions, which I wasn’t doing without the sessions...” - P2*  *“I think it showed me why what happens in your knee with the theoretical part of and then with the exercise program developing the “why” after learning about it in theory, the “why” and how it’s going to affect your knee and how it’s going to strengthen it and the benefits follow.” P10*  *“I think it was just realising that the more you move, the better off you’re going to be, long term.” - P3* |
|  |  | Reduced fear of knee pain  and damage and  improved confidence  with exercise-therapy  and physical activity | *“I just feel fitter and stronger and more confident in everything I do.” - P12*  *“Certainly, the fear of the pain has changed. I don’t have that anymore. I’m more confident of what I can do with it now, without damaging it.” - P2*  *“If we continue on with that sort of exercise regime there is no need for surgery.” - P7*  *“And that confidence has made a hell of a difference because it’s not just someone telling me, it’s I’ve experienced how I can actually learn to strengthen it.” - P2*  *“Yes, the program did help me. The benefits are mobility, more confidence in trusting my leg, and as I said before, the understanding that exercise is not to be shied away from.” - P18*  *“I feel more confident, especially going up again the stairs... it has given me the extra confidence to just try things... I’m much more confident to doing more walking now.” - P3*  *“It gave me more confidence to go to some of the other classes at the gym that I wouldn’t have gone to, I thought there’s no way I’ll be able to go to those because I can’t squat or I can’t lunge. Where now I have been known to them, so that’s really improved my confidence to go to those other classes.” - P13*  *“...I was told I have to stop running... I suppose [I’ve got] confidence in getting back into running.” - P17* |
|  |  | Routine, strategies and support facilitate opportunities to remain physically  active | *“I need to do it early in the morning... It just made me more regimented in what I do and get back to exercise.” - P5*  *“I’m gonna be happy to pay to do another 12 sessions just to try and create that habit. I think it’s definitely stuff that I can do at home... I did like that they tailor it specifically for me.” - P15*  *“Maybe a little nudgy every now and again... Something like a booster session or a follow-up session or a check-in and are you still doing things right kind of thing would be good... Maybe twice a year.” - P6*  *“...I’m going to have to make that time and book it in. So I’m committed. It’s that accountability stuff.” - P14*  *“It’s up to the individual to apply yourself.” - P11*  *“... So anything I can do in order to keep myself mobile is going to work for me.” - P14* |
|  |  | Impact of external supports  (including technology,  subsidised activities and  social interaction) on  physical activity  participation | *“I mean I’ve got access to the videos too if I get a little bit stuck.” - P3*  *“I guess the ease of having a physio, supervising in your own home that’s a big positive for me, if you’re timepoor. I guess having it evaluated is important too, there was a 30 percent improvement in that getting up out of a chair- that’s significant.” - P14*  *“A phone application...” - P3*  *“A little text to remind you every now and again doesn’t hurt.” P6 “Yeah, being able to track [physical activity].” - P18*  *“...if the government, if it could be subsidised, the money is saved in knee replacements and lack of productivity, and people being on disability pensions and all of those things would be enormous...” - P13*  *“...There’s also a social aspect to it so then we go and have a cup of coffee after and I think that’s quite nice.” - P1* |
|  | Theme 4: physical activity participation barriers following GLA:D included persistent knee pain, comorbidities, cost, lack of opportunity, and lack of motivation | Impact of ongoing knee pain and comorbidities on physical activity participation | *“...still just knee pain.” - P7*  *“... The inflammatory conditions that I’ve had with hyperthyroid, the whole package of things that had slowed me down tremendously over the last three or four years...hopefully if my hip settles down...That’s the limiting factor with that now, not my knees...” - P6*  *“Pain and discomfort.” - P4* |
|  |  | Lack of motivation as a barrier to ongoing physical activity | *“I’ve never been a person just to do specific exercises like that on my own. I’m a shocker when it comes to self-motivation.” - P1*  *“[The barrier is] basically my motivation my starting, my doing something. There’s no physical barrier as such.” - P2* |
|  |  | Impact of cost of exercise therapy and physical activity programs on opportunity to participate | *“I guess what would hold a lot of people back is the cost. A lot of people just don’t see that as an important thing. I mean they might spend money going out to the pictures every week but a lot of people have a lot of trouble spending on their health, whether it would be buying good healthy food or preventative things and so you try and educate people that, yes, it might cost $500 for the program but the benefits you’ll get.”...” - P13*  *”there’s got to be some sort of other way that they could package it because, otherwise, that will be too financially... if I was paying for it, I would say, I can’t do that.” - P8* |
|  |  | Impact of social and physical environment on opportunity for physical activity participation | *“...There’s a whole lot of stuff going on at work, which makes it really difficult ...” - P14*  *“...You get a bit busy doing other things and you don’t actually do specific physical exercise, and because I do nana duty, I stopped playing tennis.” - P5*  *“It just hasn’t occurred yet and with Christmas, it’s chaotic at the moment.” - P5*  *“I used to do a lot of bike riding, rode thousands of kilometre and whatnot but, unfortunately, the reason I gave that up more to do with safety and more accidents riding a bicycle than I have in my life and that’s disappointing...” - P1 “...I’m troubled by things like weather and that sort of thing.” - P6* |
| **Lawford et al. (2024): Understanding Negative and Positive Feelings About Telerehabilitation in People With Chronic Knee Pain: A Mixed-Methods Study** | Easier than expected |  | *“Never having done care remotely before, I was unsure as to the effectiveness, but it worked well.” Male, 56 years*  *“Initially I was wondering, would they way we were doing this consultation be successful as I had always done face to face. How would I manage to get onto Zoom? How can my Physio show me without me being there? Well, I had no problems at all, the exercise book and straps was a great help and my Physio had no problems in making sure visually that I was doing my exercised the right way. I was very happy with the overall experience and would do it this way all the time if it were available.” Female, 74 years*  *“I was interested, but couldn’t see how it would work. I was very surprised how well it worked. I put that down to the skill of the physio.” Male, 62 years* |
|  | Surprised by the benefits/effectiveness |  | *“I was pleasantly surprised by the benefits I received” Male, 57 years*  *“I’m not sure why but I didn’t really see video conferencing as a conventional method of physiotherapy and was not sure how it would work. I was pleasantly surprised how well it works.” Female, 66 years*  *“I was not sure how it was going to work as I have always had hands on physio. But the experience was a positive one and achieved similar results to a face to face appointment.” Female, 74 years* |
|  | Strong rapport with physiotherapist |  | *“Surprised at the rapport between patient and physio even though it was via a computer screen! As a new user of Zoom, it was just as good as face-to-face.” Female, 64 years*  *“video conferencing can feel a bit odd but [name] and I were able to quickly build a rapport.” Female, 67 years*  *“I felt comfortable with talking to the Physio and built a good rapport with him. It was easier to perform the tasks so that he could see what I was doing.” Female, 70 years* |
|  | Convenient way to attend consultations |  | *“It was so easy to attend the appointments, and be at home.” Female, 50 years*  *“I had a really positive experience and found this way of attending appointments very convenient.” Female, 57 years*  *“It has to do with the time factor for me - being very busy with work and family often makes me question if I have time for these things, but being online meant I didn’t have to take time from work or family. The care was still of a high standard.” Female, 62 years* |
|  | Prefer hands-on treatment |  | *“Feel it’s [physiotherapy] a hands on treatment.” Female, 57 years*  *“Still prefer hands on.” Female, 67 years*  *“Not entirely sure a hands-off approach is the right thing for all situations.” Female, 58 years*  *“I feel physiotherapy is more about physical assessment - touch, sensation, pain. Also appreciate face to face guidance and the use of massage etc.” Female, 56 years* |
|  | Does not allow adequate assessment or observation |  | *“Physiotherapy is a practical form of medical treatment. It is not just sitting in front of a camera, talking. The physiotherapist needs to be able to see the affected area, check if there is swelling, etc. It is also difficult to see exercises properly and to have to change the camera angle constantly as you stand up to do an exercise, sit down to talk, stand up, sit down, move around the room. I considered it a waste of time and would not participate in the future.” Female, 55 years*  *“I want to make sure am doing the exercises right.” Female, 64 years*  *“I just don’t think you can really assess if exercises are being performed correctly via zoom - the calls are good for background information and general information/teaching but I don’t think you can get the precision element required.” Female, 54 years* |
|  | Prefer in-person contact |  | *“Would prefer to see someone face to face to gain motivation.” Female, 61 years*  *“I would prefer to see the physio in person.” Female, 68 years*  *“Video conferencing was adequate and useful but not as good as in-person. I consider it is not quite as effective and less value for money. It was essential during Covid restrictions and definitely better than no sessions.” Male, 60 years* |
|  | Just do not like telerehabilitation |  | *Just doesn’t do it for me.” Male, 81 years*  *“Because I just don’t like it.” Female, 61 years*  *“I don’t like video conferencing.” Female, 66 years* |
| **Allison et al. (2023): ‘I saw it as a marriage—You can't have one without the other’: A qualitative study of patient and physiotherapist experiences with a therapeutic combined strength and aerobic physical activity exercise programme for hip osteoarthritis** | Positive outcomes | Functional improvements | *Brad: ‘The guys I play with, they've really noticed, oh, geez, you're hitting the ball a bit further, which is, you know, because I've played golf 30 years and not, I'm not going to improve, but I have.’*  *Bill: ‘Now I can put my foot, I can cross my legs—I could never do that before. Or won't say never, in the last 12 months I'd have pain doing that. And you know I put my sock on quite easily. And cut my toenails, all those sorts of things so there's just, even just the mobility improvement is worth it.’*  *Hannah: ‘It's quite what I needed, and also I found that I wasn't able to… I wasn't able to vacuum and mop the floors in the same—timeframe, so it's a case of mop or vacuum 1 day and not another day. But now I'm back to being able to do it all in one go and that starts the side movement as well.’*  *Martin: ‘I've pretty much gone from, if I'm sitting in my van for work and I've got to drive for an hour, getting out and feeling stiff, to not having that at all.’* |
|  |  | Pain relief | *Sue: ‘I just don't want to be in pain and not being able to do the things I want to do. And so, this has shown that it’s helped.’*  *Bill: ‘Since I started on the programme things like aching in the early hours of the morning, after a few weeks of on the programme I haven't had any aches at all and my hip got to the point where I didn't even know it was there 99% of the time.’*  *Gail: ‘I used to feel like bit of an old lady getting out of bed, but that's really improved. I think that's improved for a couple of reasons because I'm not in so much pain but also I just feel better because I'm not in pain, if that makes sense.* |
|  |  | Empowerment through experience | *Hannah: ‘I've learnt a lot about my body and what I can do. I've learnt to watch for trigger signs and what do I do when that happens. So all in all, it has been a positive experience, but a lot of having to adjust to what I felt was working for me.’*  *Louisa: ‘I'm in a more positive state than I was 3 months ago, and I feel I can do more. I feel stronger and my family have noted changes in the things that I've been able to do.’*  *Mary: ‘I started to go back to the gym, like, I went back to aqua aerobics, I went back to Zumba, because I felt more confident, and I think that the confidence was a big factor, before this I was losing confidence, I was losing confidence on the public transport, I was really thinking how am I going to get around walking when I travel, and that was worrying me, but when I went to Auckland, because it's a hilly town, I was now very confident.’*  *David: ‘But if I find that my symptoms are coming back I will take from that the understanding that perhaps I need to be strengthening something else and doing one of the other exercises to strengthen a different part of the muscle, et cetera. And I think I understand enough about that now to do that myself.’* |
|  |  | Commitment to continue | *Sue: ‘I know I'm committed to keeping it going for 12 more weeks now, and I'm hoping that after that the pattern's made…… I've got a lot of reasons to keep it going from a lifestyle point of view.’*  *David: ‘I've already had the discussion with the physio about how it is that I continue the exercises whilst I'm away.’* |
|  | Combined benefits of aerobic and strength exercise | Complimentary effects | *David: ‘I think strength and aerobic exercise types are inseparable. I don't think one without the other would work. I mean you need the strengthening exercise to build you up and then you need the aerobic stuff to make sure that it’s working, and frankly you need one for the other.’*  *Celina: ‘I think you need the combination of both strength and aerobic, because the increase in your walking increases your—what's the word I'm looking for, to be able to keep doing the exercises. I guess your strength I suppose, and then to combine it with the strength stuff, I think they're both combined, you've got to do them both, that's my feeling.’*  *Mary: ‘I saw (the strength and aerobic exercise) as a marriage. You couldn't have one without the other or you'd end up wonky.’*  *Gail: ‘I suppose maybe I felt a bit overwhelmed by the combination of strength and aerobic exercise—and that's why I couldn't commit as much to the strengthening as I wanted to—and that's why you come up with all these reasons why you can't do it. So perhaps that was a little bit overwhelming.’* |
|  |  | Strengthening exercises key | *Hannah: ‘If I'm going to improve, strengthening is the core… the reason that I'm going to improve.’*  *Claire: ‘I had been previously walking anyway, and wasn't really getting any results, then we added strength and I was getting results.’*  *Bill: ‘All the exercises through the programme—I thought they really worked. All the strength exercises, in particular. I think it's probably the strength exercise that made the improvement. I was walking a bit and because I changed and with now doing these strength‐ building exercises I have to attribute to that to being the best, or the most effective of the lot.* |
|  | Valuing support from a physiotherapist | Personalised care | *Gail: ‘Definitely what worked for my body was the exercises when the physio and I found the ones that were not aggravating pain for me.’*  *Mary: ‘I'd done a previous programme, and this was much better. I suppose it was because it was individualised, not in a big group, tailored to your specific needs rather than a generalised programme rolled out for people from severely disabled to moderate to mild, and also much older people.’* |
|  |  | Skill performance feedback | *Jenny: ‘It's the physical seeing someone that reminds you no, you've not got the angle right or you're not holding enough.’ Louisa: ‘I liked being there because he could look at my technique. I don't know, it's just a bit personable.’*  *Gail: ‘A lot of the time when I'd go see Damien, we'd just try and correct my technique. That was important because something as little as getting my foot placement back in the bridge exercise, that was what he was looking for. So refining the technique was important.* |
|  |  | Coach effect | *Bill: ‘(The physiotherapist) was providing the encouragement to me as well. So it was okay that I wanted to achieve something and I could see that—but having someone else recognise it and give you credit for it is invaluable as well.’*  *Louisa: ‘It was very helpful having a physio. He just sort of pushed me along a bit, whereas I think if I was doing it at home I’d probably go, “Look that's fine.” Whereas, he's just like, “No, you can do more.” So I just found him pushing me was great.’* |
|  |  | Accountability | *Sue: ‘I think having someone focused on you one on one every week for 12 weeks so that you feel a commitment to make sure you do what you've committed to, I think that really was valuable from that respect.’*  *Mary: ‘Seeing the physiotherapists motivated you to do your stuff, I mean, because they're going to catch you, aren't they? I mean, if you're not improving, or doing it right, they're going to know something's up, not that they're police people but, you know, it just makes perfect sense that if you weren't able to graduate from the small step and you're still at the small step after 3 months, see, I'm now at the big step at the end, so I've gone up to three levels of steps, well, there would be, you know, it's data, and also, too, I don't want to waste their time, or mine.’*  *Louisa: ‘The fact that I was going (to the physiotherapist) was encouraging to me because I had to produce my worksheets and he'd have a look through it and say yes yeah. And you could see what I was doing…. It was also important that I could sort of show the physio that I achieved something you know. And I mean anyone can write in the sheets a figure but you can't cheat the Fitbit.’* |
|  | Motivation and opportunity to exercise | Positive symptom loop | *Bill: ‘The fact that I was getting better and it was improving it the longer I went, that really encouraged me to keep going and I'm determined I'm not going to stop now.’*  *David: ‘And frankly, it gets more enjoyable as the pain diminishes. That just encourages you do (your exercises) more.’*  *Martin: ‘What I found from a pain point of view, the more exercise I was doing, the more activity I was doing, the less I was getting any sort of symptoms.’*  *Hannah: ‘I think when I started to feel a little bit stronger, that my leg was stronger, I then felt more confident that I could do all the exercises.’* |
|  |  | Integration into daily routine | *Jenny: ‘It's all just to and from the station or a walk at lunchtime—it just means I get home a little later. Nothing that inconveniences me at all. I don't have to plan to go out on the weekend and spend hours doing something; it's all incidental type stuff.’*  *Bill: ‘The walking fits in with what I wanted to do anyway so it wasn't a problem… the aerobic exercise has got to be such that it suits a person's particular lifestyle activities.’*  *David: ‘I started off then catching the tram from the station, then to work, now I walk from South Yarra station to St Kilda Road and it's just little things like that I've been able to put incidental movement into my day, and I feel so much better about it.’* |
|  |  | Prior exercise experience | *David: ‘I did like the ones with the weights those but then I've used weights before and I quite like those because I know that it’s just once you get to a particular point you improve.’*  *Brad: ‘I just think if you haven't been used to doing that, I think that's with a lot of things in life, if it's something you've done all your life, if you've played cricket you'll play it in your 50s, but if you just try and take it up in your 50s … It won't work, it'll do your hammie or something. But, you know, it's like a lot of things, if that's what you're used to doing … if your body is conditioned for that you aren't so cautious.’* |
|  |  | Fitbit motivation | *Brad: ‘I think the Fitbit probably helped, it actually became a bit of a, almost a motivation to do more, if I had 3000 steps at the end of work, well, I was probably more likely to get it up to five.’*  *Mary: ‘I love my Fitbit. I mean, I don't have, you know, all those, I don't know much about how to work most of the app bits on there, but I like having my stars when I get to 8000, so it took me, I get to 8000 quicker now than I did in the beginning. Because I only know 8000 because that's when all the rockets go off.’*  *Jenny: ‘The Fitbit probably influenced me a little bit in terms of like the 10,000. Or if it was buzzing at the 250 and I wasn't in a meeting or there wasn't a clear reason, then I probably would be a quick reminder to move.’* |
|  | Time consuming commitment | Frequency of physiotherapy consultations | *Gail: ‘That's hard work seeing someone every week for 12 weeks—because I work full‐time—so just the commitment to do that is not easy.’*  *Jenny: ‘I would actually suggest maybe occasional longer gaps. So, just doing 1 week for 12 weeks. Like some weeks were pretty repetitive or only little changes,* *where probably it might be good to do 6 weeks all together. But then once a month or once a fortnight, then once a month. Just for that spacing out once you've got the hang of it.’* |
|  |  | Travel inconvenience | *Brad: ‘I've found because it's the physiotherapist half hour drive it's sort of not always (easy), it didn't always suit.’*  *Sue: ‘And the only challenge for me was the fact that the physiotherapist was on the other side of town.’*  *Gail: ‘The only downside was really just going to the physiotherapy clinic every week. That was the thing—I just found—it did stress me a little, just with the commute there and back because I'm busy.’* |
|  |  | Time required for exercise | *David: ‘I found it was taking me about 45 min to do the (strengthening) exercises at the end. And that's a bit hard to find in my lifestyle.’*  *Hannah: ‘I suppose we started off very slowly over a short timeframe. There wasn't a huge time impact of how I'm going to fit it all in, but as time has gone on and the walks are taking 20/30 min and the pool work has taken 20/30 min.’*  *Brad: ‘Doing the strengthening 3 times a week—I guess I managed it. I mean people who are working full‐time—I mean I'm running a sort of hospitality business as well. I don't have a lot of hours in my day and I'm planning for this overseas trip as well. So, yeah, I found that it was an impost.’* |
| **Nielsen et al. (2024): “The social and organisational factors shaping acceptability of a self-management education and exercise intervention for people with hip or knee osteoarthritis in Greenland”** | Experiences and perspectives of how the OA School intervention was organised | Acceptability of the hospital as the setting | *For me, it is really good that it is here at the hospital, because it is more committing for me. –Miki* |
|  |  | Acceptability of intervention timings | *(. . .) And I am (job title), so I do not always finish work at four o’clock. I can’t just say, “Now it’s four o’clock, I’m leaving”. And that’s what I had to do, so there were many days when I simply could not make it down there. So it probably started a little after four. I think I would be able to attend if it started at five, for example. –Anna*  *It’s when you work night shifts regularly, you sleep from around 8 in the morning until about 3 in the afternoon. So it’s difficult to fit in such exercises. (. . .) Because it didn’t really match with having to exercise right after waking up. – Ivalu*  *If I want to prioritize exercise more in my daily life, I would prefer to have an easier job, not such a physically demanding one. That way, I would have more energy and time to dedicate to exercising at home. – Kaali* |
|  | Experiences and perspectives of the education and exercise components | Working out with (the right) peers | *It is better when we are several people, and we can encourage each other. I also know some people who have joined the same team as me. So we encouraged each other. Therefore, I think it’s good that it’s a group rather than individual. – Nina*  *I let it fade out because being part of a team was not really my thing. (. . .) Looking back, I think I would have continued if the group consisted mostly of people my age. (. . .) I think what was challenging for me was feeling old and disabled. (. . .) I missed having people on the same level. – Aviaaja*  *When I walked in I thought, “Is this for me?” Because there were only older people, and while some might consider me older in their terminology, I do not see myself as that old yet. There were definitely people on the team who were 20 years older than me. And I thought, “This is not for me”. I would rather go to the gym instead. – Anna* |
|  |  | Acceptability of the physiotherapists | *(. . .) I mentioned it to the physiotherapists, and they said, “Well, try doing it this way”. They are really good at coming up with alternatives if something doesn’t work for you. – Naduk*  *(. . .) It was together with the physiotherapist, so we constantly agreed on what felt good and what didn’t, and then we had to adjust a bit based on how MY knee was feeling. – Nivi*  *(. . .)We received some guidance, but one person had to guide many of us. So I felt like we were left on our own. (. . .) It could also be that there were many newcomers, and there were definitely many people who needed guidance on what they were supposed to do. And I was new to it, so I think I needed more introduction or instruction for the exercises than I received. I felt as if I was left to my own. I was assigned to a stationary bike and such, where I may have had some questions. . . and I did not manage to ask them myself. I could have really used more guidance, I think. – Anna* |
|  |  | Maintaining motivation (over time) | *As a pensioner, one could easily go there three times a week without any major issues, right? But every now and then, you can get a bit stuck in that routine. And in the end, it becomes more like, “Oh, now I have to go there again, right?” It’s like that, in a way. –Malik*  *(. . .) I felt that it was too monotonous. There were no variations in terms of the exercise. –Ivalu*  *It is that someone is there to guide you and make sure you do the right things. They ensure that you get it done. Because I know myself well enough to know that I won’t do it at home. –Miki* |
|  |  | The timing and form of the education session | *It might have been more understandable or better if we had done it (the lecture) at the beginning - so that we knew more about what we were doing. –Malik*  *(. . .) Maybe one could have the instruction online at home BEFORE starting. And then have some questions afterwards. –Nina* |
|  | Significant change stories | Physical improvements | *(. . .) I found it difficult to walk to work because, after walking halfway, my knee would hurt so much that I could barely walk. But now, I can walk to work and even more. The exercise sessions have definitely helped with that. –Nina*  *I can feel the difference by not feeling any discomfort. –Malik* |
|  |  | Improvements in mental health | *(. . .) it changes for the better, and it has made my whole life easier. And of course, when your whole life becomes easier, it also becomes brighter overall, right? It has been really good for me. –Malik*  *One becomes sad because it limits one’s quality of life, but when one receives such help with exercise, one becomes a bit more positive again. I am naturally positive-minded, so I try to see the good in things and make the best of it. –Naduk* |
|  |  | Increased knowledge of OA | *I thought it was a wear and tear of the bones, right? But it wasn’t actually. One is less afraid of having it, you know? –Naduk*  *I think it opened my eyes to a lot of things because it addressed aspects that I had not really thought about in my daily life, but that sometimes arise and I could not find answers to them all of a sudden. And then there’s someone there with the answers. –Malik*  *As a layperson, one does not know much about those things unless they have delved into them for other reasons, so there was a lot of new information for me during the exercise. (. . .) Yes, the idea of moving more to prevent problems was something I could definitely apply. –Miki*  *Yes, I am really happy with it (the new knowledge). I even tell my colleagues, when you go down the stairs or up the stairs, you should do it like this to exercise your knees so that they stay strong as you get older. (. . .) But I am really happy with it. (. . .) Even when I just go up the stairs, I do it very slowly because I feel that they (my knees) need to become stronger. I wasn’t aware of that before. –Naduk* |
