# Supplementary material for: Perceived barriers and facilitators to exercise adherence in osteoarthritis: A thematic synthesis of qualitative studies
Source: Osteoarthr Cartil Open. 2025 Feb 15;7(2):100584. doi: 10.1016/j.ocarto.2025.100584 (PMC11889972; doi:10.1016/j.ocarto.2025.100584)
Supplement: Multimedia component 4 [file mmc4.docx]

**Supplementary Material 4** - Complete Analytical and Descriptive Themes with Codes and Quotations

| ***Analytical Themes*** | ***Descriptive Themes*** | ***Codes*** | ***Quotes*** |
| --- | --- | --- | --- |
| **Mind-Body Connection** | The experience of the exercise programme | Doing the exercise following clear instructions, handbooks, website or wearable devices | “Now I think I handle it more wisely. I know better because I’ve been fortunate to get good instruction” ^21^  “I think, for me, it was 2 separate. The physio [physical therapist] was concentrating and getting me better virtually, and there’s she (the coach) asking how I am managing, and, in a way, yet it goes hand in glove.” ^36^  “Watched me doing all the exercise, then she’d write on the list how many times she wanted me to do, you know. But I said to her, I said, ‘Well I’m doing them once, once a day.’ She said, ‘Well as long as you do thoroughly, but do the ten times of each exercise, you know.” ^53^  “Reassure me. Reassure me. Give me the right exercises to do, if it wasn’t going to do any further damage, if it was arthritis. I think the cartilage problem is caused by, or could have been caused by the arthritis. I needed reassurance that it was okay to actually do the exercises and I wasn’t going to cause further damage. Confidence I think because sometimes trying to do these things on your own is a bit scary if you get stuck, because my leg does lock. And I think probably him encouraging me to do the right exercises, and do them every day, which I did do.” ^53^  “When I was filling out the booklet all the time, that was an incentive to make sure I kept doing the exercises. And I’ve noticed since I haven’t got to fill it out all the time, I’m not doing them 3 times a week, I’ve let it slip…I was using the stepper all the time – yes, I used the stepper every day. Even when I wasn’t doing the exercise and that thing, I was putting it on every day and measuring my daily steps. That was good actually, that was a real motivator.” ^35^  “In addition, she expressed independence to problem solve “…if you had any problem, you had a takehome manual, if you go through that, that would help you…if you forgot the procedure, you know, the right way to stand – or whatever –” ^22^  “That’s what I miss about Joint Academy (digital platform). I have never shown how I do my exercises. So theoretically, I can do them completely wrong.” ^45^  “The stick figures should have been a video instead.— Yes, an instructional film.” ^45^  “… someone must probably instruct me what to do and how to set it up because as I said, I’m not interested in sitting and looking among the apps and what features they have and so on...” ^45^  “More than what the doctor has given you” ^41^ |
|  |  | Knowing exactly what to do | “It got me where I had a wide variety of different exercises that I could do and I felt supported and I knew what I needed to do, so I didn’t really need more [physio consults].” ^35^ |
|  |  | Personalised exercises and progression | “And I think that it is important when people choose which exercises to do, that you enjoy it, that you feel it is rewarding… these positive factors have to be present” ^21^  “The swimming pools are what I would recommend to every person with OA” ^21^  “Sometimes, after a long day, I’ll throw some ice on my knees, take a hot bath after. It feels great… Using the heat was my favorite part of my physiotherapy… That is probably the only way I could handle exercising” ^47^  “I thought, for me, it was beneficial to have both. Initially, I thought no, one’s enough, I don’t need this health coach, but I think putting the 2 together, I think it was beneficial, and it was good. They sort of complemented each other in different ways. Yeah, like I said, one was a pure business-type person, and the other was a very personal person, so they did complement each other, and it worked for me.” ^36^  “They (the exercises) were good because you could just work your way up, you know, and make them harder. They weren’t so difficult that you couldn’t do them and you didn’t want to do them.” ^36^  “I guess certainly the physio treatment and the tailoring of the selection of the exercises and the ability to have that reviewed on a regular basis and ratchet it up accordingly [helped achieve results].” ^35^  “You know, I think there was a couple that, they were hard to do and we just adjusted them and then worked towards the harder ones…I liked that you could build on it, so you weren’t expected to just, you know, do a mammoth effort in the beginning.” ^35^  “With [the physio], we sort of worked on trying to not do it – not go down as far, and so just played that one by ear as to a point where you were continuing to do them, but not to the full degree that it was originally required.” ^35^  “She changed a couple of them because I just said to her, “I can’t squat down on that chair, it just doesn’t happen,” and she did change a couple of them around for me…” ^35^  “I couldn’t do all of the step-downs, because of physical limitation with that, but I certainly tried to do most of them and with the physio, we varied them as much as I could so that I could, you know, get as much out of it as I could with my physical limitations.” ^37^  “The stiff knees tended to cope better and do better and feel better with the [NWBE group]. And the looser knees, more mobile knees, tended to do better with the [WBE] exercises…You’ve just got to find what’s going to work for the patient.” ^37^  “Overall, I think, if you have the right patients, the WBE one is fine and the [NWBE] one might be the person learning the exercises and there isn’t that heavy grinding or clunking feeling when they loaded.” ^37^  “it’s the one step at a time. You do not have to…race at a certain pace. You do it as much as you can.” ^22^  “...while we were in the group each person was able to proceed and progress as they were physically able and mentally able.” ^22^  *“*I just feel fitter and stronger and more confident in everything I do.” ^42^ |
|  |  | Improving your confidence | “…it’s helped me not only with my mobility but my self-confidence to be able to go, yeah, I can get up there all right and come down there.” ^41^  “But we covered the book and it got me to a stage where I was comfortable doing my exercise and there was nothing of concern really…” ^35^  “And it seems like everything I did here [during the exercise class], I was able to do at home with no problems.” ^22^ |
|  |  | Feeling stronger and more flexible | “And so my aim was to…get back to closer to 15 to 20,000 steps a day – which I achieved. So doing the exercises and strengthening the knee, I was able to get back to all of that again…Realistically I knew it was not going to get me back to running.” ^35^  “So the exercises were 1 aspect of it, but the other aspect of it is just understanding my limits, and maybe tapering my expectations a little bit as well.” ^35^  “It did in terms of resistance and reps and stuff, yes, it progressed a lot. I got a lot stronger, definitely, yeah.” ^35^  “I felt all the muscles leading into my knee really – so you know my quad, my hammy, I felt all of that starting to build strength, which was taking a bit of pressure off the poor old knee as well.” ^35^  “You could just definitely feel the strengthening in the quads and things like that, that were actually taking the load off the knee a little bit.” ^35^  “Well, I do feel that it did – I got stronger in the legs, and that was a help.” ^35^  “I felt so much stronger. I could barely walk, I’d use a walker – inside the house, just to go to the sink. And when I first went there I could barely walk and I was doing just a few hundred steps a day…And then I worked up to 6,000 and – I could walk to the sink without my walker. So I definitely got improvement as far as strength went.” ^35^  “It's quite what I needed, and also I found that I wasn't able to… I wasn't able to vacuum and mop the floors in the same—timeframe, so it's a case of mop or vacuum 1 day and not another day. But now I'm back to being able to do it all in one go and that starts the side movement as well.”^39^ |
|  |  | Learning to self-manage and pace | “I still do [the exercises] and I remember to stand the correct way without even thinking about it now . . . . . .. [The pain] has been a lot better, much better, and I can do things better. Dressing—I don’t have to hold on to anything, I can balance now and in fact, you know, I find it a great improvement.” ^52^  “- And that you learn the relationship with how you feel. – Yes, exactly. – Te leg or the knee or the hip or whatever it is... Tat you learn how many steps I must walk so that it does not hurt.” ^45^  “We had it for so long that I felt that at 8,000 [steps] it started to get too tough afterwards, so I tried to stick to it, and I thought it worked well. Ten there was never any [pain]... So previously I activated myself a lot and then nothing... It became a much better rhythm.” ^45^  “An allied health (person) to actually monitor the exercises was not necessary.” ^41^  “And in doing the GLAD program, it made me more aware of what I might be doing in my Pilates and Barre classes and RPM [cycling] classes and things like that...”^42^  “I've learnt a lot about my body and what I can do. I've learnt to watch for trigger signs and what do I do when that happens. So all in all, it has been a positive experience, but a lot of having to adjust to what I felt was working for me.”^39^  “But if I find that my symptoms are coming back I will take from that the understanding that perhaps I need to be strengthening something else and doing one of the other exercises to strengthen a different part of the muscle, et cetera. And I think I understand enough about that now to do that myself.”^39^ |
|  |  | Enjoying exercising | “I exercise in the gym using the machine to bend and straighten my legs with weights and sometimes I use the stationary bike. At home, I would pull my foot up (dorsiflexion) and straighten my leg without weights” ^49^  “It’s part of life, it’s what I do. I get up in the morning, I have a cup of coffee, I take my blood pressure medication, then I go and do my exercises, and then I come back and have breakfast, and that’s just become a routine, which in a way is no different from people going to the gym 3 times a week or doing anything else, so it’s my way of doing things. And I don’t have to leave home!” ^36^  “We were talking about gardening whatever as I was doing my exercises as well. And she sort of mentioned things about her life and what she could do with various bits and pieces and it just made it a much more enjoyable experience I think. [.] I think it made a difference. It made me feel I wanted to do the exercises more.” ^53^ |
|  |  | Getting positive feedback after doing the exercise | “It’s a very good thing if you’ve got their GP onside. So, perhaps it’d be good idea to have some group sessions with the GPs. By group sessions, I don’t mean just getting them in a room like this and talking to them. I mean tell them to come along in their exercise togs and actually do it.” ^37^  It was very often that I looked at [the WAT] and... Oh, ok, so now I have cycled for twenty minutes at a high pace, and I received no credit for it. It’s annoying.” ^45^ |
|  |  | Feeling self-blamed for not exercising, stealing physiotherapists’ time, and not reaching the desired goal | “I really just think it was – I really think it was because I wasn’t doing what I needed to do. I wasn’t – it was mainly in the walking and things like that, I just wasn’t doing it.” ^35^  Joseph: “I think for me it’s more disappointment for not following it through like I should have followed it through I guess…At the end of the day when I did turn up it was really good.” ^35^  Geoffrey: “as I told him [one of the doctors running the trial] really I feel a bit guilty taking his time up because there must be a lot of people a lot worse than what I am.” ^52^ |
|  |  | Comorbidities affecting adherence to exercise | “I get depression, so sometimes I just fall into a big hole and can’t quite function very well. So, we just got through that.” ^37^  “I was really sick for quite a long time and I ended up with pneumonia.” ^37^  “I’ve actually got quite unwell over the years. So, I ended up being in hospital and off work.” ^37^  “Umm, I suppose the things that sort of do prevent you are if you get ill. One thing, that’s probably the only thing would be if I got ill…I probably wouldn’t be able to go, but only that would keep me away.” ^50^  “... The inflammatory conditions that I’ve had with hyperthyroid, the whole package of things that had slowed me down tremendously over the last three or four years...hopefully if my hip settles down...That’s the limiting factor with that now, not my knees...”^42^ |
|  |  | Fatigue | “The [WBE] group would find those exercises more of a mental - mentally tiring. Focusing, concentrating, than actually getting an actual muscular exertion sense…for them it was not about the load on their muscles necessarily, it was about how much cognitive effort it was. Mental effort, for them to do the right alignment.” ^37^  “the NWBE protocol was a lot easier for [patients] to follow [than the WBE]...just because it was less technical.” ^37^  “The effort to get clean afterward is really hard… You just don’t have the energy to take a shower.” ^21^  “Well for me, at first that’s why I missed some of them. I couldn’t go more than one because I was just so tired the next day and would sleep so sound, you know at the night-time, that I couldn’t always wake up early enough to get myself organized to get the bus.” ^50^  ‘I suppose maybe I felt a bit overwhelmed by the combination of strength and aerobic exercise—and that's why I couldn't commit as much to the strengthening as I wanted to—and that's why you come up with all these reasons why you can't do it. So perhaps that was a little bit overwhelming.’^39^ |
|  |  | Complexity of exercises | “the way that I had to attach the weights to my leg, it was just about impossible to do it by myself …if they were a lot easier to use I probably would’ve kept them up a bit more than what I did, but it was just very awkward.” ^37^  “it’s pretty difficult to manoeuvre when it’s not properly strapped in around your ankle… that was probably the hardest part on me, was preparation…It was the reconfiguration of the equipment that weighed on my mind before I said, “Oh, gee, I’ve got to go do that again. I'm going to blow about an hour.” ^37^  “If I was a boy or a man, I would kick those machines; I hate adjusting them, it takes half the time.” ^21^  “the only exercise I wasn’t keen on was - because I could never balance myself very well - the step-up… I always felt that that particular exercise, for me, I probably never really did it correctly.” ^37^  “I liked them all except the straight leg lift; that was the hardest one and still is the hardest one of them all. Very demanding.” ^37^  “The only one I had trouble with was the lightweight with the straight leg, lifting that up. I had a little bit of trouble - It was something to do with my lower back, where your spine goes down and separates towards your buttock.” ^37^ |
|  |  | Stop exercising once feeling better | “I must admit, towards the end, I did flag off a little bit. Mainly because my knee was feeling so good.” ^35^  “I followed exactly what I had to do. Yes, 100 percent.” ^35^  “Since you have stopped seeing [the physiotherapist] have you stopped doing the exercises?” Geoffrey: “Yes I’m sorry I have yes. But as I said I haven’t had no pain . . .. I wondered whether it was temperature or dampness or something like that you see. Now there is nothing wrong with them.” MT: “So you feel if there is nothing wrong with it you feel there is not much point in a...” Geoffrey: “Well that’s it. It’s the wrong attitude I know.” ^52^  “Because my complaints disappeared, I was no longer motivated to continue with the exercises and activities.” ^43^  “I wanted to get rid of the pain. If the pain disappears, why would I bother to continue the exercises? I understand it is better to do the exercises to avoid the pain returning, but, if the pain returns, I will start the exercises again.” ^43^ |
|  |  | Losing the interest in the long run | “The first time I went back it was I pretty much did them all. And then the second time I went back I did, I don’t know, three-quarters. And the third time I went back I did half and sort of dwindled away so by the fifth time I went back it was hardly anything.” ^35^  “At end of treatment was partially adherent to exercises and was an active hill walker. At follow-up no longer did the exercises from the trial but was an active hill walker and had joined a gym.” ^53^  “At end of treatment was adherent to exercise and joined a gym. At follow-up was partially adherent, joined different gym and cycled.” ^53^  “At end of treatment adherent to exercises from the trial and active through dancing. At follow-up continued to dance but not doing exercises from the trial as knee worsened and awaiting knee replacement.” ^53^  “At end of treatment was partially adherent to exercises from the trial, cycled, walked dog and did morning stretches. At follow-up continued to cycle, and walk and did exercises from the trial 2 or 3 times a week.” ^53^  “At end of treatment partially adherent to exercises from the trial. At follow-up partially adherent as tried to do some exercises but others were too painful (due to Baker’s cyst).” ^53^  “At end of treatment was adherent to exercises, withdrawn from study at follow-up.” ^53^  “At end of treatment was adherent to exercises, used exercise bike. At follow-up was not adherent to exercises from the trial but did other sitting exercises, cycled and walked.” ^53^  “At end of treatment partially adherent and did mountain biking and walking. At follow-up partially adherent, did exercises from the trial but irregularly.” ^53^ |
|  |  | Previous negative experiences | “I played sports when I was young, but then I quit. I never had any endurance, so I was never good at it.” ^21^  “I felt like I wanted to do it but couldn’t. So I felt limited by my body rather than attitude. I felt a high degree of frustration with my inability to do things that I wanted to do.” ^42^  ‘I just think if you haven't been used to doing that, I think that's with a lot of things in life, if it's something you've done all your life, if you've played cricket you'll play it in your 50s, but if you just try and take it up in your 50s … It won't work, it'll do your hammie or something. But, you know, it's like a lot of things, if that's what you're used to doing … if your body is conditioned for that you aren't so cautious.’^39^ |
|  |  | Difficulty in starting the exercises | “I think it’s a problem, that you can’t get in… Tat I can’t make it work. I feel it’s like a sort of handicap. But once it works, it’s amazing.” ^45^  “It is good for our age if we learned how to swim but it is hard to learn from our children and we are afraid of water.” ^49^ |
|  |  | Feeling too much pain | “My doctor told me to go on a [recumbent] bicycle for 20 minutes a day, or whatever was easiest for me. So she tells me to pick up my hands [to the sky], which I can’t do because of arthritis in my back. So then she tells me to pick up my legs or do sit ups… But I can’t do those either! I’m so confused. I just find it easier to do nothing.” ^47^  “So it is that maybe when you are old, people back down, they lie on the couch… Surely such a pain affecting someone who does not have that drive [motivation to stay fit] makes people unwilling to get up from the couch” (P1, male, 49) ^48^  “I can’t bend down. I can’t get on the floor, if I do, it is a chore for me to get up. Bending my knees hurts all the time. Walking now seems to be hurting me as well” (P5)^47^  “Not only does it hurt when you [move], but it would hurt the next day. The pain never lets you forget . . . and believe me, I don’t. The only thing I can do is not do it again. Avoid exercise, avoid the pain” (P11) ^47^  “If someone called to play ball or something I would say, “I’m busy, I can’t,” and pretty soon I realized that I couldn’t do it, not that I didn’t want to, I just couldn’t anymore. It wasn’t worth the pain” (P7) ^47^ |
|  |  | Seeing/ not seeing the benefits of continuing to exercise | “The improvement it made… I suppose [I was surprised] that the type of exercise I did could make a difference, I wouldn’t have thought the exercises I was doing would make any difference at all this study taught me that exercise can definitely help with mobility, with arthritis and I think a lot of people including myself were frightened of that, thinking “oh no, I’m going to hurt myself. Going to injure myself. Going to wear out my knees”, you know – it was the opposite effect. The more you move, the better it feels, you know?” ^37^  “If I felt generally more mobile and a bit better.” ^37^  “He was physically incapable of sitting up and within four weeks of that program, particularly the floor exercise, he can get up and he sits. It was just amazing.” ^37^  “It [physical exercise] is not like taking supplements with hyaluronic acid, those (supplements) you do not see what they do.” ^48^ |
|  |  | Personal preferences | “I hate exercise. I have to say, I hate it. I'm one of these people that never go to the gym for exercise.” ^37^  “I’m a person if I start the gym, I go swimming and I do it a few times and I stop. I’m lazy, whatever.” ^37^  “I mean, I'm a bit lazy, I don't really like exercise.” ^37^  “Well, there was this note on the wall saying the aqua-exercise classes are about to start… But for whom?” ^37^  “It is not a purpose in itself to have a digital app, you must be spurred by it as well. So just putting on a Fitbit does not help if you are not interested.” ^45^  “I have always enjoyed physical activity.” ^45^  “The problem was that I never found any that suited me” ^21^  “Yeah, it probably is just a bit boring.” ^36^  “It was boring. Every day, every other day, when you do the same thing, it’s very hard to get motivated, it was a bit boring. Some of the exercises were OK, but some of the exercises… just thought of throwing in the towel virtually, but then I thought the pain versus this, and then it will balance everything out.” ^36^  Stanley: “If perhaps my wife would work with me and you had a bit of competition, but I feel such a fool standing on one leg and going up and down on my own and I tends to drop it I do. I’m not very strong disciplined on that, no. I know some people can be so, but not me. I suppose if there was a really good reason I would.” ^52^ |
|  | Individuals’ mindset  (way of thinking about things) | Recognising that you need to find the time | “It’s not so much the time. I think everyone’s got time. It’s just whether mentally you can get yourself in a frame of actually doing it. That's the issue. I think we all got time. You're kidding yourself if you didn’t.” ^37^  “It’s just excuses when it comes down to basics. I mean you know you could get up in the morning and do it between 6 or 7 or something like that.” ^52^ |
|  |  | Importance of giving a positive narration about an exercise programme | “I think reinforcement of the benefit [is important] because I think they probably have enough information now to say, well, if you do stick to it, if you do it the way you’re supposed to do it, the number of times a week you’re supposed to do it, you will see an improvement. But you can’t, if you go at it half-heartedly then you get a half-hearted result.” ^37^  “I think if it was made clear at outset there was benefits, then I would've probably stuck to it or tried hard to stick to the programme more....I could see it helping but I think a statement at the start would've been helpful.” ^37^  “I think putting a positive spin on it saying that you’re going to improve, so if take part in this program, you’re going to feel better, you’re going to improve, you’re going to walk back, so I think something positive.” ^37^  “I am more vivacious, both physically and mentally” ^21^ |
|  |  | Being positive (mindset) to keep living with OA | “…so I would say my general health was very good. If I’m giving myself a rating out of 10, I would say my general health was a 8.” ^35^  “I would say it’s certainly above average…I have atrial fibrillation which I manage, and then I’ve got the osteoarthritis…other people will say we can’t believe that you’re 70 and your energy and activity and stuff like that.” ^35^  “My general health is fine…I mean, like my knee issues is something that’s been there for a long, long time, so I don’t think my general health affected that program at all; and I’m in good health, touch wood.” ^35^  “My general health is pretty good. I don’t have any major issues.” ^35^  “Again, I think my general health is pretty good. The only thing I’ve got is osteoarthritis in the knees, which I tend to ignore and work around.” ^35^  “I think that general positivism is part of your health; if you think constantly about pain and aches, then you get really sick” ^21^  One becomes sad because it limits one’s quality of life, but when one receives such help with exercise, one becomes a bit more positive again. I am naturally positive-minded, so I try to see the good in things and make the best of it.^51^ |
|  |  | Strong motivation | “You can go too far with this, as you said, you push yourself and then you have to do a little more and then you have to do a little more and you will never be satisfied.” ^45^  “Well I thought it was marvellous really it um you know got us out of bed in the morning and got us into the pool and umm the instructor we had was very, very good and ah I think it was just so good. And I think the motivation was there which is the big thing is to get you motivated you know?” ^50^  “I was very, very diligent with the exercise program certainly through the first 3 months. I didn’t miss a single day. Did all the exercises as required and to the level required.” ^35^  “I felt, as I worked on the exercises – like religiously, doing them every second day.” ^35^  “If you want something bad enough you do it” ^22^  "I found it a little bit intimidating at first [the exercise class], ‘cause it was like an obstacle course, where you had to do this, then you went from that to a [another] thing… I’m saying, “Oh gosh, I can’t do this. This is a bit much.” But then I said, “But no. Let me give it a shot and just try to do it,” and it’s not like I gotta be vigorous with it, just take my time, go at it.” ^22^  “...it will be easy to push or trigger yourself to go those steps extra if you are at 6,500, it is easy to motivate and take another walk to reach the goal.” ^45^  “…Determination and willpower [to change life‐habits]” ^48^  “A great willpower is necessary [to change life‐habits]” ^48^ |
|  |  | No more interest in taking drugs | “I’ve really gone off painkillers, so I rarely take an ibuprofen now and I rarely take anything stronger.” ^37^  June: “I’m a great believer in physiotherapy anyway I think. I don’t agree with drugs quite as much as, I think, if you can have it naturally.” ^52^ |
|  |  | Accommodation is part of OA to keep living your life | Susan: “It’s just there. It’s just part of me now. So, I don’t feel necessarily…oh, I guess, what I feel down about is when I’m with other people and they go for a big long walk, and I say, “I have to sit down, I’ll wait for you here.” So, that’s pretty annoying, but other than that, I just have to accommodate it into my life.” ^35^  “But now I’ve decided to quit driving.” ^21^ |
|  |  | Lack of commitment | “I reckon – quite frankly, I reckon there's a 50/50 chance. And I couldn't commit to anything further than that, because as I said, work is of the utmost – unless, as I said before – unless there's a way in which I can go on sick leave.” ^37^  “I think so, for laziness. Because if you want to, you are able to find the time. So it is, therefore, laziness” ^48^  “Virtually I guess what I’m saying, the problem was probably 100% my lack of 100% commitment rather than any fault of the study.” ^35^ |
|  | The Role of Beliefs  (strong feeling that something is true or real) | Knowing the mechanisms of pain and OA | “Then you get lazy [because of a bad night’s sleep]” (Carl)  “I would like to have an increased support so that you get the whole concept of diet and other things as well, it would have been great, I think.”  It was only during the education that I discovered that exercise is actually the best way to strengthen the joint which takes away – well, in my case, is taking away all the pain.” ^37^  “Just being told that it’s okay to feel pain…I think it was finding that it needed to actually be uncomfortable, nobody had ever said that, they just said you need exercises and I’d sort of slightly cheat, I’d sort of do them but not really do them because I didn’t know what it was meant to feel like” ^37^  “When I was first diagnosed, I didn’t know what to think. I knew it wasn’t good, but I didn’t know how bad it was going to be. After a couple of years, the pain was too much to bear and I thought, that’s it… my life is over. And no one warned me… I didn’t even know what to do… exercise was the farthest thing from my mind.” ^47^  “Someone could help me check what it is that makes me feel so bad today, if it’s because I did too much or I did too little or what could be the cause... Ten I was grateful because I can’t find a pattern myself and don’t really know...” ^45^  “I do not think exercises could worsen my condition” ^49^  “With the knee, I suppose I was imagining there’d be more a hands-on assessment of my knee, and it was just really ‘How are you going with the exercises?’ ‘Are you doing them?’ ‘Aren’t you, and how can you do it so it’s not as painful?’” ^36^  “I think it was just realising that the more you move, the better off you’re going to be, long term.”^42^  I thought it was a wear and tear of the bones, right? But it wasn’t actually. One is less afraid of having it, you know? ^51^ |
|  |  | Exercise will reduce pain after/before surgery or not | “But I imagine that someone can do this… let's call it preventive activity. Activity that can help with the recovery process following the intervention” ^48^  “the knowledge that exercise can help. I had no idea that actually exercise could help like that…that knowledge is the big one that really surprised me” ^37^  “I continue with my exercises, they are integrated in my daily living. I really know these exercises have beneficial effects and that motivates me to continue with my exercises. The main motivation to do all this is to prevent an operation to get a new hip” ^43^  “Well, I – the thing with surgery, it seems to me, is that once the surgery is taking place, and there's been the particular period of physiotherapy, that there's very little pain, as I have heard. Is that going to be the outcome of this sort of course?” ^37^ |
|  |  | An active role will improve OA symptoms and prevent worsening | “I didn’t expect great improvements. All I wanted was to either maintain it, get a little bit better, but not get worse.” ^35^  “I worked out new ways to cope, to keep my arthritis from getting in the way too much” ^21^  “…The body has to move…” ^48^  “I think there is a lot to be learnt and a lot to be done for [arthritis] because even a simple thing like plastering [taping], that is cheap, quick and easy, isn’t it?” ^52^  “I think there’s nothing negative that can happen, even if it didn’t get better, it’s not a negative thing because you tried.” ^35^  “I know exercise is correct. That’s obviously just to strengthen what you have got there and it does work. As far as I don’t know, massage or manipulation or TENS machines, braces and that I don’t know if that makes any difference but I agree with, well, just exercise in general. I know that works.” ^35^ |
|  |  | Strengthening your muscles will improve OA symptoms | “…It’s the answer. If you can’t strengthen those muscles, you’re not going to see any improvement. Without strengthening those muscles, you’re just going to become a couch potato.” ^35^  “…Increasing the muscle strength helps – I don’t know what the term is but it helps support the knee, helps the function in the knee.” ^35^  “But I’m pretty confident that it was, you know, and it was whatever the damage that I had could only be improved by doing strengthening exercises.” ^35^  “You just can’t pop a pill for relief. You have to do other things, other logical things like the exercises and strengthen and what have you.” ^35^  “If you don’t keep your legs stronger – mine aren’t strong enough. I know that, and the more strength you lose in the muscles around – that support your knees, the more limited you become in what your capabilities are and what you can do, and so you lose some. Without the strength in your legs, you lose your life. You lose a desire to go and do things.” ^35^  “It got worse and worse and I started falling down ... Since I started strengthening these muscles it seems I don’t fall over so much which is good ... it’s so embarrassing.” ^52^  “She explained even though the exercises might cause pain, as said, she sort of suggested that the, the problem amongst other things was the lack of strength in the muscle. So she said by building the muscles up that will support the knee better [yeah] in the long run.” ^53^  “OA is difficult to cure, even impossible, it is a natural tear, only some palliatives exist. I believe that the only way, or rather, the best way is to strengthen the muscle structure so that bones and joints suffer less from the weight load on them” ^48^ |
|  |  | Knowing that pain related to exercise is not dangerous | “Well, now I understand it is very important, understanding that a little bit of pain is OK and how to deal and manage that pain and understand that some pain to do with any sort of physical activity is OK and I’m not doing any further damage…” ^35^  “Although I experience the same level of pain, I have learned to continue with my activities and I realise that I achieve more because of that” ^43^ |
|  |  | Knowing that losing weight can help improve OA symptoms | “I think that was half my problems with my knee is because I’m overweight so that doesn’t help in the first place.” ^35^  “I think if I lost 20 kilos, which should be my ultimate game, maybe 30, I suspect my knees would improve out of sight. And I mean, I lost 10 kilos and actually after losing 10, my knees did feel a bit better. So if I lost another 20, I probably think they’d be a lot better.” ^35^  “And then I lost – I can’t remember how much it was now, it was like a fair bit in 6 months – like 8 kilos or something – and I think nothing else would – well, apart from my physical fitness would have been pretty poor – so I think those 2 things. If I could have got more weight off more quickly, I reckon I might have seen more benefits, you know?” ^35^ |
|  |  | No pain no gain / Ignoring pain to keep going | “You ignore pain, that’s the thing too. So it comes and goes. You treat it, you get an antiinflammatory, you get a massage, do what you can and just keep going.” ^35^  “There’s always some pain to have a gain [laughs]. So sometimes doing the exercises, yeah, I would find that there’d be some sort of pain…Yes, it hurts, yes, it’s uncomfortable but hopefully it will keep it moving and going and whatever.” ^35^ |
|  |  | Being confident that exercises will provide benefits | “I was fairly confident that would give at least some benefit.” ^35^  “Once you understand that the program will provide the benefit, then you’ll finish it, but it’s that first one or two sessions and until you’re confident that doing this slightly painful – not necessarily painful, but difficult exercises –- until you’re convinced of that, you won’t complete it. And once people like me that have completed the program, helping the cause to tell other people and other volunteers it’s – that’s the way to get them through. That will give them the encouragement they need.” ^37^  “I cannot let the arthritis overtake you… I was not going to let the arthritis stop me.” ^21^  “I know that when I’m done I feel better. That’s what I’m constantly after.” ^21^  “Exercising has a good effect on everything, including the heart.” ^21^  “All activity is good for sleep.” ^21^  “That is, there were some, just some things [decisions in the care process]… Erm… I don't know… they were left to our intuition, to our perception but just because you understand that by acting in a certain way, maybe you will limit its progress [of OA]…” ^48^  “When I feel better I stop exercising but when the pain increases I register for new sessions” ^49^ |
|  |  | Believing that exercise will not improve as joints are worn out | “The right knee was – worn out, wearing away on the inner side of the joint just because of the structure of my legs so that’s what caused it. My knees are plain old worn out.” ^35^  “And as I said, they’re stuffed anyway. There’s no cartilage in either knee so there’s only so much you can do. So it’s a maintenance – it’s not an improvement program, it’s a maintenance program.” ^35^  “…they’ve worn out. I did 50 years of hairdressing. I’ve done a lot of heavy work in my time like concreting and stuff like that. A lot of gym work which most probably wasn’t real brilliant for them. So I reckon they’re just worn out.” ^35^  “Not only does it hurt when you [move], but it would hurt the next day. The pain never lets you forget . . . and believe me, I don’t. The only thing I can do is not do it again. Avoid exercise, avoid the pain” ^47^  “It definitely wears on you, on your mind because it stops you from doing what you want to do. Even if my body wanted to [exercise], my mind won’t let me… I feel helpless and worthless” ^47^  “I don’t know if exercises could prevent worsening of knees” ^49^  “I don’t know if exercises would improve my knees” ^49^  “[the exercise and taping]might not help me because I’m getting old but it might help somebody else ...I just think I’m too old really to improve.” ^52^ |
|  |  | Believing that exercise will hurt joints | “I had been afraid to exercise because of the pain, and because of the study, I'm now aware that I can actually do something about it rather than just sit on the couch like I had been doing” ^38^  “I wasn’t attempting any exercise on my legs. I wasn’t even going there because I was just too worried about incurring more damage.” ^42^  *“I was a bit hesitant and reluctant too because, of course, my pain I was worried, I’d cause myself an injury.” ^42^* |
|  |  | Believing that OA is due to biomechanical causes | “…the bones now have become weak at the very end of the leg bones, where they would normally be cushioned on the meniscus, so they’ve become soft. And, yeah, it’s just they’re more tender, that’s my understanding of it.” ^35^  “…there must be a link surely that things have to wear out. Just like your car wears out after a certain amount of kilometres.” ^35^  “And I’ve been told that I’ve got bone on bone and I do need a new knee.” ^37^  “He’s just saying this is a one-way ticket, basically. When it gets bad enough, we’ll give you a new knee.” ^37^  “The cartilage, yeah. So I’ve had three arthroscopies and meniscus tears but along that pathway, it was explained to me that I had very little articular cartilage in both knees, so I was expecting that it’s going to be a problem.” ^37^  “Well, I went to sleep and I’m guessing he put something in and scrape – he’s told me, he scraped a lot of arthritis out because there was a lot of broken stuff in there. That’s what he told me.” ^37^  “The doctor told me: “You know that if I did not know that these x‐rays belong to you, I would think that they belong to another person who is at least 30 years older than you”… but, I guess I did not feel as bad as he was describing me.” ^48^  “...exercise in the water, it’s not like walking or running…You’re not jarring any limbs or bones…And for old people I’m, I’m sure that’s the best sort of exercise that you could do.” ^50^  “My GP wasn’t keen on surgery, so their attitude was just keep going and try and avoid it at all costs, whilst my approach is different. I’m trying to fix the issue and particularly at the age I am so I prefer to have it rectified and then I’ll be good for another period of time.” ^37^ |
|  |  | Beliving that OA is part of life | “I was extremely unhappy with myself… I couldn’t work as hard as before, and I just could not understand why. It was one of the hardest things, to accept myself as what I had become.” ^21^  “Well, you have to face the fact that you are not young anymore, and you just have to slow down.” ^21^  “Oh look, I’ve had it for so long, I just, it’s just part of life. It’s a limiter, but just puts boundaries on things.” ^35^  “I just think osteoarthritis is just a part of life. It’s incurable, if that’s the word, and you’ve got to live with it and therefore manage it.” ^35^  “So, unfortunately, it had no effect because I [my hip] is so terribly bad and consequently, I could not walk as much as I would like.” ^45^  “There is nothing that can be done about the OA; therefore, I do nothing.” ^21^  “I am not a one for taking a lot of tablets. I get a bit dubious, you know, so I just learnt to live with it for a bit and then I had the chance of [the trial].” ^35^ |
|  |  | Exercising affects other body areas | “Probably because I didn’t do the exercises. I did some exercises with trepidation for fear of causing my back pain…There was times that I just didn’t do the exercises due to other factors, whatever, at the time.” ^35^  “I thought it was doing me OK but then no, I just couldn’t deal with it anymore…I ended up having to have injections in my hips afterwards. Because I do have bursitis in my hips, so it actually created more problems for me.” ^35^  “Well I think it was the overriding factor – my general health with my hips and my back and that were the major contributor for me easing up and not doing as much as I should have been.”^35^  “I don't know what the exercises involve, but I would have to consider the impact of those on my back.” ^37^  “You really need to have a strong back to start this because you’re going to be doing exercises and your back has to be strong.” ^37^  “I had a shoulder issue at some point and so I just went and saw about that, and I had bursitis of the hips.” ^38^ |
|  |  | Lack of access to information | “There are many 60-year-olds who don’t use computers to get information. And these are the people with arthritis! I think it is much easier to get information to the younger people. We use the Internet.” ^21^  “It is useless to start doing physiotherapy/exercise if I am undertaking surgery in a month.”^48^  “Well nobody knows about the GLA:D program.” ^37^ |
|  |  | Fear of feeling pain | “I’m always in pain and agony, every movement is a chore. Sometimes, I just stare at my stairs, dreading what comes next.” ^47^  “I was advised to walk but if my knees hurt, I would stop walking.” ^49^  “When I did the exercises in the beginning, it wasn’t painful with the tape on, so I think that was how I was able to get on with them so well . . . whereas if the tape came off and I didn’t put it on it was more painful.” ^52^  “I feel much better but I am afraid the pain will be back again once my sessions finish.” ^49^ |
|  |  | Increasing weight can affect OA symptoms | “I feel it right away if I gain a pound; I feel it in my hips and knees.” ^21^  “Well, if you don’t move, you get fat, no matter how little you eat.” ^21^  “Because when you’ve got knees like this, you like to do other things, you think I’m gonna go—I’d like to get back to how I was before, but I don’t think that’s ever going to happen now. I’m sure the weight is the biggest problem . . . . . .I don’t eat as much as I use to, nowhere near and I was slim then. But I love me food so.” ^52^ |
|  |  | No expectations | “I guess when I signed up, I didn’t have an expectation. I thought, you know, anything’s better than nothing.” ^35^  “I was hopeful but I wasn’t unrealistic. So I didn’t expect, I did not expect a miracle.” ^35^  "I think the other reason I probably fell down a little bit on doing the work, the exercises, was that I think after a point I wasn’t convinced that even though I knew strengthening would help, I wasn’t convinced that it would allow me to change my lifestyle back to what it used to be” ^22^ |
| **Social Support Systems** | Relationship with the health professional | Good communication skills to explain exercises in detail | “I think he was really good. He was very easy to talk to, get along with. I think he explained everything really well.” ^35^ |
|  |  | Good listening skills to take on board every aspect and put at ease patients | “[My physical therapist] was really good. Really approachable and listened, and really took on board whatever I said as well.” ^35^  “I must say, in the past I haven’t been all that fussy about physios, because I didn’t feel that they were as hands-on as I would like, but he seemed to be – yeah, sort of easy to talk to and understood the problems quite well.” ^35^  “I mean, he always had time to talk to you, and say, you know, ‘Any questions or anything?’ He didn’t rush you in and rush you out or, like, you know, it does happen sometimes but, with people, but, no, he was very good.” ^53^  “Well, I always say that my physical therapist is as good as any psychologist.” ^21^  “The coaching was very pleasant, very nice conversations in the evening. The physio [physical therapist] was possibly a bit business-minded or a bit focused on the work.” ^36^  “I think she was far more realistic and she seemed more interested in me as a person and what I did. I know the other girl talked about it but it was more as a matter of course, not out of . made you feel particularly valuable I suppose.” ^53^  “I do think it was probably seeing that (trial) physio that really made me open up and think this is a shared thing, he is trying to help me. I wanted to be helped. But he was on such a level that I could share things that I’d maybe found in the past difficult to share.” ^53^  “And then you start asking more... you develop a bit of a rapport and I think that from the study I’m sure the physio gets a little bit more out of it because you start expanding on, on what you’re saying.” ^53^  “So I think, I, I was quite impressed with the physio in that she listened and understood what I was saying with regard to both the pain in the knee and my mental health problems and the hernia.” ^53^ |
|  |  | Being guided by the physiotherapist to understand what to do | “I would like to go to the gym but I am afraid they would not know what I need for my knee. I would prefer to use the gym at the hospital with a physiotherapist.” ^49^  “I would prefer to use the gym at the hospital with a therapist.” ^49^  “The physiotherapist determined the gradual increase of the exercises; he told me, for example, to increase the exercises by five minutes. I liked it that he told me what to do, nevertheless, he was my physiotherapist.” ^43^  “I’d like to know how much attention you’d get because you can be doing something, you can attend the program and be doing it wrong.” ^37^  “It can be a good discussion basis for the follow-up visit: “You have walked far too much” or “you have not moved enough.”” ^45^  “I liked being there because he could look at my technique. I don't know, it's just a bit personable.’” ^39^  “It is that someone is there to guide you and make sure you do the right things. They ensure that you get it done. Because I know myself well enough to know that I won’t do it at home.” ^51^ |
|  |  | Connecting with the physiotherapist | “By going seeing someone every week for a period of time, I think you, you develop some trust, some openness comes from the... from my part, comes from that as well, some understanding.” ^53^  “I felt comfortable with talking to the Physio and built a good rapport with him. It was easier to perform the tasks so that he could see what I was doing.” ^40^ |
|  |  | Fear of being scolded by the physiotherapist | “I guess the fact that I suppose I knew somebody was going to be marking my homework so to speak meant that there was that element as well. If I skip a day or whatever, what’s [my physical therapist] going to say?” ^35^  ^“^The fact that I was going (to the physiotherapist) was encouraging to me because I had to produce my worksheets and he'd have a look through it and say yes yeah. And you could see what I was doing…. It was also important that I could sort of show the physio that I achieved something you know. And I mean anyone can write in the sheets a figure but you can't cheat the Fitbit.” ^39^ |
|  |  | Going to the physiotherapist to do the exercises and be motivated | “I found going to [the physio], it made you do it.” ^35^  “If it wasn’t for a programme that someone was going to use the results, well, I probably would’ve thrown the towel… some sort of motivation to do it is the biggest thing, which is probably having ongoing contact with a physio or something, maybe, cracking the whip sort of thing” ^37^  “I think that physical therapists are the best to help those who have a physical dilemma to start exercising… and start carefully, and under supervision. I think that is very important” ^21^  “The physiotherapist professionally guided me to feel less pain. It made me want to do exercises on my own.” ^47^  “The fact that they were genuinely, or seemed genuinely, interested and were monitoring your progress, you know what I mean, and you sort of go back to your health coach, for example, and you feel a little bit proud that you’ve achieved what you, what’s been set for you, to achieve, and similarly with the physio [physical therapist], you know, I’d say ‘I’ve done it’ with a big smile on my face.” ^36^  “It was just hopeless. She rings up for a chat, and I don’t think she was bossy enough, or clear enough about ‘Now we’re going to have a conversation, and this is what we have to achieve out of this session.’ It was a chat.” ^36^  “There were techniques for how to motivate yourself to do the exercises, and that was good, that was very good, because as I said, knowing that someone’s assessing you, I suppose, makes you more, made me more, responsible for doing the exercises.” ^36^  “The most important thing is listening to the physio [physical therapist] and doing the exercises because he motivated me to do the exercises. It was for my benefit, right, so he kept on pushing me, ‘You have to do it, gradual buildup, don’t go at once, start slowly, work yourself up through the stage,’ and that advice motivated me to do it.” ^36^  “The thing is you do the exercise ‘cause you feel that you don’t want to let the other person down. You know you do them ‘cause in the first instance you think, ‘Oh that’s going to do me good, it’s going to yeah’, but also there’s a secondary thing there you think, ‘Oh he’s gone out of his way to explain these things to me and shown me what to do it’s only fair that I do them so at least I can tell him what sort of effect its having the next time I meet him’, you know.” ^53^  “I’ll be a lot more confident, I think I’ll be a bit, say, worried, but at the moment, I’ve got sort of motivation that I’m being seen sort of every month. Um, just reassurance really, I suppose isn’t it. Um, I’m doing my exercises and I’m going and um, the physio’s going through the exercises with me and telling me this is – well I know it’s improved, but it’s just a bit of reassurance and – and motivation to keep going a bit longer [mmm] with them.” ^53^  “Well I felt because [physiotherapist] took the trouble of explaining it all to me I couldn’t turn around and say, “well blow it, why bother sort of thing? you know?” And when I first turned round and said that I would do it, I felt well alright I wasn’t obligated to do it but I felt let’s do my bit towards it, you know. I didn’t want her to simply think that she was wasting her time.” ^52^  “What keeps me going now is attending physical therapy sessions” ^21^  ‘It's the physical seeing someone that reminds you no, you've not got the angle right or you're not holding enough.’^39^ |
|  |  | Having external support | “I could do it with someone's support - but as soon as the study ended I just sort of dribbled off and I stopped doing it - I can’t seem to self-motivate without that outside support” ^37^  “I was surprised I was committed to it, but part of it was because I felt like I didn’t want to let the program down.” ^37^  ‘It's the physical seeing someone that reminds you no, you've not got the angle right or you're not holding enough.’ ^39^ |
|  |  | Negotiation with the physiotherapist to reach a consensus about the exercises | “I was able to negotiate away from the ones that were awkward or difficult for me, to the ones that were easier or more present or physically possible in my house.” ^35^  “When I first started, it hurt a lot; the first lot of exercises. And it made it that every time I took a step it felt like someone stabbed me in the front of the kneecap with a knife; it was that sharp. And then I went back to [the physio] for my next visit and he changed one of the exercises because it was irritating the knee…and when he changed that one, even though we still did the same exercise but minus the band, it was much better…” ^35^  “The approach of the physiotherapist was very democratic, which I appreciated. Together, we discussed the activities and the increase of the activities. I could indicate to what extent I wanted to increase the activities, to what extent I could maintain the exercises” ^43^  ‘Definitely what worked for my body was the exercises when the physio and I found the ones that were not aggravating pain for me.’ ^39^  “(. . .) It was together with the physiotherapist, so we constantly agreed on what felt good and what didn’t, and then we had to adjust a bit based on how MY knee was feeling.” ^51^ |
|  |  | Physiotherapists showing trust in patients’ capabilities | “[the physical therapist] had, I think, more faith in what I could achieve than what I did first and it was right. I could achieve it because I continued on and trusted him.” ^37^  “I would say that this is a bloody hard exercise and he’d say “well, just get on with it” sort of - I didn’t resent what was going on, it was some parts were difficult.” ^37^ |
|  |  | Physiotherapists pushing patients’ limits | “the physiotherapist challenged me to up the weight, like rather than just keep the same weight on all the time and do more repetitions it was, actually put greater weight on.” ^37^  “.. you have a mentor or a physiotherapist that you meet every three months or when necessary to update your exercises, steps, and the Fitbit. Help with that…Find the level, get that support. – Kind of like a diabetes nurse. - Yes, it might be like that.—OA physiotherapist.” ^45^  ‘It was very helpful having a physio. He just sort of pushed me along a bit, whereas I think if I was doing it at home I’d probably go, “Look that's fine.” Whereas, he's just like, “No, you can do more.” So I just found him pushing me was great.’^39^ |
|  |  | Flexibility to adapt the exercise programme to the patients | “I thought she was excellent. She was good at looking at what was happening and trying to change the program to fit, and I thought that she had a very positive approach…” ^35^  “Yes, I think the instructor was sort of aware of our capabilities and kept the challenge up. And it made it more interesting that way, because if you did the same thing over and over at the same level, it would be boring.” ^50^ |
|  |  | Trusting the physiotherapist | “Certainly, if it’s anything to do with the knee I’ll seek [my PEAK physical therapist] out again and if it’s to do with anything else is a very high probability that I’ll seek him out again.” ^35^  “I thought she was very good. I thought she was very professional. And, yes, I trusted her with what she was telling me to do.” ^35^  “Well, if my specialist had recommended it, I would’ve had my decision validated.” ^37^  “I just trust the professionals what they tell me that it needs.” ^37^  “If I’m happy and I have confidence in a physio that I go to, I will trust that they will do the right thing by me as a patient.” ^37^  “The instructor went through everything with us, and it was like, it was uh, yeah, that’s a challenge I have to do this, I have to do this. I got to do this to try to overcome some of the pains and get some of these stiff joints movin’, you know. Because I had committed myself to ‘em, [exercise] and I had got into a pattern that I was doing them, and I did them.” ^22^ |
|  |  | Need to be dedicated time or listened | “You’re lucky if you’d get a quarter of an hour at our physiotherapist, don’t you?” ^37^  “They [the physicians] are positive if you ask [for a referral to a physical therapist], but you have to ask.” ^21^  “They do not ask if you exercised at home.” ^49^ |
|  |  | Passive solution with no other indication affects trust in patients | “I was told I needed some tablets. And then I went down – I saw the specialist and because the knee was such in a poor state, we virtually jumped straight into surgery.” ^37^  “GP? He doesn’t do anything because he just prescribe the medication but nothing else.” ^37^  “It was me that asked the GP if I could come back here. She did not suggest it. She was into anti-inflammatories.” ^37^  “I said is there anything I can do she said, ‘No, nothing,’ and I think that would be the general answer for most GPs.” ^37^  “I was having trouble with my knees every so often it did hurt you know with one thing and another. Working in the construction industry there is a lot of lifting and a lot kneeling you see and I felt well I wonder if that’s got anything to do with it. So I go to the doctor and all he just simply done was put his hand on my knee, he said “move your leg, . . . you are getting old you’ve got rheumatism.” You see that was it I didn’t take any more notice of it [the knee pain]…” ^52^  “I found that when I didn’t have the tape on I missed it. But I don’t know whether that was psychological or—but I found it helps because when I was walking down the stairs, it was supporting—you know what I mean? But the only trouble with that was, I found that by using it quite so often I used to get a reaction [to the sticking plaster] on my knees, it was sore.” ^52^  “One other physio who’d said to me, “You cannot exercise if it hurts.” ^42^ |
|  |  | Lack of health professionals' support | “They have not done it [encouraged exercising].” ^21^  “The physiotherapist would set up the machine and tell me to exercise then leave.” ^49^  “Only at the end of the last session the physiotherapist told me about the exercises and to do them at home.” ^49^  “I had very little from GPs [general practitioner] or any other professionals. I’d only had a GP do couple of scans or x-rays... and that’s it. No treatment, no exercises, no referrals.” ^42^  “I probably haven’t received any education [about exercises]. I mean the only thing the surgeon ever said to me was, “I’ll see you when you’re ready.”^42^ |
|  | Social Aspects | Preferring to exercise independently | “I like being free when it comes to training time and just decide for myself when I do it and when I don’t.” ^21^ |
|  |  | Having a partner who actively participates in exercising and making new proposals | I’ve always asked her if she thought that this exercise we were doing would work for both of us, or did she need to do something else. She would think of different exercises we could do, and I’d participate with her. I’d think of stuff we could do, and she’ll participate with me.” ^44^  “Not really negotiate…I’ll make the offer and sometimes he joins me and sometimes he doesn’t.” ^44^ |
|  |  | Having a partner or relative who supports and motivates you | “[PALS] would not be worth a dime without your partner.” And later, “…she’s the motivator… She really helps me out.” ^44^  “…we do a lot of it together instead of separate because it’s easier. We find it’s easier if we’re both doing it. Then you both want to go do it. If we try to do it separately, that’s when the old habits creep in. You think of some reason and you do not want to go do it. With the other one it helps motivate you to keep doing it." ^44^  “Sometimes I’ll just start out walking and he’ll see me and come out with me. I do not tell him, ‘Let’s go walking.’ If I see him, I go out with him.” ^44^  “Because it makes a difference. It’s more motivating when you’re doing it [exercise] with someone else. It’s easy to go back to your regular of doing nothing. I mean, if it’s just me, I don’t, I don’t care about me. [laughs] But it’s different, I’d care about someone else.” ^22^  “Yes, my wife, naturally, she encourages me” ^21^  “I like to do physical activities with my wife. We’re together most of the time, so most of it is together.” ^44^  “I prefer to do it with him. It’s easier to do it with him, and that way he’s encouraging me and I’m encouraging him.” ^44^  “…my partner was very supportive in my activity and he encouraged me. ‘Let’s go for a walk after dinner’… instead of, ‘Well no let’s stay home and watch TV’.” ^44^  “No, he did not discourage me one bit. He didn’t do anything to prevent me from being active.” ^44^  “When you’ve got somebody there to encourage you and say, ‘Come on, let’s get it done,’ you know? It helps a lot. Every day you might not feel like getting out of that door, but my wife will say, ‘Come on, get them bones loose, let’s go.’ We stayed at it, and we stay at it.” ^44^  “It [the experience of lack of support] was, just, what should I say, totally pathetic… I guess men are not all equally understanding.” ^21^  “I know [my husband] supports being active, but I never really felt overly supported until my youngest asked if she could come with me on walks. The shocking interest in my exercise habits motivated me to want to exercise more, and be a great role model.” ^47^  “He encourages me in every way.” ^21^ |
|  |  | Mirroring the others to understand if you’re performing the exercises correctly | “I knew exactly what the expectation was in terms of getting it [the exercise] done correctly and for safety’s sake. I certainly didn’t want to be injured. So just being here with you guys watching closely…the movements that I was making, correcting them when [they] needed to be corrected, constantly – it made a big difference, 'cause to ghome and not be sure exactly how to do…the steps or the movements for safety’s sake, would’ve been a cause for concern, I would be like, ‘Am I doing this right?' You know, and that in itself for me is stressful. I don’t want stress.” ^22^ |
|  |  | Exercising in a group is a good motivator | “I think it is the best exercise class I’ve ever attended.” ^21^  “…sometimes being actually on your own to be motivated uh it’s harder. It’s harder: A lot harder.” ^50^  “Anything that will help me continue with it? You put it on and I will be there! Let’s start tomorrow!” ^50^  “Well, some people would, say… “I got fifteen pounds of weight on my [leg],” and I said, “Oh my gosh, how do they do it? I can’t even do four.” [laughs] “And they can do that many?” I said, “Oh, my, maybe I can do that too, eventually” ^22^  “I liked the camaraderie, hearin’ from other people who were experiencing similar problems…it gave me the motivation to also keep doin’ what I was doin’ to stay active.” ^22^  “A suggestion might be to have like a reunion… Do the exercises, …I guess, hearing people’s experiences with the exercises, seeing if we were doing them right, tweaking them, you know, getting some feedback.” ^22^  “I feel that this… I participated in the SOASP… that it was me and then it was 90-year-olds.” ^45^  “It [SOASP] should be sort of more separated in the age groups maybe because I have no one... but it felt like they were not in the same stage as I was. I would probably like to have that.” ^45^  “One of my friends who knows about my arthritis asked me if I ever exercise. “Exercise?!” I said, “What could I do with exercise?!” Then she said she would work out with me if I wanted to. That was the first time I ever seriously thought about exercising.”^47^  “I walked into one of the community centers near my house, and was shocked to see on a bulletin board, they had exercise programs running for people with arthritis, and I just felt, “Hey, I belong here!” ^47^  “I would exercise more if there were group sessions, something social, a pool, something that would not be expensive.” ^49^  “the social side of things is really, really good.” ^50^  “all in the same boat.” ^50^  “Being with the group of elderly people…of same age and we related to so many things that we did, you know. We talked about what helped us and what didn’t help us, you know?” ^50^  “I also think that because they see you trying, it motivates them as well.” ^53^  “It is better when we are several people, and we can encourage each other. I also know some people who have joined the same team as me. So we encouraged each other. Therefore, I think it’s good that it’s a group rather than individual.” ^51^ |
|  |  | Exercising in a group is like having a sense of obligation | “Just having that consultation and someone working with you along the way, there’s a sense of obligation to yourself and to the other.” ^35^  “Well, it’s a commitment, and if you don’t show up, then the group notices.” ^22^  “I really liked the structure of having to show up and go through the exercises.” ^22^  “I like exercising in a group the most… I’m more reluctant to go alone into the gym.” ^21^  “Yeah, so it’s just funny little things that keep you thinking you have a responsibility to attend ‘cause someone’s gonna miss you.” ^50^ |
|  |  | Feeling as a weight for the others | “I’ve got one complaint and it is only really my complaint. It was that most of them could manage so much quicker than me.” ^50^  “He’s more active…He walks faster. He’ll slow down for me, so usually if he walks with me and does stuff for me, that’s really not his routine. He does it with me just to be with me because it makes him happy to see me get out and do some stuff. If he does it with me, it’s more a side thing for him versus a routine.” ^44^ |
|  |  | Exercising only if the partner is exercising too (never alone) | “If I did not do it with her, I do not know if I would do it at all. It is something that we have begun doing together. Although, let me back up. She does it with or without me. She is much more diligent about that than I am. I generally do not do it unless I do it with her.” ^44^ |
|  |  | Having different tastes from the partner can affect the type of exercises they can perform together | “Well, he and I don’t really like many of the same things except for walking…He loves to golf and I picked up golf a little bit but we don’t seem to find the time to do that much together. He definitely doesn’t like Zumba or water aerobics and he doesn’t like swimming at all. Really the only thing we share in common is the walking.” ^44^ |
|  |  | Misunderstanding the partner | He would remind me, every day, to go exercise, and everything, and I always kind of took it the wrong way, that he was saying exercise because you need to lose weight. To hear him say, ‘Don’t forget. You need to exercise today.’ To me, I heard, ‘You are overweight. You need to lose weight. That is what I heard. Which is probably my fault. Well, it is.” ^44^  “Well, she’d get upset, to be honest with you. I mean…that’s something you have to bear. That’s a tough one.” ^44^  “…it’s a little stressful in the marriage anyway. We just got to learn how to communicate with one another. Because when I talk to him he always thinks that I’m nagging or worrying him or whatever or complaining…he’s easy to get upset” ^44^ |
|  |  | Need to have a matching partner in terms of available time, motivation, and preferences | “My husband would not allow me to exercise in a gym.” ^49^  “It’s a lot easier to exercise by myself. Frequently, since we’re both retired, and we both work part time, frequently our schedules don’t match. I end up exercising by myself. When we are at the house together and I’m ready to exercise, more than likely when I say I’m going for a walk he will join me, but, like I said, many times our schedules don’t jibe.” ^44^  “When it comes to physical activity, things that he likes to do that I don’t like to. He likes to go out with his boys and have fun and whatever and I’m just not a ‘going’ person. I just stay at home. We don’t agree…I go to church a lot. If they have an activity at church, I go to the activities at church, but he don’t go to church.” ^44^  “It’s hard to be motivated for two. If she was more motivated, that would be awesome.” ^44^ |
|  |  | Preferring to perform the exercises alone if the partner tries to patronise | “I prefer to do it separately, because I do it at a different pace than my wife does…I kind of push her I kind of wonder if like, make her go a little bit sometimes a little bit too much…a lot of times she would like to do it at her own pace and both of our paces are different.” ^44^  “I prefer separately. Only because my partner tends to want to tell me what to do. I want to do it at my speed, and how I do it…and not be told what to do, like a child.” ^44^ |
| **Environmental Enablers** | Environmental Circumstances | Everywhere is a good spot to exercise | “It was an easy process for me, because I had everything laid out. I kept my bag right there in the kitchen, so while I’m in the kitchen, before I start my day, I would just do my exercises.” ^22^  “…if you didn’t have time to do all of ‘em, you could just start some of them during the day, just as long as you finished it during the day, and I know I would get tired, and I said, ‘Oh no. I forgot to do the lunges,’ you know, that was the last thing, OK. But you know, if I started out in the morning [leaving her home], I was doing them everywhere… I would go quilting, and… I see the stairway [to perform step-up exercises].” ^22^  Alan: “It wasn’t so much at home I am able to do it, it’s more at work . . .. Perhaps not as often as I would really like to, but I can do it quite freely then, because I’m totally on my own.” ^52^ |
|  |  | Financial burden | “Generally most costs because I’m a pensioner, my wife still working though. I have to consider it. And you come to a certain age where your body is falling apart and I need, for example at the moment, another hearing aid. There are lots of things that I need at the moment. Yes financial considerations do matter.” ^21,37^  “And this costs money. Walking, however, is free. Such things matter when you only have your pension” ^21^  “Well, I think that you must always consider the cost of it, I think that's the first one.” ^37^  “Parking around any hospital, not just [Hospital], is a nightmare and you do not want to be in a situation where you have to use the hospital parking because it costs a fortune.” ^37^    “so long as it wasn’t too expensive” ^41^  “Yes… but also from an economic point of view [it is difficult to do supervised exercises]” ^48^  “…couldn’t probably afford a big amount, twice a week” ^50^  “I guess what would hold a lot of people back is the cost. A lot of people just don’t see that as an important thing. I mean they might spend money going out to the pictures every week but a lot of people have a lot of trouble spending on their health, whether it would be buying good healthy food or preventative things and so you try and educate people that, yes, it might cost $500 for the program but the benefits you’ll get.”...” ^42^ |
|  |  | Time burden | “We are trapped into a spiral in which work, we can say, takes up a lot of energy and a lot of time, and then that time is taken away from us…” ^52^  “I have too much work to do, at the end of the day I do not have energy to do the exercises” ^49^  “I like to walk, but I do not because of housework, the children, and winter timing” ^49^  “Weekends I try to do [the exercises] but I am very busy on the weekend really it is the only chance I get to do sort of any cleaning. Then my family usually come up in the afternoon, my sister and her husband, because on the way to Mum’s they always come in. Sunday’s I have Mum on Sunday see, then I have her two sisters because they are older.” ^52^  “I'm hoping to go overseas in five weeks and I'll be away for three weeks. So, I wouldn't be ready to start before eight weeks.” ^37^  “It has to do with the time factor for me - being very busy with work and family often makes me question if I have time for these things, but being online meant I didn’t have to take time from work or family. The care was still of a high standard.” ^40^  ‘That's hard work seeing someone every week for 12 weeks—because I work full‐time—so just the commitment to do that is not easy.’ ^39^  The only downside was really just going to the physiotherapy clinic every week. That was the thing—I just found—it did stress me a little, just with the commute there and back because I'm busy.’ ^39^ |
|  |  | Logistics burden | “I would like to go to the gym but there is not one near my home and my husband would not allow me” ^49^  “They should focus more on exercises. It takes me five hours to get to the hospital and wait for my turn to just have ice packs” ^49^  “So where I lived, it was more than an hour travel to [location] and back and I thought I’d rather spend that hour in the gym.” ^37^  ‘I've found because it's the physiotherapist half hour drive it's sort of not always (easy), it didn't always suit.’ ^39^  “(. . .) And I am (job title), so I do not always finish work at four o’clock. I can’t just say, “Now it’s four o’clock, I’m leaving”. And that’s what I had to do, so there were many days when I simply could not make it down there. So it probably started a little after four. I think I would be able to attend if it started at five, for example”^51^ |
|  |  | Accessibility issues | “Walking upstairs is the worst thing for me” ^21^  “Whether my daughter’s got the time to be taking me to all of this.” ^37^ |
|  |  | Unpredictable life events affecting daily routine | “I persevered with it until a couple of months ago, because I had a lot of bad news in the family and things – stress just took over.” ^35^  “She’d had a car crash. She herself, her leg’s badly damaged. So she had got an insight into sort of what it was all about, you know.” ^53^  “So many things happening ... The boys used to come in from school or work… people come and see [wife] and ugh . . . I’m out twice at least a week to band practice and I have two engagements as well.” ^52^  “There was a time when I missed one [session with the physiotherapist]. I don’t know why . . . . . .I think it was taking the wife some where I don’t know and I must honestly admit that her needs come as a priority as far as I am concerned.” ^52^  “Kept it up right up until most probably just before I left. And then I was packing. So it was more a time factor, and I was – the packing – I had to do most of it myself. And I was really struggling…So there really was no thought of, “Oh yes, I must do my exercises,”  because by the end of the day I could barely move. So yes, I dropped off a fair bit in that few months leading up to moving.” ^35^  “Other things happened in my life that changed as well about the same time. That always complicates outcomes, and they were not something that could be avoided. Just some of my physical activity ceased due to other issues, other people’s injuries, actually. So there was a bit of a sudden change in lifestyle.” ^35^  “...I’m troubled by things like weather and that sort of thing.”^42^ |
|  | Technological support | Wearable devices and monitoring can help you monitor and reach your goal | “I’m amazed at how controlled I am by it, 7,000 Steps, it was like, that’s what I walked every day. And now that I don’t have this [the WAT] anymore, I don’t think I take that many steps anymore. I’m really affected by it.” ^45^  “a little person on my wrist…a little friend.” ^46^  “Happy when I do 10,000 steps…just like a friend supporting me…There’s a gentle persuasion.” ^46^  “There to keep me accountable.” ^46^  Zed found that Fitbit’s influence did “start me going” to meet step goals “in the beginning” of the study, and its influence “wanes over time” once his increase in walking became part of his habitual routine ^46^  “[responding to you mentioned that there were some goals that you didn’t meet…] I don’t feel bad at all. [Laughs] I just kind of go, “Oh that’s life” because I know that I’m keeping really active so yeah it’s not a problem to me. I guess if I thought, “Gee I’m not very active and I’m not meeting any of my goals” then I might feel sad about it but because I know that I like to exercise and so being active is not an issue for me. So the goals I set are kind of like…in a perfect world this is what I would like to do but the world isn’t perfect and it’s okay. You know, I’m working, I’m certainly getting tons of weekly exercise in and so if I don’t meet some aspect of it, it’ll be okay. You know also I guess I can also look at it and kind of go, “If I really, really wanted to do Yoga, I could put a DVD on and do some at home” but again, I like the Yoga for the social aspect so that’s not much of an incentive to do it on my own in my living room…actually I kind of find it a kind of cozy feeling thinking, “I haven’t completed everything. There’s still more to do.” So I’m not taking it kind of like I’m a failure, I’m taking it more as, “Oh there’s still more to do and you can keep on growing, keep on improving” so.” ^46^  “You have a knowledgeable person telling you, you’re doing the right thing type thing…I tried a little bit harder [laughs] maybe for a few weeks…because you actually have another person kind of monitoring you and you also…you want to try…it was very, very encouraging…I think it’s really good for her to be able to see and for me to know that somebody is monitoring me. I think maybe that makes me [laughs] take a few more steps maybe.” ^46^  “I mean I’ve got access to the videos too if I get a little bit stuck.” ^42^  “Never having done care remotely before, I was unsure as to the effectiveness, but it worked well.” ^40^  “I was interested, but couldn’t see how it would work. I was very surprised how well it worked. I put that down to the skill of the physio.” ^40^  “I’m not sure why but I didn’t really see video conferencing as a conventional method of physiotherapy and was not sure how it would work. I was pleasantly surprised how well it works.” ^40^  ‘I think the Fitbit probably helped, it actually became a bit of a, almost a motivation to do more, if I had 3000 steps at the end of work, well, I was probably more likely to get it up to five.’ ^39^ |
|  |  | The role of reminders | “It’s positive that it beeps when you haven’t walked 250 steps in an hour. When it “beeps” you get to move and take a turn in the corridors at work…” ^45^  “…it [the telephone calls] shows that the BOOST Program cared about you. And they wanted to make sure you do your exercise, make sure you was, followin’ the protocol, it was very beautiful.” ^22^  “Even thought I don’t like it, [automated telephone calls] [laughs] it’s a good motivator” ^22^  “I mean, I think that’s something incredibly important, I would love you to keep calling me. And I hate them, I hate the um, the what-do-you-call-it voice. I hate the idea of it [laughs], I mean I, because I [laughs], I hate that the whole thing was happening to us all the time… the automated voices [laughs]. But I really appreciate it.” ^22^  “It [BOOST-TLC] would ask you more specific questions, how many times did you exercise, what were your goals, and I thought that was good. It was kind of a pain in the neck sometimes [laughs]. I said “Oh, I don’t feel like doing this,” but I thought it was good, because it made me think, “OK. When am I gonna exercise?.” ^22^  “I will always keep the Fitbit and always have one I think because it lets me feel as though I’m accomplishing something every day, like I have it set to a pretty low number of steps every day. It’s set to 3000 but when I look at my results I can be over 3000. When it goes off during the day, I feel pretty happy about, “Okay, I’ve accomplished that much today,” and then it’s kind of fun to see how much more I can do.” ^46^  “When I would be close to my steps, if I would see…you just tap it [the Fitbit] and then you’ll know if you’re close or not and I would just make that extra effort to meet that mark. Like instead of driving to work, I would walk to work. On most days it was easy…just seeing that number of how you’re so close. “Got to get over that hump.” Once a goal you’ve set and, you know, when you reach that goal, you feel good about it. It’s just happiness, accomplishment. The other days I’m just in too much pain. I’m like, “I’m not walking home.” I want to be that person going on those hikes. I don’t want to be that person just sitting there. You want to always try to do better the next week but then if it doesn’t happen, I try not to beat myself up over it anymore because, you know, the next day could be better. I’m like, “Okay well you didn’t do well this week. What’s the problem? You shouldn’t be doing that. You shouldn’t be doing this. You should be doing that,” and I just kind of get stuck on that hamster wheel of negative thoughts and have to zip it.” ^46^  “A little text to remind you every now and again doesn’t hurt.” P6 “Yeah, being able to track [physical activity].” ^42^  ‘The Fitbit probably influenced me a little bit in terms of like the 10,000. Or if it was buzzing at the 250 and I wasn't in a meeting or there wasn't a clear reason, then I probably would be a quick reminder to move.’ ^39^  “That was kind of a good little challenge to have your little [activity tracker] on your arm and see how many steps and you know if youneed to go for an extra walk, well, I would.” ^35^  “Knowing that I’m gonna get that phone call!” ^22^ |
|  |  | Devices easy to use | “I’m not the smartest computer user in the world, but if I can do it, I reckon anybody can do it.” ^41^  “It's just simple.” ^41^  “Sometimes with the SMS, I'd put the letters and the things around the wrong way… it was very particular, you know, you had to do it in the right…But other than that, no worries at all.” ^41^ |
|  |  | Wearable tracking your movements can make you understand that you have bad attitudes | “Shows perhaps an inherent bad attitude.” ^46^  “I wasn’t invested that I absolutely had to do, come hell or high water, these steps so I’d be like yeah, I just didn’t walk very much today.” ^46^ |
|  |  | Wearable are considered annoying for the reminders | “It probably gives me some incentive to walk a little further just to placate the Fitbit…I forget to check but I think it does give you an incentive to get out and do something because it’s there and it nags you…I’m fine with that…it’s probably a good thing to have something that makes you get up and go.” ^46^  “I finally get home say around seven in the evening…just kind of want to eat and then just do nothing…I know you’re supposed to move [laughs]…But sometimes I’m just too tired and in the evenings, I’m forced to kind of do some more activities…having the Fitbit it does make me feel I need to move more…I have definitely gone for more walks.” ^46^  “I max out in my activities, so I don’t need this monitoring as a way of positive feedback or gratification to give me incentive. I personally don’t need that…I’m actually walking and all of that. If somebody says, “Oh you should dance more in the evening,” I say, “Well no I can’t dance anymore.” I can only go so far then I drop dead right? So I’m maxed out. I can’t add much more here…So when this six months is over, I’ll take the Fitbit and throw it away because it has no relevance to my life…I know what I’m doing and I don’t care what this little machine tells me.” ^46^  “it was a reminder of the bleeding obvious.” ^41^  “When I got the planter fasciitis and the texts were coming through…they just kept coming, and it was kind of like a little shame thing.” ^41^ |
|  |  | Knowing to be constantly monitored can increase pressure in patients | “But for me, the problem was that I went through a transition. I went through a transition from work, from workin’ in the office, to workin’ at home, and I’m on the phone, and on the computer, and I’m always on the phone all day for, for almost, for pretty much eight hours. So at the end of the day, I’m talked out. I don’t wanna be bothered [with the BOOST-TLC].” ^22^  “It’s just a little bit of pressure of keeping the, the steps they...Because I, I need to watch to keep my promise, you know, as far as I can…she [the physiotherapist] called me like every two weeks. I think her purpose is to motivate me for keeping my, my promise to keep activities.” ^46^  “I do have a grump with the Fitbit over the times where it’s gone into sleep mode so many times…Before I know it I’ve lost 3000 steps…you go like, “I get a lot more steps today than that’s showing me. I know it’s slipped into the sleep mode activity and it’s not”…I figured I should have my entire European boot badge by now so it’s not fair…I knew I was getting a phone call from the physio and I said, “Oh yeah but it wasn’t my fault I didn’t make my 10,000. This stupid band didn’t log on properly.” Oh she said, “Oh yeah, it happens.” ^46^  “At the beginning, it’s very encouraging, but after a while, it’s kind of, to me it’s kind of tedious.” ^22^  “You get disquiet if you do not reach 7,000 steps… I think it happened to me one day and that was very tough…” ^45^ |
